# Membrane fusion and immune evasion by the spike protein of SARS-CoV-2 Delta variant

**DOI:** 10.1126/science.abl9463

**Published:** 2021-10-26

**Authors:** Jun Zhang, Tianshu Xiao, Yongfei Cai, Christy L. Lavine, Hanqin Peng, Haisun Zhu, Krishna Anand, Pei Tong, Avneesh Gautam, Megan L. Mayer, Richard M. Walsh, Sophia Rits-Volloch, Duane R. Wesemann, Wei Yang, Michael S. Seaman, Jianming Lu, Bing Chen

**Affiliations:** 1 Division of Molecular Medicine, Boston Children’s Hospital, Harvard Medical School, 3 Blackfan Street, Boston, MA 02115, USA.; 2 Department of Pediatrics, Harvard Medical School, 3 Blackfan Street, Boston, MA 02115, USA.; 3 Center for Virology and Vaccine Research, Beth Israel Deaconess Medical Center, 330 Brookline Avenue, Boston, MA, 02215, USA.; 4 Institute for Protein Innovation, Harvard Institutes of Medicine, 4 Blackfan Circle, Boston, MA 02115, USA.; 5 Division of Allergy and Clinical Immunology, Department of Medicine, Brigham and Women’s Hospital, Boston, MA 02115, USA.; 6 Ragon Institute of MGH, MIT, and Harvard, Boston, MA 02115, USA.; 7 The Harvard Cryo-EM Center for Structural Biology, Harvard Medical School, 250 Longwood Avenue, Boston, MA 02115, USA.; 8 Department of Biological Chemistry and Molecular Pharmacology, Blavatnik Institute, Harvard Medical School, 240 Longwood Avenue, Boston, MA 02115, USA.; 9 Codex BioSolutions, Inc., 401 Professional Drive, Gaithersburg, MD 20879, USA.; 10 Department of Biochemistry and Molecular and Cellular Biology, Georgetown University School of Medicine, 3900 Reservoir Road, N.W., Washington, D.C. 20057, USA.

## Abstract

The Delta variant of severe acute respiratory syndrome coronavirus 2 (SARS-CoV-2) has outcompeted previously prevalent variants and become a dominant strain worldwide. We report the structure, function, and antigenicity of its full-length spike (S) trimer as well as those of the Gamma and Kappa variants, and compare their characteristics with the G614, Alpha, and Beta variants. Delta S can fuse membranes more efficiently at low levels of cellular receptor angiotensin converting enzyme 2 (ACE2), and its pseudotyped viruses infect target cells substantially faster than the other five variants, possibly accounting for its heightened transmissibility. Each variant shows different rearrangement of the antigenic surface of the amino-terminal domain of the S protein but only makes produces changes in the receptor binding domain (RBD), making the RBD a better target for therapeutic antibodies.

## Delta’s spike

Understanding the molecular mechanisms of the increased transmissibility and immune evasion of severe acute respiratory syndrome coronavirus 2 (SARS-CoV-2) variants is critical to guiding current and future intervention strategies. Zhang *et al*. determined cryo–electron microscopy structures of the full-length spike protein trimers of the Delta, Kappa, and Gamma variants of SARS-CoV-2 and studied their function and antigenic properties. The Delta spike protein fused membranes more efficiently at low levels of the cellular receptor ACE2, and its pseudotyped viruses infected target cells substantially more rapidly than all other variants tested, possibly at least partly accounting for its heightened transmissibility. Mutations of each variant rearranged the antigenic surface of the N-terminal domain of the spike protein but only caused local changes in the receptor-binding domain, consistent with greater resistance to neutralizing antibodies. These findings elucidate the molecular events that have led these viruses to adapt in human communities and to evade host immunity. —VV

## Teaser

Structural and functional studies explain the heightened transmissibility and immune evasion of the SARS-CoV-2 Delta variant.

Severe acute respiratory syndrome coronavirus 2 (SARS-CoV-2) is the causative agent of the COVID-19 pandemic (*1*). The strain responsible for the initial outbreak, Wuhan-Hu-1 (*1*), was the basis for first-generation vaccine development. We previously characterized two early variants of concern (VOC): Alpha and Beta (*2*). The Delta variant (*3*) (also known as lineage B.1.617.2) was first detected in India and was quickly characterized as a VOC and has since outcompeted other variants to become a globally dominant strain within several months. It is estimated to be about twice as transmissible as Wuhan-Hu-1 (*4*, *5*). Infection by the Delta variant appears to have a shorter incubation period with a viral load ~1000 times greater in the first positive Polymerase Chain Reaction (PCR) test than earlier variants (*6*). It remains uncertain whether it causes more severe disease (*7*, *8*), but it does have some resistance to immunity elicited by first-generation vaccines (*9*–*12*). Another VOC, Gamma (lineage B.1.1.28 or P.1), has spread in Brazil and some other countries (*13*, *14*). A third variant, Kappa (lineage B.1.617.1), also first reported in India, remains a variant of interest (VOI) but had only a limited surge (*15*, *16*). It is critical to understand the molecular mechanisms of the increased transmissibility and immune evasion of variants to guide intervention strategies.

SARS-CoV-2 is an enveloped, positive-stranded RNA virus that enters a host cell by fusing its lipid bilayer with the target cell membrane. The fusion reaction is facilitated by the virus-encoded trimeric spike (S) protein after it binds to the host angiotensin converting enzyme 2 (ACE2). The S protein is produced as a single-chain precursor, and processed by a host furin-like protease into the receptor-binding fragment S1 and the fusion fragment S2 (fig. S1) (*17*). After engaging with ACE2 on the host cell surface, the S protein is cleaved by a second cellular protease in S2 (S2’ site; fig. S1) (*18*), initiating S1 dissociation and a cascade of S2 refolding events to drive membrane fusion (*19*, *20*). S1 contains four domains: NTD (N-terminal domain), RBD (receptor-binding domain), and two CTDs (C-terminal domains), protecting the central helical bundle structure of the prefusion S2. The RBD can adopt either a ‘down’ conformation for a receptor-inaccessible state, or an ‘up’ conformation for a receptor-accessible state (*21*); movement of the RBD allows the virus to protect the critical receptor-binding site from host immune responses (*21*, *22*).

Intensive studies on the S protein have advanced our knowledge of SARS-CoV-2 entry substantially (*23*–*26*). Here, we have characterized the full-length S proteins of the Delta, Kappa, and Gamma variants, and determined their structures by cryogenic electron microscopy (cryo-EM). Comparison of the structure, function, and antigenicity of Delta S with those of Gamma and Kappa—as well as the previously characterized Alpha and Beta variants (*2*)—provides molecular insights into the mechanisms of the heightened transmissibility and enhanced immune evasion of the most contagious form of SARS-CoV-2 since its initial outbreak.

## Membrane fusion by Delta S is substantially faster than that of other variants

To characterize the full-length S proteins with the sequences derived from natural isolates of the Gamma (hCoV-19/Brazil/AM-992/2020), Kappa (hCoV-19/India/MH-NEERI-NGP-40449/2021), and Delta (hCoV-19/India/GJ-GBRC619/2021) variants (fig. S1), we transfected HEK293 cells with the respective expression constructs and compared their fusion activities with that of the full-length S construct of their parental strain (G614 or B.1 variant) (*27*). All S proteins were expressed at comparable levels (fig. S2A). Kappa S had <5% cleavage between S1 and S2 compared with ~40% cleavage for other variants at the time the cells were harvested, suggesting that the P681R mutation (found in Delta and Kappa) near the furin cleavage site does not increase furin processing. The extent of cleavage in Delta is not substantially altered from that in its parent strains (fig. S2A). The cells producing these S proteins fused efficiently with ACE2-expressing cells, as expected (fig. S2B). The fusion activity of Kappa S was ~50% that of other S proteins at a low transfection level, presumably as a result of the low furin cleavage, but the difference diminished at high transfection levels (fig. S2B).

**Fig. 1.**
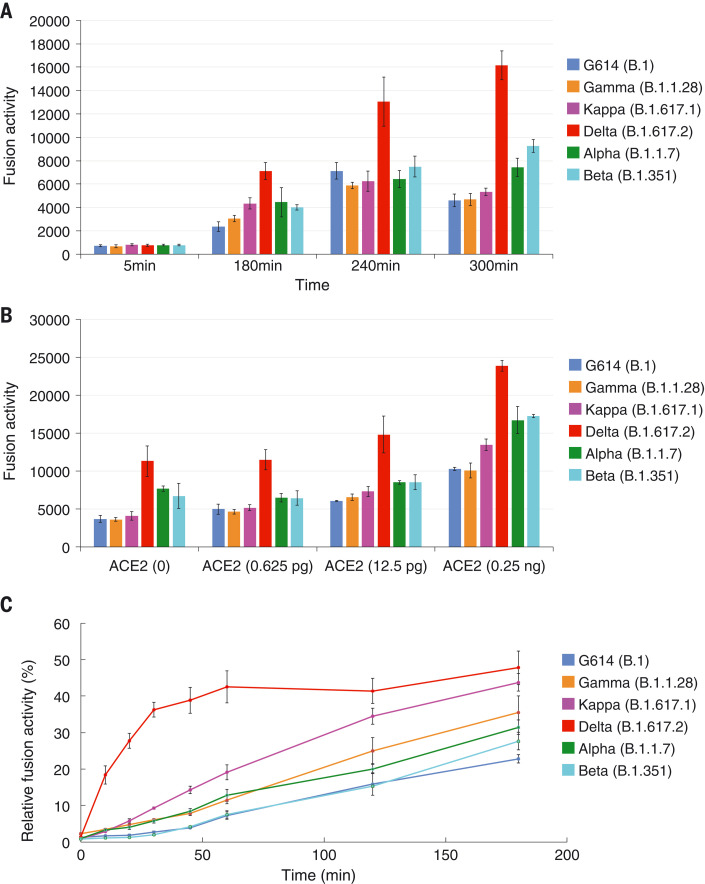
More efficient membrane fusion by the Delta variant than other variants. (**A**) Time course of cell-cell fusion mediated by various full-length S proteins, as indicated by the labels, with HEK293 cells with no exogenous ACE2. (**B**) Cell-cell fusion mediated by various full-length S proteins with HEK293 cells transfected with low levels (0 to 0.25 ng) of ACE2 expression constructs. (**C**) Time course of infection HEK293-ACE2 cells by MLV-based, pseudotyped viruses by various SARS-CoV-2 variant S constructs containing a CT deletion in a single cycle. Infection was initiated by mixing viruses and target cells, and viruses were washed out at each time point as indicated. The full time course and concentration series are shown in fig. S3. The experiments were repeated at least three times, with independent samples each giving similar results.

To test whether more efficient fusion accounts for Delta S transmissibility, we performed a time-course experiment with a cell-cell fusion assay, with both S and ACE2 transfected at high levels (fig. S3A). We found no notable differences in fusion activity among G614, Alpha, Beta, Gamma, Delta, and Kappa. Notably, Delta S-expressing cells fused with the negative-control HEK293 cells more efficiently than other variants, particularly at longer time points (Fig. 1A and fig. S3B). HEK293 cells, expressing a minimal level of endogenous ACE2, are used as negative controls when not transfected by the ACE2 expression construct in our standard 2-hour fusion protocol (*28*). The same pattern was reproduced when small amounts of ACE2 were introduced in HEK293 cells, but the differences diminished when the ACE2 transfection level increased (Fig. 1B and fig. S3C). These data suggest that Delta S can enter a host cell expressing low levels of ACE2 more efficiently than other variants.

We performed a similar time-course experiment by using murine leukemia virus (MLV)–based pseudoviruses expressing the cytoplasmic tail-truncated S constructs to facilitate incorporation into particles (*29*, *30*). The infection was initiated by mixing the viruses and target cells, and the viruses were washed out at each time point. The Delta variant established infection much more rapidly in the first 60-min period than did any other variant, when infectivity was normalized to its maximum level (Fig. 1C). The other variants caught up over time, reaching their maximum levels at 8 hours (fig. S3D). Some viruses—including Delta—reproducibly showed lower measurements for the no wash-out controls than those measured at the 8-hour time point, consistent with some cytotoxicity reducing the reporter-gene expression. Taken together, these findings suggest that the Delta variant can infect a target cell more rapidly than the other variants tested, either by more effective attachment or by faster fusion kinetics.

## Biochemical and antigenic properties of intact S proteins from the variants

We added a C-terminal strep-tag to the full-length S proteins of the Gamma, Kappa, and Delta variants (fig. S4A), and expressed and purified them by the procedures established for the Wuhan-Hu-1 S trimer (*28*). The Gamma protein eluted in three distinct peaks, corresponding to the prefusion S trimer, postfusion S2 trimer, and dissociated S1 monomer, respectively (*28*), as shown by Coomassie-stained SDS-PAGE analysis (fig. S4B). The prefusion trimer accounted for <40% of the total protein, similar to the profile of the Wuhan-Hu-1 protein, indicating that this trimer is not very stable. Although the Kappa protein eluted in one major peak corresponding to the prefusion trimer, there was a considerable amount of aggregate on the leading side and a large shoulder on the trailing side, suggesting that the protein is also conformationally heterogeneous. Moreover, a large fraction of the protein remained uncleaved (fig. S4B), confirming that the furin cleavage is inefficient despite the P681R mutation. By contrast, the Delta protein eluted in a single symmetrical peak of the prefusion trimer showing little aggregation or dissociation, and it appears to be the most stable trimer preparation among all the full-length S proteins that we have examined (fig. S3B) (*31*). Negative stain EM confirmed these results (fig. S5). SDS-PAGE analysis showed that the Delta trimer peak primarily contained the cleaved S1-S2 complex with a cleavage level very similar to that of the G614 and Beta S proteins (*2*, *31*), indicating that P681R has little effect on the furin cleavage.

**Fig. 2.**
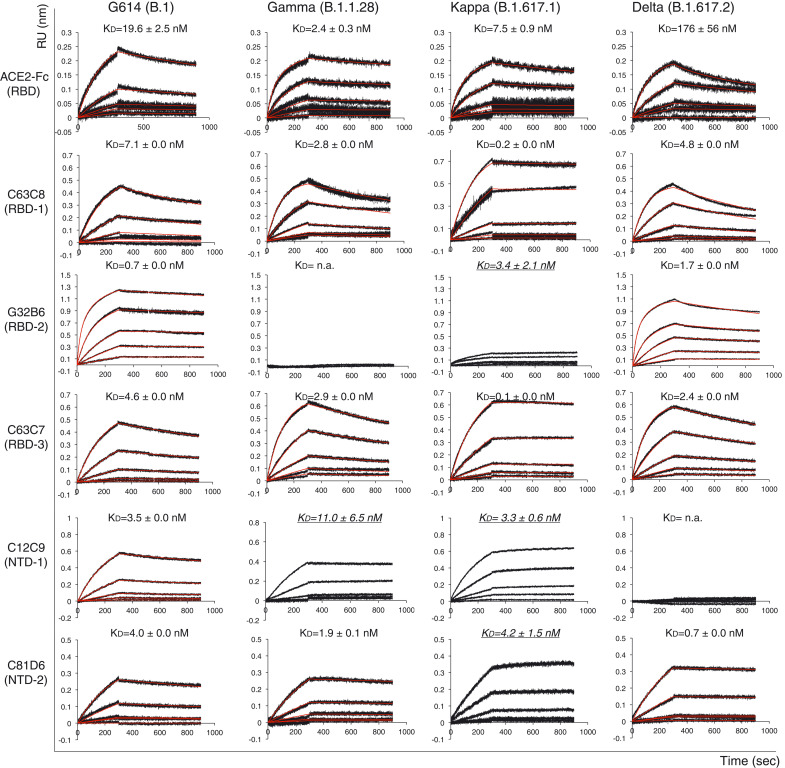
Antigenic properties of purified full-length SARS-CoV-2 S proteins. Biolayer interferometry (BLI) analysis of the association of prefusion S trimers derived from the G614 “parent” strain (B.1) and the Gamma (B.1.1.28), Kappa (B.1.617.1), and Delta (B.1.617.2) variants with soluble ACE2 constructs and with a panel of antibodies representing five epitopic regions on the RBD and NTD (see fig. S4A) (*32*). For ACE2 binding, purified S proteins were immobilized to AR2G biosensors and dipped into wells containing ACE2 at various concentrations. For antibody binding, various antibodies were immobilized to AHC biosensors and dipped into wells containing each purified S protein at different concentrations. Binding kinetics were evaluated by a 1:1 Langmuir model except for dimeric ACE2 and antibody G32B6 targeting the RBD-2, which were analyzed by a bivalent binding model. All K_D_ values for multivalent interactions with antibody IgG or dimeric ACE2 and trimeric S protein are the apparent affinities with avidity effects. Sensorgrams are in black and fits are in red. Binding constants highlighted by underlines were estimated by steady state analysis as described in the materials and methods. RU, response unit. Binding constants are summarized both here and in table S1. All experiments were repeated at least twice with essentially identical results.

To analyze the antigenicity of these S trimers, we measured their binding to soluble ACE2 proteins and S-directed monoclonal antibodies isolated from COVID-19 convalescent individuals by biolayer interferometry (BLI). The selected antibodies recognize distinct epitopic regions on the S trimer, as defined by antibody competition for binding, designated RBD-1, RBD-2, RBD-3, NTD-1, NTD-2, and S2 (fig. S6A) (*32*). The binding of the Gamma variant to the receptor was substantially stronger than that of its G614 parent, regardless of the ACE2 oligomeric state (Fig. 2, fig. S6B, and table S1), likely because of its mutations (K417T, E484K, and N501Y) in the RBD. ACE2 affinities for Kappa and Delta were intermediate between those of the G614 and Gamma trimers, with Kappa and Delta closer to Gamma and G614, respectively, except for binding of Delta with dimeric ACE2, which had an unexpectedly higher off rate than the other variants (Fig. 2). These data were largely confirmed by monomeric RBD preparations instead of the S trimers, except that the Kappa RBD showed slightly higher ACE2 affinity than the Gamma RBD (fig. S6B and table S1). ACE2 did not dissociate more rapidly from the Delta RBD than it did from the Gamma and Kappa RBDs; one possible explanation for the apparently weaker affinity of the Delta trimer for the ACE2 dimer than other variants is that ACE2 binding induces S1 dissociation. These results suggest that the RBD mutations of the Gamma variant enhance receptor recognition, whereas those in Kappa (L452R and E484Q) and Delta (L452R and T478K) have a smaller effect on ACE2 affinity. The dimeric ACE2 appears to be more effective in inducing S1 dissociation from the Delta S trimer than from any other variant.

All selected antibodies had reasonable affinities for the G614 trimer (Fig. 2, fig. S6B, and table S1). The Gamma variant lost binding to the two RBD-2 antibodies (G32B6 and C12A2) and to one NTD-1 antibody (C83B6), but retained binding to the NTD-1 antibody C12C9 with somewhat reduced affinity, suggesting that these two NTD-1 antibodies target overlapping but distinct epitopes (*32*). Its affinities for the remaining antibodies were the same as those of the G614 trimer. Binding of the Kappa trimer showed unrealistically slow off-rates for several antibodies (Fig. 2 and fig. S6B), presumably because of aggregation and conformational heterogeneity. Qualitatively, it had substantially weakened binding to the RBD-2 antibodies and the NTD-1 antibody C83B6, but with wildtype or even enhanced affinity for another NTD-1 antibody (C12C9). Thus, the changes in the Kappa antigenicity show trends similar to those of Gamma S. Delta S only lost binding to the two NTD-1 antibodies with little change in affinities for the other antibodies, including those targeting the RBD (Fig. 2, fig. S6B, and table S1). The BLI data were also largely consistent with the binding results for the membrane-bound S trimers measured by flow cytometry (fig. S7).

We next analyzed the neutralization potency of these antibodies and trimeric soluble ACE2 (*33*) by measuring the extent to which they blocked infection by S variants in an HIV-based pseudovirus assay. For most antibodies, the neutralization potency correlated with their binding affinity for the membrane-bound or purified S proteins (table S2). C81D6 and C163E6 recognized two nonneutralizing epitopes in the NTD-2 and S2, respectively, and did not neutralize any of the pseudoviruses. Thus, the mutations in the Gamma and Kappa variants have a greater effect on antibody sensitivity than those in the Delta variant.

## Overall structures of the intact S trimers of the Delta, Kappa and Gamma variants

**Fig. 3.**
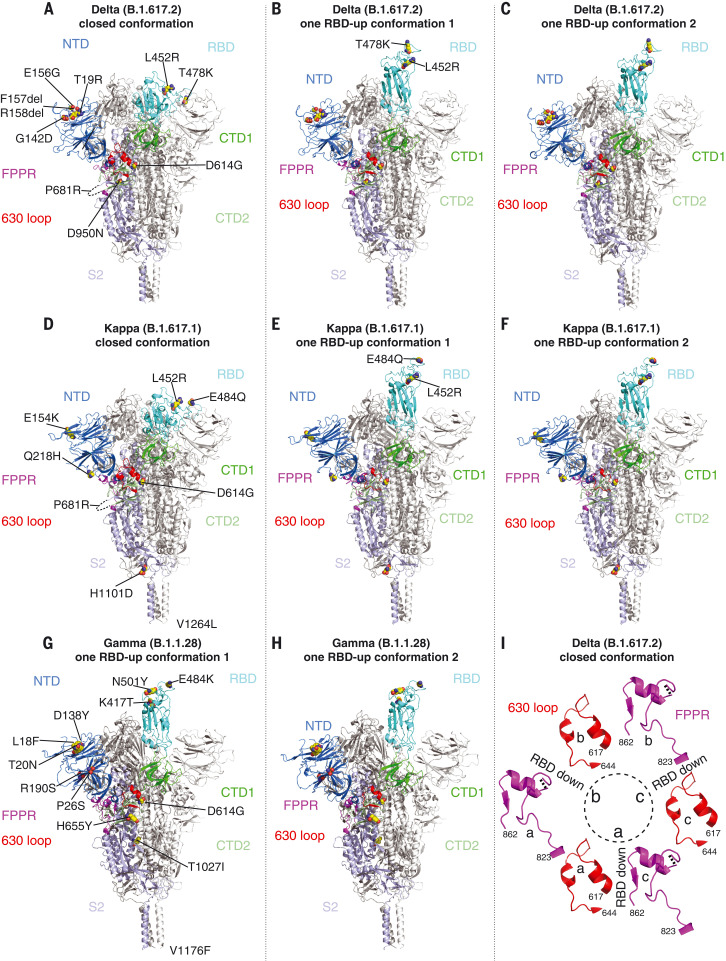
Cryo-EM structures of full-length SARS-CoV-2 S proteins from the Delta, Kappa, and Gamma variants. (**A** to **C**) The structures of the closed prefusion conformation and two one-RBD-up conformations of the Delta S trimer are shown in the ribbon diagrams, with one protomer colored as follows: NTD in blue, RBD in cyan, CTD1 in green, CTD2 in light green, S2 in light blue, the 630 loop in red, and the FPPR in magenta. (**D** to **F**) The structures of the closed prefusion conformation and two one-RBD-up conformation of the Kappa S trimer are shown in the ribbon diagrams, with the same color scheme as in (A). (**G**) and (**H**) The structures of the two one-RBD-up conformations of the Gamma S trimer are shown in the ribbon diagrams with the same color scheme as in (A). All mutations in the three variants, as compared to the original virus (D614), are highlighted in the sphere model. (**I**) Structures in the Delta closed trimer of segments (residues 617 to 644) containing the 630 loop (red) and segments (residues 823 to 862) containing the FPPR (magenta) from each of the three protomers (A), (B), and (C). The position of each RBD is indicated. Dashed lines indicate gaps in the chain trace (disordered loops).

We determined the cryo-EM structures of the full-length S trimers with the unmodified sequences of the Delta, Kappa, and Gamma variants, according to our established procedures (*2*, *28*, *31*). 3D classification gave three distinct classes each for both the Delta and Kappa trimers, representing one closed prefusion conformation and two one-RBD-up conformations, respectively. There were two classes for the Gamma trimer, representing two one-RBD-up conformations. These structures were refined to 3.1 to 4.4 Å resolution (figs. S8 to S14 and table S3). There are no major changes in the overall architectures of the full-length variant S proteins when compared with that of the parental G614 S trimer in the corresponding conformation (Fig. 3 and fig. S15) (*31*). The furin cleavage site (residues 682 to 685) at the S1-S2 boundary, including the P681R substitution, was not visible in these maps.

We have proposed that the FPPR (fusion peptide proximal region; residues 828 to 853) and 630 loop (residues 620 to 640) are control elements, and that shifts in their positions modulate the S stability and structural rearrangements (*28*, *31*). For the Delta and Kappa variants, the FPPR and 630 loop configurations are largely consistent with the distribution in the G614 trimer; all are structured in the RBD-down conformation, whereas only one FPPR and 630-loop pair is ordered in the one-RBD-up conformations. The density for FPPR residues 841 to 847 of the Delta S in the closed state is weak, probably because of slight (1 to 2Å) downward shifts of the CTD1 and RBD, which may weaken the FPPR packing (fig. S15). No class representing the closed conformation was identified for Gamma S from three independent datasets (fig. S12), suggesting this conformation is not well occupied by that variant; however, one FPPR and 630-loop pair is structured in the one-RBD-up conformations of Gamma, probably stabilizing the cleaved S trimers before receptor engagement. In all three variants, the distinct one-RBD-up structures differ only by the degree to which the up-RBD and the adjacent NTD of its neighboring protomer shift away from the central threefold axis (fig. S15). An N-linked glycan at Asn^343^ has been implicated in a gating role for facilitating RBD opening (*34*). Its density is stronger in the maps of all the new variants, particularly Delta and Kappa, than that in the G614 map (fig. S16). The distal end of this glycan contacts the neighboring RBD, forming a ring-like density and apparently stabilizing the three-RBD-down conformation. Nonetheless, it remains unclear why the Gamma prefusion trimer dissociates, the Kappa trimer tends to aggregate, and the Delta trimer is the most stable of the three.

## Structural consequences of mutations in the Delta variant

**Fig. 4.**
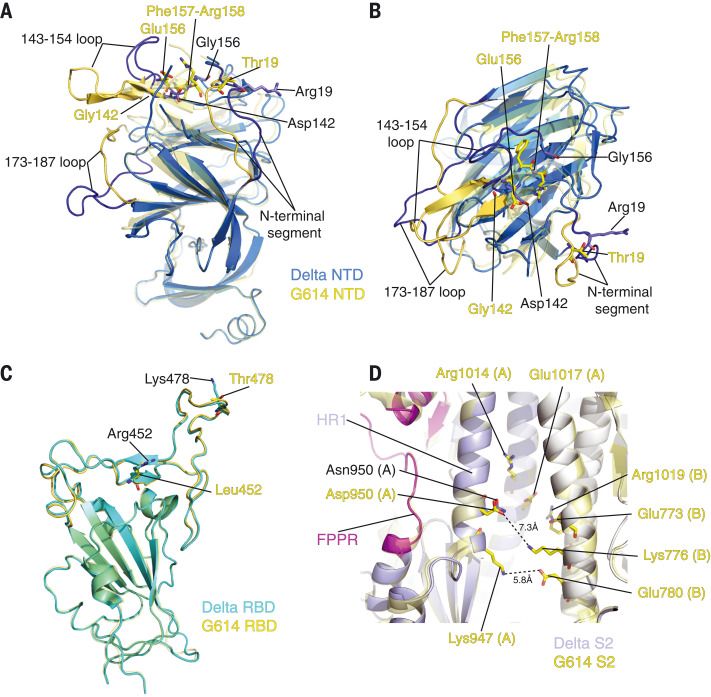
Structural impact of mutations in Delta S. (**A**) Superposition of the NTD structure of the Delta S trimer (blue) with the NTD of the G614 S trimer (PDB ID: 7KRQ) (yellow). Locations of mutations T19R, G142D, E156G, and deletion of F157 and R158 are indicated; these residues are shown in the stick model. The N-terminal segment, as well as loops 143 to 154 and 173 to 187, are rearranged between the two structures and highlighted in darker colors. (**B**) Top view of panel (A). (**C**) Superposition of the RBD structure of the Delta S trimer (cyan) with the RBD of the G614 S trimer (yellow). Locations of mutations L452R and T478K are indicated; these residues are shown in the stick model. (**D**) A close-up view of superposition of the Delta S2 (light blue) with the S2 of the G614 S trimer (yellow) near residue 950. Locations of the D950N mutation and charged residues in the vicinity including Lys^947^, Arg^1014^, and Glu^1017^ from protomer A and Glu^773^, Lys^776^, Glu^780^, and Arg^1019^ from the protomer B are indicated. All aforementioned residues are shown in the stick model.

We superposed the structures of the Delta S trimer onto the G614 trimer in the closed conformation, aligning them by the S2 region (fig. S15) and revealing the most prominent differences in the NTD, which contains three point mutations (T19R, G142D, and E156G) and a two-residue deletion (F157del and R158del). When the two NTDs are aligned (Fig. 4A and 4B), the mutations reshape the 143-154 loop, which contains an N-linked glycan (N149) and forms part of the NTD-1 epitopes (*35*–*38*), projecting it away from the viral membrane. They also reconfigure the N-terminal segment and the 173-187 loop, substantially altering the antigenic surface near the NTD-1 epitopes, and consistent with loss of binding and neutralization by NTD-1 antibodies (Fig. 2, fig. S6, and table S2). There are no major structural rearrangements in the Delta RBD with two mutations, L452R and T478K (Fig. 4C). These residues are not in the ACE2 contacting surface, and have little influence on the receptor binding (fig. S17) (*39*). Neither binding nor neutralization of the Delta variant by most anti-RBD antibodies tested here have changed, suggesting that the two residues are also not in any major neutralizing epitopes. No obvious structural alterations were observed from the D950N substitution in HR1 (heptad repeat 1) of S2 (Fig. 4D), with multiple pairs of charged residues in the vicinity that could stabilize the packing between S2 protomers in the prefusion conformation.

## Structural impact of the mutations in the Kappa and Gamma variants

**Fig. 5.**
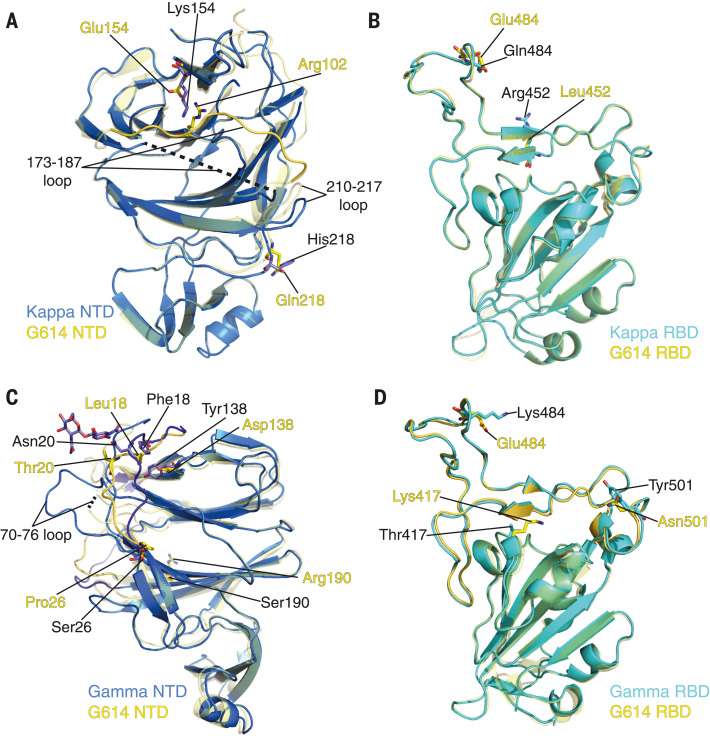
Structural impact of mutations in the Kappa and Gamma S proteins. (**A**) Superposition of the NTD structure of the Kappa S trimer (blue) with the NTD of the G614 S trimer (yellow). Locations of mutations E154K and Q218H, as well as Arg^102^ which forms a salt bridge with Glu^154^ in the G614 structure, are indicated; these residues are shown in the stick model. The 173 to 187 loop in the G614 trimer is highlighted in a darker color; it becomes disordered in the Kappa trimer. (**B**) Superposition of the RBD structure of the Kappa S trimer (cyan) with the RBD of the G614 S trimer (yellow). Locations of mutations L452R and E484Q are indicated; these residues are shown in the stick model. (**C**) A view of superposition of the NTD structures of the Gamma (blue) and G614 (yellow; PDB ID: 7KRR) S trimers in the one-RBD-up conformation. Locations of mutations L18F, T20N, P26S, D138Y, and R190S are indicated, as well as the N-linked glycan attached to Asn^20^ in the Gamma structure; these residues are shown in the stick model. (**D**) Superposition of the RBD structure of the Gamma S trimer (cyan) with the RBD of the G614 S trimer (yellow). Locations of mutations K417T, E484K, and N501Y are indicated, and these residues are shown in the stick model.

There are only two mutations (E154K and Q218H) in the Kappa NTD (Fig. 5A). Glu^154^ forms a salt bridge with Arg^102^ in the G614 trimer (*31*); E154K substitution results in an unfavorable interaction with Arg^102^, possibly leading to a disordered 173 to 187 loop nearby in the Kappa trimer. Residue 218 is surface-exposed and on the opposite side from the neutralizing epitopes. Q218H may contribute to rearrangement of the 210 to 217 and 173 to 187 loops (Fig. 5A). There are two RBD mutations (L452R and E484Q) in Kappa (Fig. 5B), which do not alter the overall structure of the domain. Glu^484^ forms a salt bridge with ACE2 Lys^31^ in the RBD-ACE2 complex (fig. S17) (*40*, *41*). The E484Q substitution loses the salt bridge, but hydrogen bonds between Gln^484^ and ACE2 Lys^31^ might compensate and thus account for a small increase in ACE2 binding affinity. L452R is unlikely to substantially affect ACE2 binding (fig. S17). The mutation H1101D in S2 caused little local change (fig. S18A), and V1264L is not visible in our structures.

Structural changes in the Gamma NTD caused by the mutations (L18F, T20N, P26S, D138Y, and R190S) were evident in the EM maps (fig. S14). All mutations except for R190S are located near the N-terminal segment and contribute to reconfiguration of its extended structure (Fig. 5C). The new conformation of the N-terminal segment appears to stabilize the 70 to 76 loop, disordered in most known S trimer structures (*21*, *28*, *42*). T20N has created a new glycosylation site and Asn^20^ is indeed glycosylated in Gamma (Fig. 5C). These changes apparently also shift the 143 to 154 and 173 to 187 loops (fig. S18B), leading to relatively large-scale rearrangement of the antigenic surface of the Gamma NTD. The three RBD mutations (K417T, E484K, and N501Y) in Gamma also produce no major structural rearrangements (Fig. 5D). N501Y increases receptor-binding affinity, which may be counteracted by K417T and E484K because of loss of ionic interactions with ACE2 (fig. S17) (*2*, *43*–*46*). K417T and E484K are probably responsible for loss of binding and neutralization of Gamma by antibodies that target the RBD-2 epitopes (*45*, *47*, *48*). H655Y in the CTD2 did not change the local structure (fig. S18C), but its location near the N terminus of the cleaved S2 suggests a role in destabilizing the Gamma S trimer. Finally, T1027I did not lead to any major changes in S2 (fig. S18D), and V1176F is in a disordered region.

The Delta variant of SARS-CoV-2 has rapidly replaced the previously dominant variants—including Alpha—which is itself ~60% more transmissible than the Wuhan-Hu-1 strain (*49*–*51*). Delta thus appears to have acquired an enhanced capacity for propagating in human cells. Several hypotheses have been proposed to explain its heightened transmissibility, including mutations in the RBD enhancing receptor engagement (*52*), P681R substitution near the S1-S2 boundary leading to more efficient furin cleavage (*53*, *54*), and changes in its RNA polymerase increasing viral replication. We cannot rule out the possibility that mutations in the viral replication machinery characteristic of Delta (e.g., G671S in nsp12) may increase the production of genomic RNA, but viral assembly into mature virions would require many other factors to achieve the >1,000-fold greater viral load in infected patients. We have not detected any notable increase in ACE2 binding by either the full-length Delta S trimer or its RBD fragment, nor have we observed more efficient cleavage in the Delta S than any other variants. Indeed, the furin cleavage is already very efficient in G614, Alpha, Beta, and Delta (*2*, *31*, *54*), and may no longer be a rate-limiting step for all these variants.

We have identified two properties, so far only found in the Delta variant, that might account for its transmissibility. First, when the Delta S protein is expressed on the cell surface at a saturating level, those cells fuse more efficiently with target cells that produce lower levels of ACE2 than do cells of any other variant (*2*). When the ACE2 expression level increases, the differences among the variants diminish. Second, the pseudoviruses containing the Delta S construct enter the ACE2-expressing cells more rapidly than other variants. These data suggest that the Delta S protein has evolved to optimize the fusion step for entering cells expressing low levels of the receptor. This optimization may explain why the Delta variant can transmit upon relatively brief exposure and infect many more host cells rapidly, leading to a short incubation period and greater viral load during infection. One caveat is that all our experiments were performed in vitro; additional studies with authentic viruses will be needed to confirm our findings in more clinically relevant settings.

The RBD and NTD are the two major sites on the S trimer targeted by neutralizing antibodies (*32*, *36*, *55*, *56*). The three strains studied here show once again how different variants can use different strategies to remodel their NTD and evade host immunity. One notable implication is that the NTD function does not require specific structural elements or sequences because the surface loops, β strands in the core structure, and even some N-linked glycans can be rearranged in different ways without compromising viral infectivity. By contrast, the overall structure of the RBD has been strictly preserved among all variants, and reoccurring surface mutations appear to be limited to a number of sites, consistent with its critical role in receptor binding. We therefore suggest that therapeutic antibodies or universal vaccines should not target the NTD, as escape from anti-NTD antibodies appears to be of little cost to the virus.

## Supplementary Material

20211026-1

## Supplementary Materials

### This PDF file includes:

Materials and Methods Figs. S1 to S18 Tables S1 to S3 References (*57*–*69*)

### Other Supplementary Material for this manuscript includes the following:

MDAR Reproducibility Checklist

## Notes

View/request a protocol for this paper from *Bio-protocol*.
