# Supplementary material for: Membrane fusion and immune evasion by the spike protein of SARS-CoV-2 Delta variant
Source: Science. 2021 Oct 26;374(6573):1353–60. doi: 10.1126/science.abl9463 (PMC10763652; doi:10.1126/science.abl9463)
Supplement: 20211026-1 [file science.abl9463.v1.pdf]

Cite as: B. Chen *et al.*,
Science 10.1126/science.abl9463 (2021).

Membrane fusion and immune evasion by the spike protein of SARS-CoV-2 Delta variant

Jun Zhang[1,2]†, Tianshu Xiao[1,2]†, Yongfei Cai[1,2], Christy L. Lavine[3], Hanqin Peng[1], Haisun Zhu[4], Krishna Anand[4], Pei Tong[5,6], Avneesh Gautam[5,6], Megan L. Mayer[7,8], Richard M. Walsh Jr.[7,8], Sophia Rits-Volloch[1], Duane R. Wesemann[5,6], Wei Yang[4]‡, Michael S. Seaman[3], Jianming Lu[9,10], Bing Chen[1,2]*

[1]Division of Molecular Medicine, Boston Children's Hospital, Harvard Medical School, 3 Blackfan Street, Boston, MA 02115, USA. [2]Department of Pediatrics, Harvard Medical School, 3 Blackfan Street, Boston, MA 02115, USA. [3]Center for Virology and Vaccine Research, Beth Israel Deaconess Medical Center, 330 Brookline Avenue, Boston, MA, 02215, USA. [4]Institute for Protein Innovation, Harvard Institutes of Medicine, 4 Blackfan Circle, Boston, MA 02115, USA. [5]Division of Allergy and Immunology, Department of Medicine, Brigham and Women's Hospital, Boston, MA 02115, USA. [6]Ragon Institute of MGH, MIT and Harvard, Boston, MA 02115, USA. [7]The Harvard Cryo-EM Center for Structural Biology, Harvard Medical School, 250 Longwood Avenue, Boston, MA 02115, USA. [8]Department of Biological Chemistry and Molecular Pharmacology, Blavatnik Institute, Harvard Medical School, 240 Longwood Avenue, Boston, MA 02115, USA. [9]Codex BioSolutions, Inc., 401 Professional Drive, Gaithersburg, MD 20879, USA. [10]Department of Biochemistry and Molecular and Cellular Biology, Georgetown University School of Medicine, 3900 Reservoir Road, N.W., Washington, D.C. 20057, USA.

†These authors contributed equally to this work.

‡Present address. GV20 Therapeutics LLC, 1 Broadway Floor 14, Cambridge, MA 02142, USA.

*Corresponding author. Email: bchen@crystal.harvard.edu

The Delta variant of severe acute respiratory syndrome coronavirus 2 (SARS-CoV-2) has outcompeted previously prevalent variants and become a dominant strain worldwide. We report the structure, function, and antigenicity of its full-length spike (S) trimer and those of the Gamma and Kappa variants and compare their characteristics with the G614, Alpha, and Beta variants. Delta S can fuse membranes more efficiently at low levels of cellular receptor ACE2, and its pseudotyped viruses infect target cells substantially faster than the other five variants, possibly accounting for its heightened transmissibility. Each variant shows different rearrangement of the antigenic surface of the N-terminal domain of the S protein, but only causes local changes in the receptor-binding domain (RBD), making the RBD a better target for therapeutic antibodies.

Severe acute respiratory syndrome coronavirus 2 (SARS-CoV-2) is the causative agent of the COVID-19 pandemic (*1*). The strain responsible for the initial outbreak, Wuhan-Hu-1 (*1*), was the basis for first-generation vaccine development. We previously characterized two early variants of concern (VOC) Alpha and Beta (*2*). The Delta variant (*3*), first detected in India and also known as lineage B.1.617.2, was soon characterized as a VOC and has outcompeted other variants to become a globally dominant strain within several months. It is estimated to be about twice as transmissible as Wuhan-Hu-1 (*4*, *5*). Infection by the Delta variant appears to have a shorter incubation period with a viral load around 1000 times greater at the first positive PCR test than earlier variants (*6*). It remains uncertain whether it causes more severe disease (*7*, *8*), but it does have some resistance to immunity elicited by the first-generation vaccines (*9*–*12*). Another VOC, Gamma (lineage B.1.1.28 or P.1), has spread in Brazil and some other countries (*13*, *14*). A third variant, Kappa (lineage B.1.617.1), also first reported in India, remains a variant of interest (VOI) but had only a limited surge (*15*, *16*). It is critical to understand molecular mechanisms of the increased transmissibility and immune evasion of variants to guide intervention strategies.

SARS-CoV-2 is an enveloped, positive-stranded RNA virus that enters a host cell by fusing its lipid bilayer with the target cell membrane. The fusion reaction is facilitated by the virus-encoded trimeric spike (S) protein after it binds to the host angiotensin converting enzyme 2 (ACE2). The S protein is produced as a single-chain precursor, and processed by a host furin-like protease into the receptor-binding fragment S1 and the fusion fragment S2 (fig. S1) (*17*). After engaging with ACE2 on the host cell surface, the S protein is cleaved by a second cellular protease in S2 (S2′ site; fig. S1) (*18*), initiating S1 dissociation and a cascade of S2 refolding events to drive membrane fusion (*19*, *20*). S1 contains four domains: NTD (N-terminal domain), RBD (receptor-binding domain), and two CTDs (C-terminal domains), protecting the central helical bundle structure of the prefusion S2. The RBD can adopt either a "down" conformation for a receptor-inaccessible state, or an "up" conformation for a receptor-accessible state (*21*); its movement allows the virus to protect the critical receptor-binding site from host immune responses (*21*, *22*).

Intensive studies on the S protein have advanced our knowledge of the SARS-CoV-2 entry substantially (*23*–*26*). Here, we have characterized the full-length S proteins of the Delta, Kappa, and Gamma variants, and determined their structures by

cryogenic electron microscopy (cryo-EM). Comparison of the structure, function and antigenicity of the Delta S with those of Gamma and Kappa, and previously characterized Alpha and Beta (*2*), provides molecular insights into mechanisms of the heightened transmissibility and enhanced immune evasion of the most contagious form of SARS-CoV-2 since its initial outbreak.

Membrane fusion by Delta S is substantially faster than that of other variants

To characterize the full-length S proteins with the sequences derived from natural isolates of Gamma (hCoV-19/Brazil/AM-992/2020), Kappa (hCoV-19/India/MH-NEERI-NGP-40449/2021) and Delta (hCoV-19/India/GJ-GBRC619/2021) variants (fig. S1), we transfected HEK293 cells with the respective expression constructs and compared their fusion activities with that of the full-length S construct of their parental strain (G614 or B.1 variant) (*27*). All S proteins expressed at comparable levels (fig. S2A). Kappa S had <5% cleavage between S1 and S2 as compared with ~40% cleavage for other variants at the time when the cells were harvested, suggesting that the P681R mutation (found in Delta and Kappa) near the furin cleavage site does not increase furin processing. The extent of cleavage in Delta is not significantly altered from that in its parent strains (fig. S2A). The cells producing these S proteins fused efficiently with ACE2-expressing cells, as expected (fig. S2B). The fusion activity of Kappa S was ~50% that of other S proteins at a low transfection level, presumably due to the low furin cleavage, but the difference diminished at high transfection levels (fig. S2B).

To test whether more efficient fusion accounts for Delta S transmissibility, we performed a time-course experiment with a cell-cell fusion assay, with both S and ACE2 transfected at high levels (fig. S3A). We found no significant differences in the fusion activity among G614, Alpha, Beta, Gamma, Delta, and Kappa. Delta S-expressing cells fused with the negative-control HEK293 cells more efficiently than other variants, however, particularly at longer time points (Fig. 1A and fig. S3B). HEK293 cells, expressing a minimal level of endogenous ACE2, are used as negative controls when not transfected by the ACE2 expression construct in our standard, 2-hour fusion protocol (*28*). The same pattern was reproduced when small amounts of ACE2 were introduced in HEK293 cells, but the differences diminished when the ACE2 transfection level increased (Fig. 1B and fig. S3C). These data suggest that Delta S can enter a host cell expressing low levels of ACE2 more efficiently than other variants.

We performed a similar time-course experiment using murine leukemia virus (MLV)-based pseudoviruses expressing the cytoplasmic tail-truncated S constructs to facilitate incorporation into particles (*29*, *30*). The infection was initiated by mixing the viruses and target cells, and the viruses were washed out at each time point. The Delta variant established infection much more rapidly in the first 60-min period than did any other variant, when infectivity was normalized to its maximum level (Fig. 1C). The other variants caught up over time, and reached their maximum levels at 8 hours (fig. S3D). Some viruses, including Delta, reproducibly showed lower measurements for the no wash-out controls than those measured at the 8-hour time point, consistent with some cytotoxicity reducing the reporter-gene expression.

Together these findings suggest that the Delta variant can infect a target cell substantially more rapidly than the other variants we tested, either by more effective attachment or faster fusion kinetics.

Biochemical and antigenic properties of the intact S proteins from the variants

We added a C-terminal strep-tag to the full-length S proteins of the Gamma, Kappa and Delta variants (fig. S4A), and expressed and purified them by the procedures established for the Wuhan-Hu-1 S trimer (*28*). The Gamma protein eluted in three distinct peaks, corresponding to the prefusion S trimer, postfusion S2 trimer, and dissociated S1 monomer, respectively (*28*), as shown by Coomassie-stained SDS-PAGE analysis (fig. S4B). The prefusion trimer accounted for <40% of the total protein, like the profile of the Wuhan-Hu-1 protein, indicating that this trimer is not very stable. Although the Kappa protein eluted in one major peak corresponding to the prefusion trimer, there is a significant amount of aggregate on the leading side and a large shoulder on the trailing side, suggesting that the protein is also conformationally heterogeneous. Moreover, a large fraction of the protein remains uncleaved (fig. S4B), confirming that the furin cleavage is inefficient despite the P681R mutation. By contrast, the Delta protein eluted in a single symmetrical peak of the prefusion trimer showing little aggregation or dissociation, and it appears the most stable trimer preparation among all the full-length S proteins that we have examined (fig. S3B) (*31*). Negative stain EM confirmed these results (fig. S5). SDS-PAGE analysis showed that the Delta trimer peak contained primarily the cleaved S1/S2 complex with a cleavage level very similar to that of the G614 and Beta S proteins (*2*, *31*), indicating little impact of P681R on the furin cleavage.

To analyze antigenicity of these S trimers, we measured their binding to soluble ACE2 proteins and S-directed monoclonal antibodies isolated from COVID-19 convalescent individuals by biolayer interferometry (BLI). The selected antibodies recognize distinct epitopic regions on the S trimer, as defined by which antibodies compete for binding, designated RBD-1, RBD-2, RBD-3, NTD-1, NTD-2, and S2 (fig. S6A) (*32*). The Gamma variant bound substantially more strongly to the receptor than did its G614 parent, regardless of the ACE2 oligomeric state (Fig. 2, fig. S6B, and table S1), probably because of its mutations (K417T, E484K, and N501Y) in the RBD. ACE2 affinities for Kappa and Delta were intermediate between those of the G614 and Gamma trimers, with Kappa closer to Gamma and Delta closer to G614, except for binding of Delta with dimeric ACE2, which had an unexpectedly higher

off-rate than the other variants (Fig. 2). These data were largely confirmed using monomeric RBD preparations instead of the S trimers, except that the Kappa RBD showed slightly higher ACE2 affinity than the Gamma RBD (fig. S6B and Table S1). ACE2 did not dissociate more rapidly from the Delta RBD than it did from the Gamma and Kappa RBDs; a possible explanation for the apparently weaker affinity of the Delta trimer for the ACE2 dimer than other variants is that ACE2 binding induces S1 dissociation. These results suggest that the RBD mutations of the Gamma variant enhance receptor recognition, while those in Kappa (L452R and E484Q) and Delta (L452R and T478K) have a lesser impact on ACE2 affinity. The dimeric ACE2 appears to be more effective in inducing S1 dissociation from the Delta S trimer than from any other variant.

All selected antibodies had reasonable affinities for the G614 trimer (Fig. 2, fig. S6B, and table S1). The Gamma variant lost binding to the two RBD-2 antibodies, G32B6 and C12A2, and to one NTD-1 antibody, C83B6, but it retained binding to the NTD-1 antibody C12C9 with somewhat reduced affinity, suggesting that these two NTD-1 antibodies target overlapping but distinct epitopes (*32*). Its affinities for the remaining antibodies were the same as those of the G614 trimer. Binding of the Kappa trimer showed unrealistically slow off-rates for several antibodies (Fig. 2 and fig. S6B), presumably because of aggregation and conformational heterogeneity. Qualitatively, it had substantially weakened binding to the RBD-2 antibodies and the NTD-1 antibody C83B6, but with wildtype or even enhanced affinity for another NTD-1 antibody C12C9. Thus, the changes of its antigenicity show similar trends to those of the Gamma S. The Delta S only lost binding to the two NTD-1 antibodies with little changes in affinities for the other antibodies, including those targeting the RBD (Fig. 2, fig. S6B, and table S1). The BLI data were also largely consistent with the binding results for the membrane-bound S trimers measured by flow cytometry (fig. S7).

We next analyzed the neutralization potency of these antibodies and of trimeric soluble ACE2 (*33*) by measuring the extent to which they blocked infection by S variants in an HIV-based pseudovirus assay. For most antibodies, the neutralization potency correlated with their binding affinity for the membrane-bound or purified S proteins (table S2). C81D6 and C163E6 recognize two nonneutralizing epitopes in the NTD-2 and S2, respectively; they did not neutralize any of the pseudoviruses. Thus, the mutations in the Gamma and Kappa variants have a greater impact on antibody sensitivity than those in the Delta variant.

Overall structures of the intact S trimers of the Delta, Kappa and Gamma variants

We determined the cryo-EM structures of the full-length S trimers with the unmodified sequences of the Delta, Kappa, and Gamma variants, following our established procedures (*2*, *28*, *31*). 3D classification gave three distinct classes each for both the Delta and Kappa trimers, representing one closed prefusion conformation and two one-RBD-up conformations, respectively. There were two classes for the Gamma trimer, representing two one-RBD-up conformations. These structures were refined to 3.1 to 4.4 Å resolution (figs. S8 to S14 and table S3). There are no major changes in the overall architectures of the full-length variant S proteins when compared with that of the parental G614 S trimer in the corresponding conformation (Fig. 3 and fig. S15) (*31*). The furin cleavage site (residues 682-685) at the S1/S2 boundary, including the P681R substitution, was not visible in these maps.

We have proposed that the FPPR (fusion peptide proximal region; residues 828 to 853) and 630 loop (residues 620 to 640) are control elements and that shifts in their positions modulate the S stability and structural rearrangements (*28*, *31*). For the Delta and Kappa variants, the FPPR and 630 loop configurations are largely consistent with the distribution in the G614 trimer: all are structured in the RBD-down conformation, while only one FPPR and 630-loop pair is ordered in the one-RBD-up conformations. The density for the FPPR residues 841-847 of the Delta S in the closed state is weak probably because of slight (1 to 2Å) downward shifts of the CTD1 and RBD, which may weaken the FPPR packing (fig. S15). No class representing the closed conformation was identified for the Gamma S from three independent datasets (fig. S12), suggesting this conformation is not well occupied by that variant, but one FPPR and 630-loop pair is structured in the one-RBD-up conformations of Gamma, probably stabilizing the cleaved S trimers before receptor engagement. In all three variants, the distinct one-RBD-up structures differ only by the degree to which the up-RBD and the adjacent NTD of its neighboring protomer shift away from the central threefold axis (fig. S15). An N-linked glycan at Asn343 has been implicated in a gating role for facilitating RBD opening (*34*). Its density is stronger in the maps of all the new variants, particularly Delta and Kappa, than that in the G614 map (fig. S16). The distal end of this glycan contacts the neighboring RBD, forming a ring-like density and apparently stabilizing the three-RBD-down conformation. Nonetheless, it remains unclear why the Gamma prefusion trimer dissociates, the Kappa trimer tends to aggregate, and the Delta trimer is the most stable of the three.

Structural consequences of mutations in the Delta variant

We superposed the structures of the Delta S trimer onto the G614 trimer in the closed conformation, aligning them by the S2 region (fig. S15) and revealing the most prominent differences in the NTD, which contains three point mutations (T19R, G142D, and E156G) and a two-residue deletion (F157del and R158del). When the two NTDs are aligned (Fig. 4A and 4B), the mutations reshape the 143-154 loop, which contains an N-linked glycan (N149) and forms part of the NTD-1 epitopes (*35*–*38*), projecting it away from the viral membrane. They also reconfigure the N-terminal segment and the 173-187 loop, substantially altering the antigenic

surface near the NTD-1 epitopes, and consistent with loss of binding and neutralization by NTD-1 antibodies (Fig. 2, fig. S6, and table S2). There are no major structural rearrangements in the Delta RBD with two mutations, L452R and T478K (Fig. 4C). These residues are not in the ACE2 contacting surface, and have little impact on the receptor binding (fig. S17) (*39*). Neither binding nor neutralization of the Delta variant by most anti-RBD antibodies tested here have changed, suggesting that the two residues are also not in any major neutralizing epitopes. No obvious structural alterations were observed from the D950N substitution in HR1 (heptad repeat 1) of S2 (Fig. 4D), with multiple pairs of charged residues in the vicinity that could stabilize the packing between S2 protomers in the prefusion conformation.

Structural impact of the mutations in the Kappa and Gamma variants

There are only two mutations (E154K and Q218H) in the Kappa NTD (Fig. 5A). Glu154 forms a salt bridge with Arg102 in the G614 trimer (*31*); E154K substitution results in an unfavorable interaction with Arg102, possibly leading to a disordered 173-187 loop nearby in the Kappa trimer. Residue 218 is surface-exposed and on the opposite side from the neutralizing epitopes. Q218H may contribute to rearrangement of the 210-217 and 173-187 loops (Fig. 5A). There are two RBD mutations (L452R and E484Q) in Kappa (Fig. 5B), which do not alter the overall structure of the domain. Glu484 forms a salt bridge with ACE2 Lys31 in the RBD-ACE2 complex (fig. S17) (*40*, *41*). The E484Q substitution loses the salt bridge, but hydrogen bonds between Gln484 and ACE2 Lys31 might compensate and thus account for a small increase in ACE2 binding affinity. L452R is unlikely to significantly impact ACE2 binding (fig. S17). The mutation H1101D in S2 caused little local change (fig. S18A), and V1264L is not visible in our structures.

Structural changes in the Gamma NTD caused by the mutations (L18F, T20N, P26S, D138Y and R190S) were evident in the EM maps (fig. S14). All mutations except for R190S are near the N-terminal segment and contribute to reconfigure its extended structure (Fig. 5C). The new conformation of the N-terminal segment appears to stabilize the 70-76 loop, disordered in most known S trimer structures (*21*, *28*, *42*). T20N has created a new glycosylation site and Asn20 is indeed glycosylated in Gamma (Fig. 5C). These changes apparently also shift the 143-154 and 173-187 loops (fig. S18B), leading to a relatively large-scale rearrangement of the antigenic surface of the Gamma NTD. The three RBD mutations (K417T, E484K, and N501Y) in Gamma also produce no major structural rearrangements (Fig. 5D). N501Y increases receptor-binding affinity, which may be counteracted by K417T and E484K because of loss of ionic interactions with ACE2 (fig. S17) (*2*, *43*–*46*). K417T and E484K are probably responsible for loss of binding and neutralization of Gamma by antibodies that target the RBD-2 epitopes (*45*, *47*, *48*). H655Y in the CTD2 did not change the local structure (fig. S18C), but its location near the N terminus of the cleaved S2 suggests a role in destabilizing the Gamma S trimer. Finally, T1027I did not lead to any major changes in S2 (fig. S18D), and V1176F is in a disordered region.

The Delta variant of SARS-CoV-2 has rapidly replaced the previously dominant variants, including Alpha, which is itself ~60% more transmissible than the Wuhan-Hu-1 strain (*49*–*51*). Delta thus appears to have acquired enhanced capacity for propagating in human cells. Several hypotheses have been proposed to explain its heightened transmissibility, including mutations in the RBD enhancing receptor engagement (*52*), P681R substitution near the S1/S2 boundary leading to more efficient furin cleavage (*53*, *54*), and changes in its RNA polymerase increasing viral replication. We cannot rule out the possibility that mutations in the viral replication machinery characteristic of Delta (e.g., G671S in nsp12) may increase the production of genomic RNA, but viral assembly into mature virions would require many other factors to achieve the >1,000-fold greater viral load in infected patients. We have not detected any significant increase in ACE2 binding by either the full-length Delta S trimer or its RBD fragment, nor have we observed more efficient cleavage in the Delta S than any other variants. Indeed, the furin cleavage is already very efficient in G614, Alpha, Beta, and Delta (*2*, *31*, *54*), and may no longer be a rate-limiting step for all these variants.

We have identified two properties, so far only found in the Delta variant, that might account for its transmissibility. First, when the Delta S protein is expressed on the cell surface at a saturating level, those cells fuse more efficiently with target cells that produce low levels of ACE2 than do cells of any other variant (*2*). When the ACE2 expression level increases, the differences among the variants diminish. Second, the pseudoviruses containing the Delta S construct enter the ACE2-expressing cells more rapidly than other variants. These data suggest that the Delta S protein has evolved to optimize the fusion step for entering cells expressing low levels of the receptor. This optimization may explain why the Delta variant can transmit upon relatively brief exposure and infect many more host cells rapidly, leading to a short incubation period and greater viral load during infection. A caveat is that all our experiments were performed in vitro; additional studies with authentic viruses will be needed to confirm our findings in more clinically relevant settings.

The RBD and NTD are the two major sites on the S trimer targeted by neutralizing antibodies (*32*, *36*, *55*, *56*). The three strains studied here show once again how different variants can use different strategies to remodel their NTD and evade host immunity. One notable implication is that the NTD function does not require specific structural elements or sequences since the surface loops, β strands in the core structure and even some N-linked glycans can be rearranged in different ways without compromising viral infectivity. By contrast, the overall structure of the RBD has been strictly preserved among all the variants and reoccurring surface mutations appear to be limited to a number of sites, consistent

with its critical role in receptor binding. We therefore suggest that therapeutic antibodies or universal vaccines should not target the NTD since escaping from anti-NTD antibodies appears to have little cost to the virus.

REFERENCES AND NOTES

1. P. Zhou, X.-L. Yang, X.-G. Wang, B. Hu, L. Zhang, W. Zhang, H.-R. Si, Y. Zhu, B. Li, C.-L. Huang, H.-D. Chen, J. Chen, Y. Luo, H. Guo, R.-D. Jiang, M.-Q. Liu, Y. Chen, X.-R. Shen, X. Wang, X.-S. Zheng, K. Zhao, Q.-J. Chen, F. Deng, L.-L. Liu, B. Yan, F.-X. Zhan, Y.-Y. Wang, G.-F. Xiao, Z.-L. Shi, A pneumonia outbreak associated with a new coronavirus of probable bat origin. *Nature* **579**, 270–273 (2020). doi:10.1038/s41586-020-2012-7 Medline

2. Y. Cai, J. Zhang, T. Xiao, C. L. Lavine, S. Rawson, H. Peng, H. Zhu, K. Anand, P. Tong, A. Gautam, S. Lu, S. M. Sterling, R. M. Walsh Jr., S. Rits-Volloch, J. Lu, D. R. Wesemann, W. Yang, M. S. Seaman, B. Chen, Structural basis for enhanced infectivity and immune evasion of SARS-CoV-2 variants. *Science* **373**, 642–648 (2021). doi:10.1126/science.abi9745 Medline

3. P. Mlcochova *et al*., SARS-CoV-2 B.1.617.2 Delta variant replication, sensitivity to neutralising antibodies and vaccine breakthrough. *bioRxiv*, 2021.2005.2008.443253 (2021).

4. R. Earnest, R. Uddin, N. Matluk, N. Renzette, K. J. Siddle, C. Loreth, G. Adams, C. H. Tomkins-Tinch, M. E. Petrone, J. E. Rothman, M. I. Breban, R. T. Koch, K. Billig, J. R. Fauver, C. B. F. Vogels, S. Turbett, K. Bilguvar, B. De Kumar, M. L. Landry, D. R. Peaper, K. Kelly, G. Omerza, H. Grieser, S. Meak, J. Martha, H. H. Dewey, S. Kales, D. Berenzy, K. Carpenter-Azevedo, E. King, R. C. Huard, S. C. Smole, C. M. Brown, T. Fink, A. S. Lang, G. R. Gallagher, P. C. Sabeti, S. Gabriel, B. L. MacInnis, R. Tewhey, M. D. Adams, D. J. Park, J. E. Lemieux, N. D. Grubaugh, Comparative transmissibility of SARS-CoV-2 variants Delta and Alpha in New England, USA. *medRxiv*, 2021.10.06.21264641 (2021) https://doi.org/10.1101/2021.10.06.21264641.

5. J. Dagpunar, Interim estimates of increased transmissibility, growth rate, and reproduction number of the Covid-19 B.1.617.2 variant of concern in the United Kingdom. *medRxiv*, 2021.2006.2003.21258293 (2021).

6. B. Li *et al*., Viral infection and transmission in a large well-traced outbreak caused by the Delta SARS-CoV-2 variant. *medRxiv*, (2021) doi:10.1101/2021.07.07.21260122.

7. A. Sheikh, J. McMenamin, B. Taylor, C. Robertson, Public Health Scotland and the EAVE II Collaborators, SARS-CoV-2 Delta VOC in Scotland: Demographics, risk of hospital admission, and vaccine effectiveness. *Lancet* **397**, 2461–2462 (2021). doi:10.1016/S0140-6736(21)01358-1 Medline

8. D. N. Fisman, A. R. Tuite, Progressive Increase in Virulence of Novel SARS-CoV-2 Variants in Ontario, Canada. *medRxiv*, 2021.2007.2005.21260050 (2021).

9. D. Planas, D. Veyer, A. Baidaliuk, I. Staropoli, F. Guivel-Benhassine, M. M. Rajah, C. Planchais, F. Porrot, N. Robillard, J. Puech, M. Prot, F. Gallais, P. Gantner, A. Velay, J. Le Guen, N. Kassis-Chikhani, D. Edriss, L. Belec, A. Seve, L. Courtellemont, H. Péré, L. Hocqueloux, S. Fafi-Kremer, T. Prazuck, H. Mouquet, T. Bruel, E. Simon-Lorière, F. A. Rey, O. Schwartz, Reduced sensitivity of SARS-CoV-2 variant Delta to antibody neutralization. *Nature* **596**, 276–280 (2021). doi:10.1038/s41586-021-03777-9 Medline

10. V. V. Edara, B. A. Pinsky, M. S. Suthar, L. Lai, M. E. Davis-Gardner, K. Floyd, M. W. Flowers, J. Wrammert, L. Hussaini, C. R. Ciric, S. Bechnak, K. Stephens, B. S. Graham, E. Bayat Mokhtari, P. Mudvari, E. Boritz, A. Creanga, A. Pegu, A. Derrien-Colemyn, A. R. Henry, M. Gagne, D. C. Douek, M. K. Sahoo, M. Sibai, D. Solis, R. J. Webby, T. Jeevan, T. P. Fabrizio, Infection and Vaccine-Induced Neutralizing-Antibody Responses to the SARS-CoV-2 B.1.617 Variants. *N. Engl. J. Med.* **385**, 664–666 (2021). doi:10.1056/NEJMc2107799 Medline

11. J. Lopez Bernal, N. Andrews, C. Gower, E. Gallagher, R. Simmons, S. Thelwall, J. Stowe, E. Tessier, N. Groves, G. Dabrera, R. Myers, C. N. J. Campbell, G. Amirthalingam, M. Edmunds, M. Zambon, K. E. Brown, S. Hopkins, M. Chand, M. Ramsay, Effectiveness of Covid-19 Vaccines against the B.1.617.2 (Delta) Variant. *N. Engl. J. Med.* **385**, 585–594 (2021). doi:10.1056/NEJMoa2108891 Medline

12. T. Tada *et al*., Comparison of Neutralizing Antibody Titers Elicited by mRNA and Adenoviral Vector Vaccine against SARS-CoV-2 Variants. *bioRxiv*, 2021.2007.2019.452771 (2021).

13. N. R. Faria, T. A. Mellan, C. Whittaker, I. M. Claro, D. D. S. Candido, S. Mishra, M. A. E. Crispim, F. C. S. Sales, I. Hawryluk, J. T. McCrone, R. J. G. Hulswit, L. A. M. Franco, M. S. Ramundo, J. G. de Jesus, P. S. Andrade, T. M. Coletti, G. M. Ferreira, C. A. M. Silva, E. R. Manuli, R. H. M. Pereira, P. S. Peixoto, M. U. G. Kraemer, N. Gaburo Jr., C. D. C. Camilo, H. Hoeltgebaum, W. M. Souza, E. C. Rocha, L. M. de Souza, M. C. de Pinho, L. J. T. Araujo, F. S. V. Malta, A. B. de Lima, J. D. P. Silva, D. A. G. Zauli, A. C. S. Ferreira, R. P. Schnekenberg, D. J. Laydon, P. G. T. Walker, H. M. Schlüter, A. L. P. Dos Santos, M. S. Vidal, V. S. Del Caro, R. M. F. Filho, H. M. Dos Santos, R. S. Aguiar, J. L. Proença-Modena, B. Nelson, J. A. Hay, M. Monod, X. Miscouridou, H. Coupland, R. Sonabend, M. Vollmer, A. Gandy, C. A. Prete Jr., V. H. Nascimento, M. A. Suchard, T. A. Bowden, S. L. K. Pond, C.-H. Wu, O. Ratmann, N. M. Ferguson, C. Dye, N. J. Loman, P. Lemey, A. Rambaut, N. A. Fraiji, M. D. P. S. S. Carvalho, O. G. Pybus, S. Flaxman, S. Bhatt, E. C. Sabino, Genomics and epidemiology of the P.1 SARS-CoV-2 lineage in Manaus, Brazil. *Science* **372**, 815–821 (2021). doi:10.1126/science.abh2644 Medline

14. M. Hoffmann, P. Arora, R. Groß, A. Seidel, B. F. Hörnich, A. S. Hahn, N. Krüger, L. Graichen, H. Hofmann-Winkler, A. Kempf, M. S. Winkler, S. Schulz, H.-M. Jäck, B. Jahrsdörfer, H. Schrezenmeier, M. Müller, A. Kleger, J. Münch, S. Pöhlmann, SARS-CoV-2 variants B.1.351 and P.1 escape from neutralizing antibodies. *Cell* **184**, 2384–2393.e12 (2021). doi:10.1016/j.cell.2021.03.036 Medline

15. P. D. Yadav *et al*., SARS CoV-2 variant B.1.617.1 is highly pathogenic in hamsters than B.1 variant. *bioRxiv*, 2021.2005.2005.442760 (2021).

16. V.-V. Edara *et al*., Infection and vaccine-induced neutralizing antibody responses to the SARS-CoV-2 B.1.617.1 variant. *bioRxiv*, 2021.2005.2009.443299 (2021).

17. B. J. Bosch, R. van der Zee, C. A. de Haan, P. J. Rottier, The coronavirus spike protein is a class I virus fusion protein: Structural and functional characterization of the fusion core complex. *J. Virol.* **77**, 8801–8811 (2003). doi:10.1128/JVI.77.16.8801-8811.2003 Medline

18. M. Hoffmann, H. Kleine-Weber, S. Schroeder, N. Krüger, T. Herrler, S. Erichsen, T. S. Schiergens, G. Herrler, N.-H. Wu, A. Nitsche, M. A. Müller, C. Drosten, S. Pöhlmann, SARS-CoV-2 Cell Entry Depends on ACE2 and TMPRSS2 and Is Blocked by a Clinically Proven Protease Inhibitor. *Cell* **181**, 271–280.e8 (2020). doi:10.1016/j.cell.2020.02.052 Medline

19. J. K. Millet, G. R. Whittaker, Host cell entry of Middle East respiratory syndrome coronavirus after two-step, furin-mediated activation of the spike protein. *Proc. Natl. Acad. Sci. U.S.A.* **111**, 15214–15219 (2014). doi:10.1073/pnas.1407087111 Medline

20. M. A. Tortorici, D. Veesler, Structural insights into coronavirus entry. *Adv. Virus Res.* **105**, 93–116 (2019). doi:10.1016/bs.aivir.2019.08.002 Medline

21. D. Wrapp, N. Wang, K. S. Corbett, J. A. Goldsmith, C.-L. Hsieh, O. Abiona, B. S. Graham, J. S. McLellan, Cryo-EM structure of the 2019-nCoV spike in the prefusion conformation. *Science* **367**, 1260–1263 (2020). doi:10.1126/science.abb2507 Medline

22. J. Shang, Y. Wan, C. Luo, G. Ye, Q. Geng, A. Auerbach, F. Li, Cell entry mechanisms of SARS-CoV-2. *Proc. Natl. Acad. Sci. U.S.A.* **117**, 11727–11734

(2020). doi:10.1073/pnas.2003138117 Medline

23. Y. Huang, C. Yang, X. F. Xu, W. Xu, S. W. Liu, Structural and functional properties of SARS-CoV-2 spike protein: Potential antivirus drug development for COVID-19. *Acta Pharmacol. Sin.* **41**, 1141–1149 (2020). doi:10.1038/s41401-020-0485-4 Medline

24. C. Zhu, G. He, Q. Yin, L. Zeng, X. Ye, Y. Shi, W. Xu, Molecular biology of the SARs-CoV-2 spike protein: A review of current knowledge. *J. Med. Virol.* **93**, 5729–5741 (2021). doi:10.1002/jmv.27132 Medline

25. N. Murgolo, A. G. Therien, B. Howell, D. Klein, K. Koeplinger, L. A. Lieberman, G. C. Adam, J. Flynn, P. McKenna, G. Swaminathan, D. J. Hazuda, D. B. Olsen, SARS-CoV-2 tropism, entry, replication, and propagation: Considerations for drug discovery and development. *PLOS Pathog.* **17**, e1009225 (2021). doi:10.1371/journal.ppat.1009225 Medline

26. R. Peng, L. A. Wu, Q. Wang, J. Qi, G. F. Gao, Cell entry by SARS-CoV-2. *Trends Biochem. Sci.* **46**, 848–860 (2021). doi:10.1016/j.tibs.2021.06.001 Medline

27. B. Korber, W. M. Fischer, S. Gnanakaran, H. Yoon, J. Theiler, W. Abfalterer, N. Hengartner, E. E. Giorgi, T. Bhattacharya, B. Foley, K. M. Hastie, M. D. Parker, D. G. Partridge, C. M. Evans, T. M. Freeman, T. I. de Silva, C. McDanal, L. G. Perez, H. Tang, A. Moon-Walker, S. P. Whelan, C. C. LaBranche, E. O. Saphire, D. C. Montefiori, A. Angyal, R. L. Brown, L. Carrilero, L. R. Green, D. C. Groves, K. J. Johnson, A. J. Keeley, B. B. Lindsey, P. J. Parsons, M. Raza, S. Rowland-Jones, N. Smith, R. M. Tucker, D. Wang, M. D. Wyles; Sheffield COVID-19 Genomics Group, Tracking Changes in SARS-CoV-2 Spike: Evidence that D614G Increases Infectivity of the COVID-19 Virus. *Cell* **182**, 812–827.e19 (2020). doi:10.1016/j.cell.2020.06.043 Medline

28. Y. Cai, J. Zhang, T. Xiao, H. Peng, S. M. Sterling, R. M. Walsh Jr., S. Rawson, S. Rits-Volloch, B. Chen, Distinct conformational states of SARS-CoV-2 spike protein. *Science* **369**, 1586–1592 (2020). doi:10.1126/science.abd4251 Medline

29. J. Yu, Z. Li, X. He, M. S. Gebre, E. A. Bondzie, H. Wan, C. Jacob-Dolan, D. R. Martinez, J. P. Nkolola, R. S. Baric, D. H. Barouch, Deletion of the SARS-CoV-2 Spike Cytoplasmic Tail Increases Infectivity in Pseudovirus Neutralization Assays. *J. Virol.* **95**, JVI.00044-21 (2021). doi:10.1128/JVI.00044-21 Medline

30. T. Giroglou, J. Cinatl Jr., H. Rabenau, C. Drosten, H. Schwalbe, H. W. Doerr, D. von Laer, Retroviral vectors pseudotyped with severe acute respiratory syndrome coronavirus S protein. *J. Virol.* **78**, 9007–9015 (2004). doi:10.1128/JVI.78.17.9007-9015.2004 Medline

31. J. Zhang, Y. Cai, T. Xiao, J. Lu, H. Peng, S. M. Sterling, R. M. Walsh Jr., S. Rits-Volloch, H. Zhu, A. N. Woosley, W. Yang, P. Sliz, B. Chen, Structural impact on SARS-CoV-2 spike protein by D614G substitution. *Science* **372**, 525–530 (2021). doi:10.1126/science.abf2303 Medline

32. P. Tong, A. Gautam, I. W. Windsor, M. Travers, Y. Chen, N. Garcia, N. B. Whiteman, L. G. A. McKay, N. Storm, L. E. Malsick, A. N. Honko, F. J. N. Lelis, S. Habibi, S. Jenni, Y. Cai, L. J. Rennick, W. P. Duprex, K. R. McCarthy, C. L. Lavine, T. Zuo, J. Lin, A. Zuiani, J. Feldman, E. A. MacDonald, B. M. Hauser, A. Griffths, M. S. Seaman, A. G. Schmidt, B. Chen, D. Neuberg, G. Bajic, S. C. Harrison, D. R. Wesemann, Memory B cell repertoire for recognition of evolving SARS-CoV-2 spike. *Cell* **184**, 4969–4980.e15 (2021). doi:10.1016/j.cell.2021.07.025 Medline

33. T. Xiao, J. Lu, J. Zhang, R. I. Johnson, L. G. A. McKay, N. Storm, C. L. Lavine, H. Peng, Y. Cai, S. Rits-Volloch, S. Lu, B. D. Quinlan, M. Farzan, M. S. Seaman, A. Griffiths, B. Chen, A trimeric human angiotensin-converting enzyme 2 as an anti-SARS-CoV-2 agent. *Nat. Struct. Mol. Biol.* **28**, 202–209 (2021). doi:10.1038/s41594-020-00549-3 Medline

34. T. Sztain, S.-H. Ahn, A. T. Bogetti, L. Casalino, J. A. Goldsmith, E. Seitz, R. S. McCool, F. L. Kearns, F. Acosta-Reyes, S. Maji, G. Mashayekhi, J. A. McCammon, A. Ourmazd, J. Frank, J. S. McLellan, L. T. Chong, R. E. Amaro, A glycan gate controls opening of the SARS-CoV-2 spike protein. *Nat. Chem.* **13**, 963–968 (2021). doi:10.1038/s41557-021-00758-3 Medline

35. G. Cerutti, Y. Guo, T. Zhou, J. Gorman, M. Lee, M. Rapp, E. R. Reddem, J. Yu, F. Bahna, J. Bimela, Y. Huang, P. S. Katsamba, L. Liu, M. S. Nair, R. Rawi, A. S. Olia, P. Wang, B. Zhang, G.-Y. Chuang, D. D. Ho, Z. Sheng, P. D. Kwong, L. Shapiro, Potent SARS-CoV-2 neutralizing antibodies directed against spike N-terminal domain target a single supersite. *Cell Host Microbe* **29**, 819–833.e7 (2021). doi:10.1016/j.chom.2021.03.005 Medline

36. X. Chi, R. Yan, J. Zhang, G. Zhang, Y. Zhang, M. Hao, Z. Zhang, P. Fan, Y. Dong, Y. Yang, Z. Chen, Y. Guo, J. Zhang, Y. Li, X. Song, Y. Chen, L. Xia, L. Fu, L. Hou, J. Xu, C. Yu, J. Li, Q. Zhou, W. Chen, A neutralizing human antibody binds to the N-terminal domain of the Spike protein of SARS-CoV-2. *Science* **369**, 650–655 (2020). doi:10.1126/science.abc6952 Medline

37. M. McCallum, A. De Marco, F. A. Lempp, M. A. Tortorici, D. Pinto, A. C. Walls, M. Beltramello, A. Chen, Z. Liu, F. Zatta, S. Zepeda, J. di Iulio, J. E. Bowen, M. Montiel-Ruiz, J. Zhou, L. E. Rosen, S. Bianchi, B. Guarino, C. S. Fregni, R. Abdelnabi, S. C. Foo, P. W. Rothlauf, L.-M. Bloyet, F. Benigni, E. Cameroni, J. Neyts, A. Riva, G. Snell, A. Telenti, S. P. J. Whelan, H. W. Virgin, D. Corti, M. S. Pizzuto, D. Veesler, N-terminal domain antigenic mapping reveals a site of vulnerability for SARS-CoV-2. *Cell* **184**, 2332–2347.e16 (2021). doi:10.1016/j.cell.2021.03.028 Medline

38. D. Li, R. J. Edwards, K. Manne, D. R. Martinez, A. Schäfer, S. M. Alam, K. Wiehe, X. Lu, R. Parks, L. L. Sutherland, T. H. Oguin 3rd, C. McDanal, L. G. Perez, K. Mansouri, S. M. C. Gobeil, K. Janowska, V. Stalls, M. Kopp, F. Cai, E. Lee, A. Foulger, G. E. Hernandez, A. Sanzone, K. Tilahun, C. Jiang, L. V. Tse, K. W. Bock, M. Minai, B. M. Nagata, K. Cronin, V. Gee-Lai, M. Deyton, M. Barr, T. Von Holle, A. N. Macintyre, E. Stover, J. Feldman, B. M. Hauser, T. M. Caradonna, T. D. Scobey, W. Rountree, Y. Wang, M. A. Moody, D. W. Cain, C. T. DeMarco, T. N. Denny, C. W. Woods, E. W. Petzold, A. G. Schmidt, I.-T. Teng, T. Zhou, P. D. Kwong, J. R. Mascola, B. S. Graham, I. N. Moore, R. Seder, H. Andersen, M. G. Lewis, D. C. Montefiori, G. D. Sempowski, R. S. Baric, P. Acharya, B. F. Haynes, K. O. Saunders, In vitro and in vivo functions of SARS-CoV-2 infection-enhancing and neutralizing antibodies. *Cell* **184**, 4203–4219.e32 (2021). doi:10.1016/j.cell.2021.06.021 Medline

39. M. McCallum *et al.*, Molecular basis of immune evasion by the delta and kappa SARS-CoV-2 variants. *bioRxiv*, (2021).

40. J. Lan, J. Ge, J. Yu, S. Shan, H. Zhou, S. Fan, Q. Zhang, X. Shi, Q. Wang, L. Zhang, X. Wang, Structure of the SARS-CoV-2 spike receptor-binding domain bound to the ACE2 receptor. *Nature* **581**, 215–220 (2020). doi:10.1038/s41586-020-2180-5 Medline

41. J. Shang, G. Ye, K. Shi, Y. Wan, C. Luo, H. Aihara, Q. Geng, A. Auerbach, F. Li, Structural basis of receptor recognition by SARS-CoV-2. *Nature* **581**, 221–224 (2020). doi:10.1038/s41586-020-2179-y Medline

42. A. C. Walls, Y.-J. Park, M. A. Tortorici, A. Wall, A. T. McGuire, D. Veesler, Structure, Function, and Antigenicity of the SARS-CoV-2 Spike Glycoprotein. *Cell* **183**, 1735 (2020). doi:10.1016/j.cell.2020.11.032 Medline

43. S. M. Gobeil, K. Janowska, S. McDowell, K. Mansouri, R. Parks, V. Stalls, M. F. Kopp, K. Manne, D. Li, K. Wiehe, K. O. Saunders, R. J. Edwards, B. Korber, B. F. Haynes, R. Henderson, P. Acharya, Effect of natural mutations of SARS-CoV-2 on spike structure, conformation, and antigenicity. *Science* **373**, eabi6226 (2021). doi:10.1126/science.abi6226 Medline

44. T. N. Starr, A. J. Greaney, S. K. Hilton, D. Ellis, K. H. D. Crawford, A. S. Dingens, M. J. Navarro, J. E. Bowen, M. A. Tortorici, A. C. Walls, N. P. King, D. Veesler, J. D. Bloom, Deep Mutational Scanning of SARS-CoV-2 Receptor Binding Domain Reveals Constraints on Folding and ACE2 Binding. *Cell* **182**, 1295–1310.e20 (2020). doi:10.1016/j.cell.2020.08.012 Medline

45. M. Yuan, D. Huang, C. D. Lee, N. C. Wu, A. M. Jackson, X. Zhu, H. Liu, L. Peng,

M. J. van Gils, R. W. Sanders, D. R. Burton, S. M. Reincke, H. Prüss, J. Kreye, D. Nemazee, A. B. Ward, I. A. Wilson, Structural and functional ramifications of antigenic drift in recent SARS-CoV-2 variants. *Science* **373**, 818–823 (2021). doi:10.1126/science.abh1139 Medline

46. T. J. Yang, P.-Y. Yu, Y.-C. Chang, K.-H. Liang, H.-C. Tso, M.-R. Ho, W.-Y. Chen, H.-T. Lin, H.-C. Wu, S. D. Hsu, Effect of SARS-CoV-2 B.1.1.7 mutations on spike protein structure and function. *Nat. Struct. Mol. Biol.* **28**, 731–739 (2021). doi:10.1038/s41594-021-00652-z Medline

47. M. Ferrari, L. Mekkaoui, F. T. Ilca, Z. Akbar, R. Bughda, K. Lamb, K. Ward, F. Parekh, R. Karattil, C. Allen, P. Wu, V. Baldan, G. Mattiuzzo, E. M. Bentley, Y. Takeuchi, J. Sillibourne, P. Datta, A. Kinna, M. Pule, S. C. Onuoha, Characterization of a Novel ACE2-Based Therapeutic with Enhanced Rather than Reduced Activity against SARS-CoV-2 Variants. *J. Virol.* **95**, e0068521 (2021). doi:10.1128/JVI.00685-21 Medline

48. P. Wang, M. S. Nair, L. Liu, S. Iketani, Y. Luo, Y. Guo, M. Wang, J. Yu, B. Zhang, P. D. Kwong, B. S. Graham, J. R. Mascola, J. Y. Chang, M. T. Yin, M. Sobieszczyk, C. A. Kyratsous, L. Shapiro, Z. Sheng, Y. Huang, D. D. Ho, Antibody resistance of SARS-CoV-2 variants B.1.351 and B.1.1.7. *Nature* **593**, 130–135 (2021). doi:10.1038/s41586-021-03398-2 Medline

49. E. Volz, S. Mishra, M. Chand, J. C. Barrett, R. Johnson, L. Geidelberg, W. R. Hinsley, D. J. Laydon, G. Dabrera, Á. O'Toole, R. Amato, M. Ragonnet-Cronin, I. Harrison, B. Jackson, C. V. Ariani, O. Boyd, N. J. Loman, J. T. McCrone, S. Gonçalves, D. Jorgensen, R. Myers, V. Hill, D. K. Jackson, K. Gaythorpe, N. Groves, J. Sillitoe, D. P. Kwiatkowski, S. Flaxman, O. Ratmann, S. Bhatt, S. Hopkins, A. Gandy, A. Rambaut, N. M. Ferguson; COVID-19 Genomics UK (COG-UK) consortium, Assessing transmissibility of SARS-CoV-2 lineage B.1.1.7 in England. *Nature* **593**, 266–269 (2021). doi:10.1038/s41586-021-03470-x Medline

50. M. U. G. Kraemer, V. Hill, C. Ruis, S. Dellicour, S. Bajaj, J. T. McCrone, G. Baele, K. V. Parag, A. L. Battle, B. Gutierrez, B. Jackson, R. Colquhoun, Á. O'Toole, B. Klein, A. Vespignani, E. Volz, N. R. Faria, D. M. Aanensen, N. J. Loman, L. du Plessis, S. Cauchemez, A. Rambaut, S. V. Scarpino, O. G. Pybus; COVID-19 Genomics UK (COG-UK) Consortium, Spatiotemporal invasion dynamics of SARS-CoV-2 lineage B.1.1.7 emergence. *Science* **373**, 889–895 (2021). doi:10.1126/science.abj0113 Medline

51. N. G. Davies, S. Abbott, R. C. Barnard, C. I. Jarvis, A. J. Kucharski, J. D. Munday, C. A. B. Pearson, T. W. Russell, D. C. Tully, A. D. Washburne, T. Wenseleers, A. Gimma, W. Waites, K. L. M. Wong, K. van Zandvoort, J. D. Silverman, K. Diaz-Ordaz, R. Keogh, R. M. Eggo, S. Funk, M. Jit, K. E. Atkins, W. J. Edmunds; CMMID COVID-19 Working Group, COVID-19 Genomics UK (COG-UK) Consortium, Estimated transmissibility and impact of SARS-CoV-2 lineage B.1.1.7 in England. *Science* **372**, eabg3055 (2021). doi:10.1126/science.abg3055 Medline

52. S. Kim *et al*., Differential Interactions Between Human ACE2 and Spike RBD of SARS-CoV-2 Variants of Concern. *bioRxiv*, 2021.2007.2023.453598 (2021).

53. A. Saito *et al*., SARS-CoV-2 spike P681R mutation enhances and accelerates viral fusion. *bioRxiv*, 2021.2006.2017.448820 (2021).

54. Y. Liu *et al*., Delta spike P681R mutation enhances SARS-CoV-2 fitness over Alpha variant. *bioRxiv*, 2021.2008.2012.456173 (2021).

55. L. Liu, P. Wang, M. S. Nair, J. Yu, M. Rapp, Q. Wang, Y. Luo, J. F.-W. Chan, V. Sahi, A. Figueroa, X. V. Guo, G. Cerutti, J. Bimela, J. Gorman, T. Zhou, Z. Chen, K.-Y. Yuen, P. D. Kwong, J. G. Sodroski, M. T. Yin, Z. Sheng, Y. Huang, L. Shapiro, D. D. Ho, Potent neutralizing antibodies against multiple epitopes on SARS-CoV-2 spike. *Nature* **584**, 450–456 (2020). doi:10.1038/s41586-020-2571-7 Medline

56. C. O. Barnes, A. P. West Jr., K. E. Huey-Tubman, M. A. G. Hoffmann, N. G. Sharaf, P. R. Hoffman, N. Koranda, H. B. Gristick, C. Gaebler, F. Muecksch, J. C. C. Lorenzi, S. Finkin, T. Hägglöf, A. Hurley, K. G. Millard, Y. Weisblum, F. Schmidt, T. Hatziioannou, P. D. Bieniasz, M. Caskey, D. F. Robbiani, M. C. Nussenzweig, P. J. Bjorkman, Structures of Human Antibodies Bound to SARS-CoV-2 Spike Reveal Common Epitopes and Recurrent Features of Antibodies. *Cell* **182**, 828–842.e16 (2020). doi:10.1016/j.cell.2020.06.025 Medline

57. J. Chen, J. M. Kovacs, H. Peng, S. Rits-Volloch, J. Lu, D. Park, E. Zablowsky, M. S. Seaman, B. Chen, HIV-1 ENVELOPE. Effect of the cytoplasmic domain on antigenic characteristics of HIV-1 envelope glycoprotein. *Science* **349**, 191–195 (2015). doi:10.1126/science.aaa9804 Medline

58. C. Z. Chen, M. Xu, M. Pradhan, K. Gorshkov, J. D. Petersen, M. R. Straus, W. Zhu, P. Shinn, H. Guo, M. Shen, C. Klumpp-Thomas, S. G. Michael, J. Zimmerberg, W. Zheng, G. R. Whittaker, Identifying SARS-CoV-2 Entry Inhibitors through Drug Repurposing Screens of SARS-S and MERS-S Pseudotyped Particles. *ACS Pharmacol. Transl. Sci.* **3**, 1165–1175 (2020). doi:10.1021/acsptsci.0c00112 Medline

59. J. K. Millet, G. R. Whittaker, Murine Leukemia Virus (MLV)-based Coronavirus Spike-pseudotyped Particle Production and Infection. *Biol. Protoc.* **6**, (2016). doi:10.21769/BioProtoc.2035

60. D. N. Mastronarde, Automated electron microscope tomography using robust prediction of specimen movements. *J. Struct. Biol.* **152**, 36–51 (2005). doi:10.1016/j.jsb.2005.07.007 Medline

61. S. Q. Zheng, E. Palovcak, J.-P. Armache, K. A. Verba, Y. Cheng, D. A. Agard, MotionCor2: Anisotropic correction of beam-induced motion for improved cryo-electron microscopy. *Nat. Methods* **14**, 331–332 (2017). doi:10.1038/nmeth.4193 Medline

62. K. Zhang, Gctf: Real-time CTF determination and correction. *J. Struct. Biol.* **193**, 1–12 (2016). doi:10.1016/j.jsb.2015.11.003 Medline

63. T. Wagner, F. Merino, M. Stabrin, T. Moriya, C. Antoni, A. Apelbaum, P. Hagel, O. Sitsel, T. Raisch, D. Prumbaum, D. Quentin, D. Roderer, S. Tacke, B. Siebolds, E. Schubert, T. R. Shaikh, P. Lill, C. Gatsogiannis, S. Raunser, SPHIRE-crYOLO is a fast and accurate fully automated particle picker for cryo-EM. *Commun. Biol.* **2**, 218 (2019). doi:10.1038/s42003-019-0437-z Medline

64. S. H. Scheres, RELION: Implementation of a Bayesian approach to cryo-EM structure determination. *J. Struct. Biol.* **180**, 519–530 (2012). doi:10.1016/j.jsb.2012.09.006 Medline

65. A. Kucukelbir, F. J. Sigworth, H. D. Tagare, Quantifying the local resolution of cryo-EM density maps. *Nat. Methods* **11**, 63–65 (2014). doi:10.1038/nmeth.2727 Medline

66. P. Emsley, B. Lohkamp, W. G. Scott, K. Cowtan, Features and development of Coot. *Acta Crystallogr. D Biol. Crystallogr.* **66**, 486–501 (2010). doi:10.1107/S0907444910007493 Medline

67. P. D. Adams, P. V. Afonine, G. Bunkóczi, V. B. Chen, I. W. Davis, N. Echols, J. J. Headd, L.-W. Hung, G. J. Kapral, R. W. Grosse-Kunstleve, A. J. McCoy, N. W. Moriarty, R. Oeffner, R. J. Read, D. C. Richardson, J. S. Richardson, T. C. Terwilliger, P. H. Zwart, PHENIX: A comprehensive Python-based system for macromolecular structure solution. *Acta Crystallogr. D Biol. Crystallogr.* **66**, 213–221 (2010). doi:10.1107/S0907444909052925 Medline

68. T. I. Croll, ISOLDE: A physically realistic environment for model building into low-resolution electron-density maps. *Acta Crystallogr. D Struct. Biol.* **74**, 519–530 (2018). doi:10.1107/S2059798318002425 Medline

69. A. Morin, B. Eisenbraun, J. Key, P. C. Sanschagrin, M. A. Timony, M. Ottaviano, P. Sliz, Collaboration gets the most out of software. *eLife* **2**, e01456 (2013). doi:10.7554/eLife.01456 Medline

ACKNOWLEDGMENTS

We thank the SBGrid team for technical assistance, K. Arnett for support and advice on the BLI experiments, and S. Harrison and A. Carfi for critical reading of the manuscript. EM data were collected at the Harvard Cryo-EM Center for Structural Biology of Harvard Medical School. We acknowledge support for COVID-19 related structural biology research at Harvard from the Nancy Lurie Marks Family Foundation and the Massachusetts Consortium on Pathogen Readiness (MassCPR). **Funding:** This work was supported by Fast grants by Emergent Ventures (to B.C. and D.R.W.), COVID-19 Award by Massachusetts Consortium on Pathogen Readiness (MassCPR; to B.C. and D.R.W.), and NIH grants AI147884 (to B.C.), AI141002 (to B.C.), AI127193 (to B.C. and James Chou), and AI39538 and AI165072 (to D.R.W). **Author Contributions:** B.C., J.Z., T.X., and Y.C. conceived the project. Y.C. expressed and purified the full-length S proteins with help from H.P., and carried out negative stain EM. T.X. performed BLI and cell-cell fusion experiments. J.Z. prepared cryo grids and performed EM data collection with contributions from M.L.M. and R.M.W., and processed the cryo-EM data, built and refined the atomic models. J.L. created all the expression constructs and performed the neutralization assays using the MLV-based pseudoviruses. C.L.L. and M.S.S performed the neutralization assays using the HIV-based pseudoviruses. H.Z., K.A., and W.Y. performed the flow cytometry experiments. P.T., A.G., and, D.R.W. produced anti-S monoclonal antibodies. S.R.V. contributed to cell culture and protein production. All authors analyzed the data. B.C., J.Z., T.X., and Y.C. wrote the manuscript with input from all other authors. **Competing Interests:** W.Y. serves on the scientific advisory boards of Hummingbird Bioscience and GO Therapeutics and is currently an employee of GV20 Therapeutics LLC. All other authors declare no competing interests. **Data and materials availability:** The atomic structure coordinates are deposited in the RCSB Protein Data Bank (PDB) under the accession numbers 7SBK, 7SBL, 7SBO, 7SBP, 7SBQ, 7SBR, 7SBS, and 7SBT; and the electron microscopy maps have been deposited in the Electron Microscopy Data Bank (EMDB) under the accession numbers EMD-24981, EMD-24982, EMD-24983, EMD-24984, EMD-24985, EMD-24986, EMD-24987, and EMD-24988. All materials generated during the current study are available from the corresponding author under an MTA with Boston Children's Hospital. This work is licensed under a Creative Commons Attribution 4.0 International (CC BY 4.0) license, which permits unrestricted use, distribution, and reproduction in any medium, provided the original work is properly cited. To view a copy of this license, visit https://creativecommons.org/licenses/by/4.0/. This license does not apply to figures/photos/artwork or other content included in the article that is credited to a third party; obtain authorization from the rights holder before using such material.

SUPPLEMENTARY MATERIALS

science.org/doi/10.1126/science.abl9463
Materials and Methods
Supplementary Text
Figs. S1 to S8
Tables S1 to S3
References (*57*–*69*)
MDAR Reproducibility Checklist

15 August 2021; accepted 22 October 2021
Published online 26 October 2021
10.1126/science.abl9463

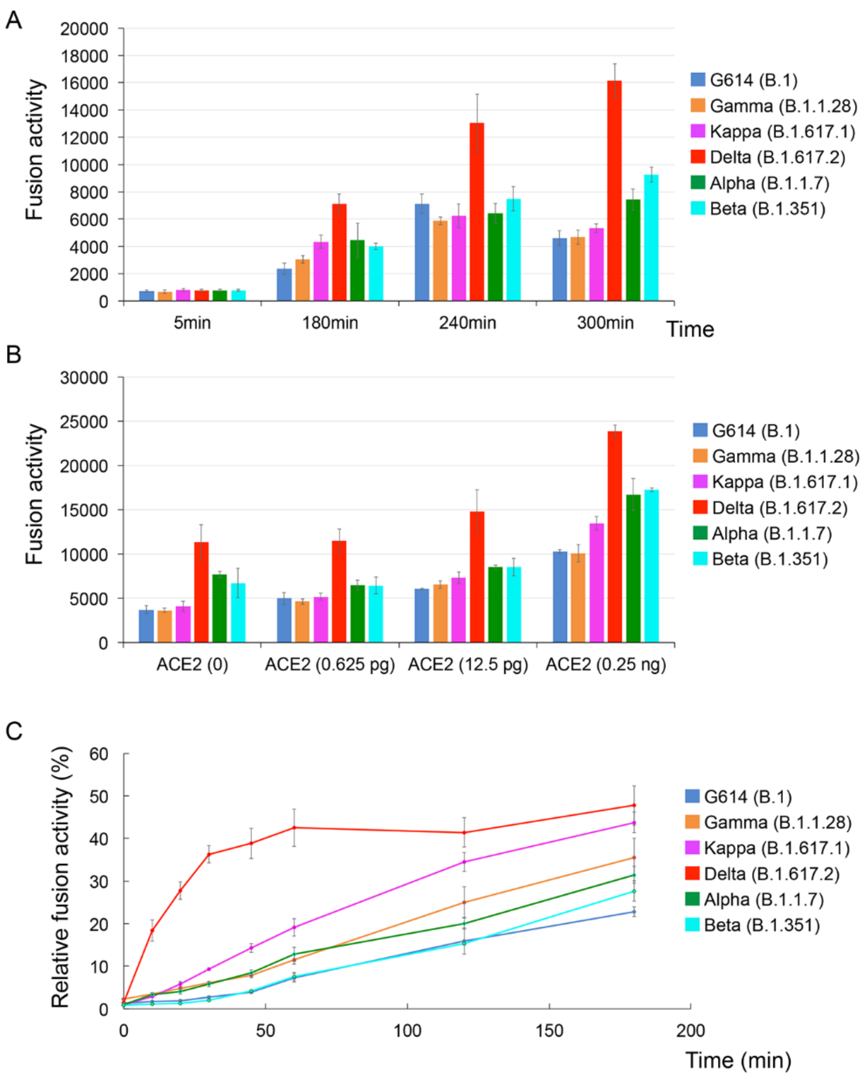

Fig. 1. More efficient membrane fusion by the Delta variant than other variants. (**A**) Time course of cell-cell fusion mediated by various full-length S proteins, as indicated, with HEK293 cells with no exogenous ACE2. (**B**) Cell-cell fusion mediated by various full-length S proteins with HEK293 cells transfected with low levels (0 to 0.25 ng) of ACE2 expression constructs. (**C**) Time course of infection HEK293-ACE2 cells by MLV-based, pseudotyped viruses using various SARS-CoV-2 variant S constructs containing a CT deletion in a single cycle. Infection was initiated by mixing viruses and target cells, and viruses were washed out at each time point as indicated. The full time course and concentration series are shown in fig. S3. The experiments were repeated at least three times with independent samples giving similar results.

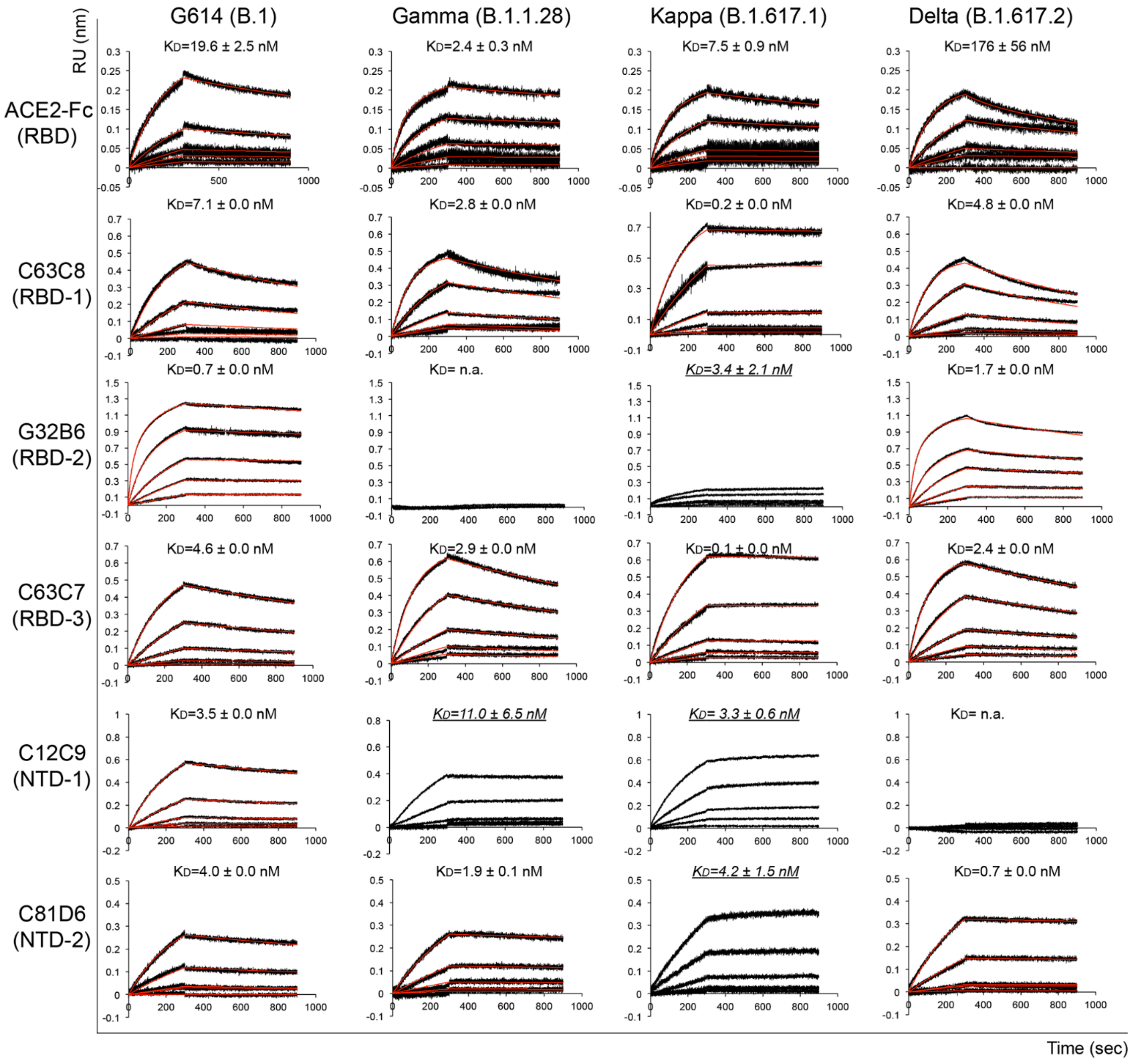

Fig. 2. Antigenic properties of the purified full-length SARS-CoV-2 S proteins. Bio-layer interferometry (BLI) analysis of the association of prefusion S trimers derived from the G614 "parent" strain (B.1) and the Gamma (B.1.1.28), Kappa (B.1.617.1) and Delta (B.1.617.2) variants with soluble ACE2 constructs and with a panel of antibodies representing five epitopic regions on the RBD and NTD (see fig. S4A) (*32*). For ACE2 binding, purified S proteins were immobilized to AR2G biosensors and dipped into the wells containing ACE2 at various concentrations. For antibody binding, various antibodies were immobilized to AHC biosensors and dipped into the wells containing each purified S protein at different concentrations. Binding kinetics were evaluated using a 1:1 Langmuir model except for dimeric ACE2 and antibody G32B6 targeting the RBD-2, which were analyzed by a bivalent binding model. All K_D values for multivalent interactions with antibody IgG or dimeric ACE2 and trimeric S protein are the apparent affinities with avidity effects. The sensorgrams are in black and the fits in red. Binding constants highlighted by underlines were estimated by steady-state analysis as described in the Methods. RU, response unit. Binding constants are also summarized here and in table S1. All experiments were repeated at least twice with essentially identical results.

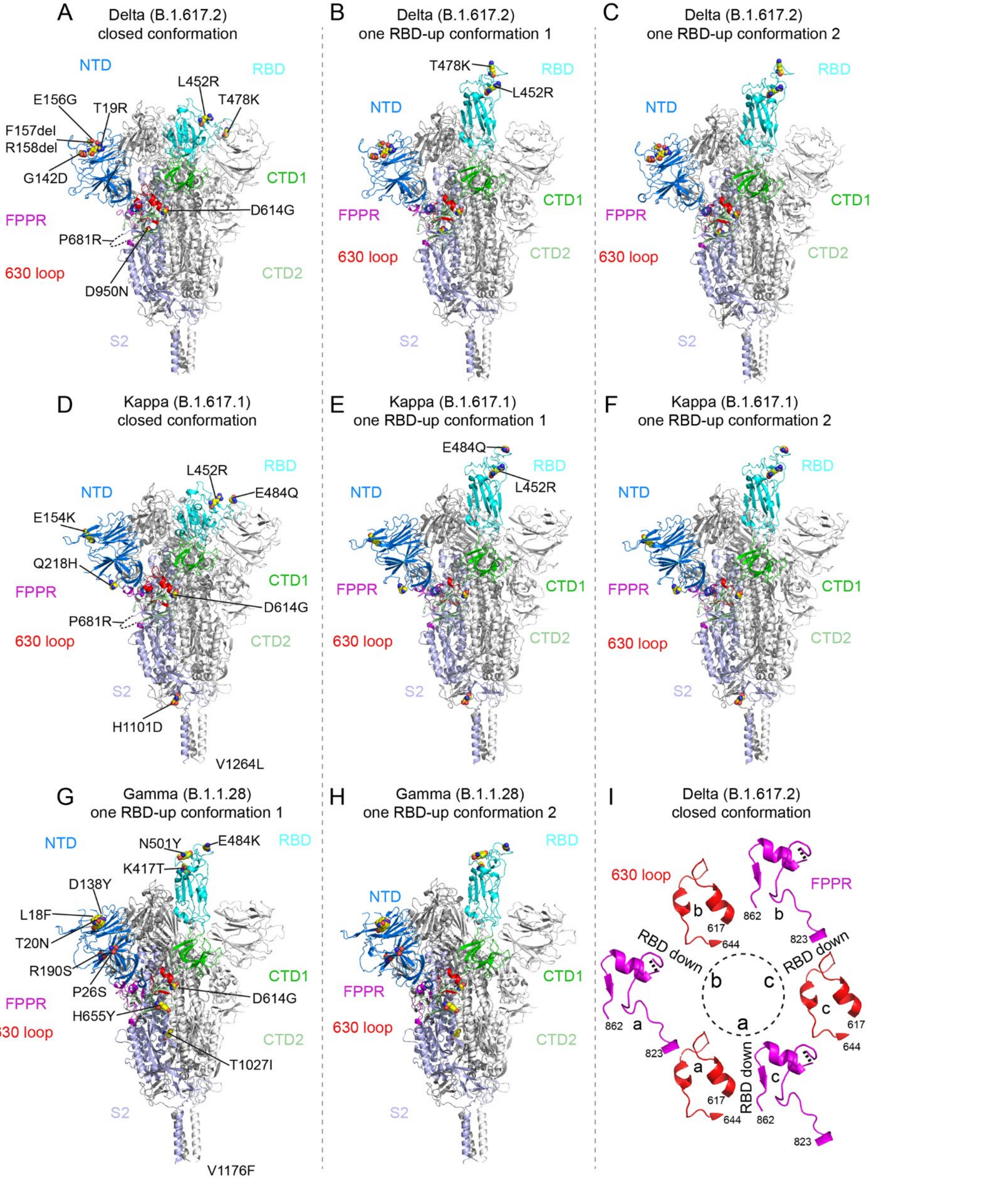
A
Delta (B.1.617.2)
closed conformation
NTD
RBD
L452R
T478K
E156G
T19R
F157del
R158del
G142D
CTD1
FPPR
D614G
P681R
630 loop
CTD2
D950N
S2
B
Delta (B.1.617.2)
one RBD-up conformation 1
T478K
L452R
C
Delta (B.1.617.2)
one RBD-up conformation 2
D
Kappa (B.1.617.1)
closed conformation
E484Q
E154K
Q218H
H1101D
V1264L
E
Kappa (B.1.617.1)
one RBD-up conformation 1
F
Kappa (B.1.617.1)
one RBD-up conformation 2
G
Gamma (B.1.1.28)
one RBD-up conformation 1
N501Y
E484K
K417T
D138Y
L18F
T20N
R190S
P26S
H655Y
T1027I
V1176F
H
Gamma (B.1.1.28)
one RBD-up conformation 2
I
Delta (B.1.617.2)
closed conformation
RBD down
617
644
862
823
a
b
c

Fig. 3. Figure 3. Cryo-EM structures of the full-length SARS-CoV-2 S proteins from the Delta, Kappa and Gamma variants. (**A** to **C**) The structures of the closed prefusion conformation and two one-RBD-up conformations of the Delta S trimer are shown in ribbon diagram with one protomer colored as NTD in blue, RBD in cyan, CTD1 in green, CTD2 in light green, S2 in light blue, the 630 loop in red and the FPPR in magenta. (**D** to **F**) The structures of the closed prefusion conformation and two one-RBD-up conformation of the Kappa S trimer are shown in ribbon diagram with the same color scheme as in (A). (**G**) and (**H**) The structures of the two one-RBD-up conformations of the Gamma S trimer are shown in ribbon diagram with the same color scheme as in (A). All mutations in the three variants, as compared with the original virus (D614), are highlighted in sphere model. (**I**) Structures, in the Delta closed trimer, of segments (residues 617-644) containing the 630 loop (red) and segments (residues 823-862) containing the FPPR (magenta) from each of the three protomers (A), (B) and (C). The position of each RBD is indicated. Dashed lines indicate gaps in the chain trace (disordered loops).

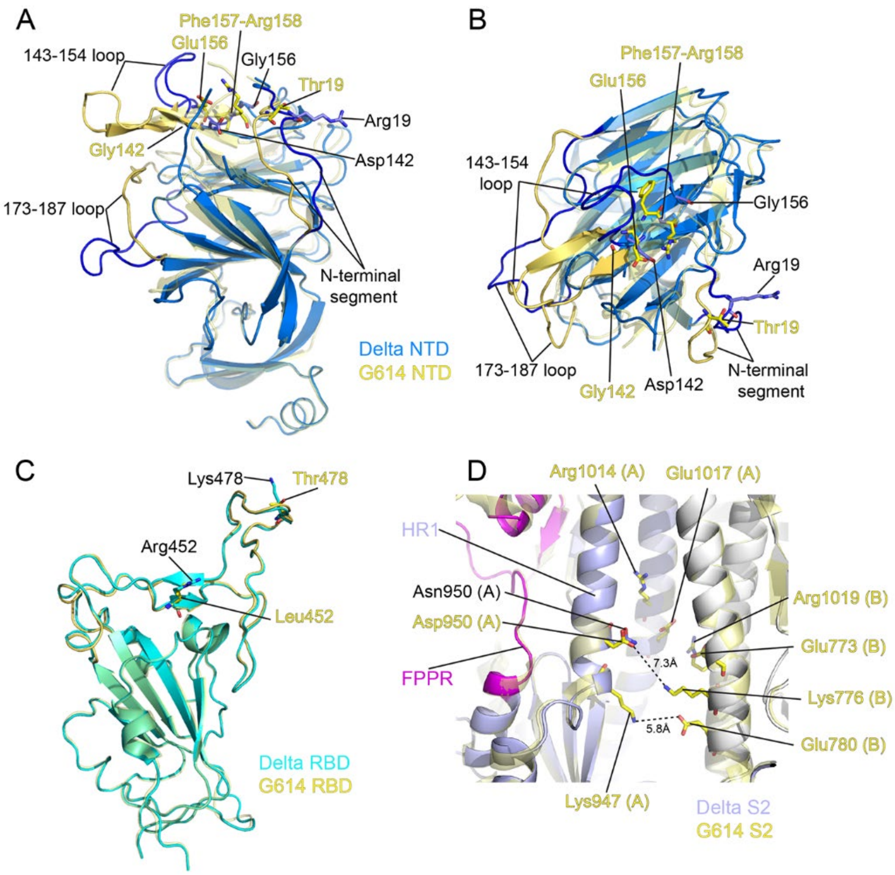

Fig. 4. Structural impact of the mutations in the Delta S. (**A**) Superposition of the NTD structure of the Delta S trimer in blue with the NTD of the G614 S trimer (PDB ID: 7KRQ) in yellow. Locations of mutations T19R, G142D, E156G, and deletion of F157 and R158 are indicated and these residues are shown in stick model. The N-terminal segment, 143-154, and 173-187 loops are rearranged between the two structures and highlighted in darker colors. (**B**) Top view of the panel (A). (**C**) Superposition of the RBD structure of the Delta S trimer in cyan with the RBD of the G614 S trimer in yellow. Locations of mutations L452R and T478K are indicated and these residues are shown in the stick model. (**D**) A close-up view of superposition of the Delta S2 in light blue with the S2 of the G614 S trimer in yellow near residue 950. Locations of the D950N mutation and charged residues in the vicinity including Lys947, Arg1014, and Glu1017 from the protomer A and Glu773, Lys776, Glu780, and Arg1019 from the protomer B are indicated. All these residues are shown in the stick model.

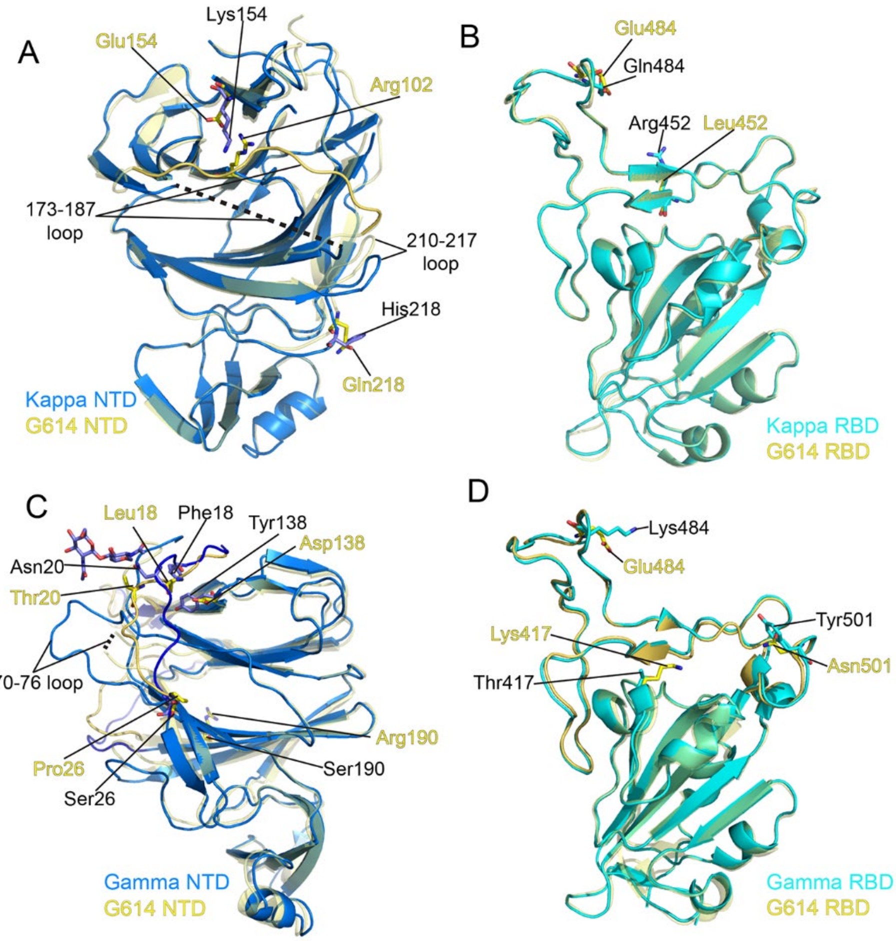

Fig. 5. Structural impact of the mutations in the Kappa and Gamma S proteins. (**A**) Superposition of the NTD structure of the Kappa S trimer in blue with the NTD of the G614 S trimer in yellow. Locations of mutations E154K and Q218H, as well as Arg102 that forms a salt bridge with Glu154 in the G614 structure are indicated and these residues are shown in the stick model. The 173 to 187 loop in the G614 trimer is highlighted in a darker color; it becomes disordered in the Kappa trimer. (**B**) Superposition of the RBD structure of the Kappa S trimer in cyan with the RBD of the G614 S trimer in yellow. Locations of mutations L452R and E484Q are indicated and these residues are shown in the stick model. (**C**) A view of superposition of the NTD structures of the Gamma (blue) and G614 (yellow; PDB ID: 7KRR) S trimers in the one-RBD-up conformation. Locations of mutations L18F, T20N, P26S, D138Y and R190S, as well as N-linked glycan attached to Asn20 in the Gamma structure are indicated and these residues are shown in stick model. (**D**) Superposition of the RBD structure of the Gamma S trimer in cyan with the RBD of the G614 S trimer in yellow. Locations of mutations K417T, E484K and N501Y are indicated and these residues are shown in the stick model.
