# Supplementary material for: Membrane fusion and immune evasion by the spike protein of SARS-CoV-2 Delta variant
Source: Science. 2021 Oct 26;374(6573):1353–60. doi: 10.1126/science.abl9463 (PMC10763652; doi:10.1126/science.abl9463)
Supplement: Supplementary file 2 — Materials and Methods Figs. S1 to S18 Tables S1 to S3 References (57–69) [file science.abl9463_sm.pdf]

Science AAAS

Supplementary Materials for

Membrane fusion and immune evasion by the spike protein of SARS-CoV-2 Delta variant

Jun Zhang *et al.*

Corresponding author: Bing Chen, bchen@crystal.harvard.edu

Science **374**, 1353 (2021)
DOI: 10.1126/science.abl9463

The PDF file includes:

Materials and Methods
Figs. S1 to S18
Tables S1 to S3
References

Other Supplementary Material for this manuscript includes the following:

MDAR Reproducibility Checklist

Materials and Methods

Expression constructs

Genes of the full-length spike (S) proteins with the wildtype sequences from Gamma (hCoV-19/Brazil/AM-992/2020; GISAID accession ID: EPI_ISL_833172), Kappa (hCoV-19/India/MH-NEERI-NGP-40449/2021; GISAID accession ID: EPI_ISL_1547802) and Delta (hCoV-19/India/GJ-GBRC619/2021; GISAID accession ID: EPI_ISL_2020954) were codon optimized and synthesized by Twist Bioscience (South San Francisco, CA) or GENEWIZ (South Plainfield, NJ). The S genes were fused with a C-terminal twin Strep tag (SGGGSAWSHPQFEKGGGSGGGSGGSSAWSHPQFEK) and cloned into a mammalian cell expression vector pCMV-IRES-puro (Codex BioSolutions, Inc, Gaithersburg, MD).

Expression and purification of recombinant proteins

Expression and purification of the full-length S proteins with the wildtype sequences were carried out as previously described (*28*). Briefly, expi293F cells (ThermoFisher Scientific, Waltham, MA) were transiently transfected with the S protein expression constructs. To purify the S protein, the transfected cells were lysed in a solution containing Buffer A (100 mM Tris-HCl, pH 8.0, 150 mM NaCl, 1 mM EDTA) and 1% (w/v) n-dodecyl-β-D-maltopyranoside (DDM) (Anatrace, Inc. Maumee, OH), EDTA-free complete protease inhibitor cocktail (Roche, Basel, Switzerland), and incubated at 4°C for one hour. After a clarifying spin, the supernatant was loaded on a strep-tactin column equilibrated with the lysis buffer. The column was then washed with 50 column volumes of Buffer A and 0.3% DDM, followed by additional washes with 50 column volumes of Buffer A and 0.1% DDM, and with 50 column volumes of Buffer A and 0.02% DDM. The S protein was eluted by Buffer A containing 0.02% DDM and 5 mM d-Desthiobiotin. The protein was further purified by gel filtration chromatography on a Superose 6 10/300 column (GE Healthcare, Chicago, IL) in a buffer containing 25 mM Tris-HCl, pH 7.5, 150 mM NaCl, 0.02% DDM. All RBD proteins were purchased from Sino Biological US Inc (Wayne, PA).

The monomeric ACE2 or dimeric ACE2 proteins were produced as described (*33*). Briefly, Expi293F cells transfected with monomeric ACE2 or dimeric ACE2 expression construct and the supernatant of the cell culture was collected. The monomeric ACE2 protein was purified by affinity chromatography using Ni Sepharose excel (Cytiva Life Sciences, Marlborough, MA), followed by gel filtration chromatography. The dimeric ACE2 protein was purified by GammaBind Plus Sepharose beads (GE Healthcare), followed gel filtration chromatography on a Superdex 200 Increase 10/300 GL column. All the monoclonal antibodies were produced as described (*32*).

Western blot

Western blot was performed using an anti-SARS-COV-2 S antibody following a protocol described previously (*57*). Briefly, full-length S protein samples were prepared from cell pellets and resolved in 4-15% Mini-Protean TGX gel (Bio-Rad, Hercules, CA) and transferred onto PVDF membranes. Membranes were blocked with 5% skimmed milk in PBS for 1 hour and incubated with a SARS-CoV-2 (2019-nCoV) Spike RBD Antibody (Sino Biological Inc., Beijing, China, Cat: 40592-T62) for another hour at room temperature. Alkaline phosphatase conjugated anti-Rabbit IgG (1:5000) (Sigma-Aldrich, St. Louis, MO) was used as a secondary antibody. Proteins were visualized using one-step NBT/BCIP substrates (Promega, Madison, WI).

Negative stain EM

To prepare grids, 3 μl of freshly purified full-length S protein was adsorbed to a glow-discharged carbon-coated copper grid (Electron Microscopy Sciences), washed with deionized water, and stained with freshly prepared 1.5% uranyl formate. Images were recorded at room temperature at a magnification of 67,000x and a defocus value of 2.5 μm following low-dose procedures, using a Tecnai T12 electron microscope (Thermo Fisher Scientific) equipped with a Gatan UltraScan 895 4k CCD camera and operated at a voltage of 120 keV.

Cell-cell fusion assay

The cell-cell fusion assay, based on the α-complementation of E. coli β-galactosidase, was used to measure fusion activity of SARS-CoV2 S proteins, as described (*28*). Briefly, HEK293T cells

were transfected by polyethylenimine (PEI) (80 μg) with various amounts of the full-length SARS-CoV2 (Wuhan-Hu-1, G614, Alpha, Beta, Gamma, Delta or Kappa) S construct, as indicated in each specific experiment (0.025-10 μg), and the α fragment of E. coli β-galactosidase construct (10 μg), as well as the empty vector to make up the total DNA amount to 20 μg, to generate S-expressing cells. The full-length ACE2 construct, as indicated in each specific experiment (0.625 pg-10 μg) together with the ω fragment of E. coli β-galactosidase construct (10 μg), and the empty vector when needed were used to transfect HEK293T cells to create target cells. After incubation at 37°C for 24 hrs, the cells were detached using PBS buffer and resuspended in complete DMEM medium. 50 μl S-expressing cells (1.0×10^6 cells/ml) were mixed with 50 μl ACE2-expressing target cells (1.0×10^6 cells/ml) to allow cell-cell fusion to proceed at 37 °C for from 5 min to 6 hours as indicated. Cell-cell fusion activity was quantified using a chemiluminescent assay system, Gal-Screen (Applied Biosystems, Foster City, CA), following the standard protocol recommended by the manufacturer. The substrate was added to the cell mixture and allowed to react for 90 min in dark at room temperature. The luminescence signal was recorded with a Synergy Neo plate reader (Biotek, Winooski, VT).

Binding assay by bio-layer interferometry (BLI)

Binding of monomeric or dimeric ACE2 to the full-length Spike protein of each variant was measured using an Octet RED384 system (ForteBio, Fremont, CA), following the protocol described previously (*33*). Briefly, a full-length S protein was immobilized to Amine Reactive 2nd Generation (AR2G) biosensors (ForteBio, Fremont, CA) and dipped in the wells containing the ACE2 protein at various concentrations (5.56-450 nM for monomeric ACE2; 0.926-75 nM for dimeric ACE2) for association for 5 minutes, followed by a 10 min dissociation phase in a running buffer (PBS, 0.02% Tween 20, 2 mg/ml BSA). To measure binding of a full-length S protein to monoclonal antibodies, the antibody was immobilized to anti-human IgG Fc Capture (AHC) biosensor (ForteBio, Fremont, CA) following a protocol recommended by the manufacturer. The full-length S protein was diluted using a running buffer (PBS, 0.02% Tween 20, 0.02% DDM, 2 mg/ml BSA) to various concentrations (0.617-50 nM) and transferred to a 96-well plate. The sensors were dipped in the wells containing the S protein solutions for 5 min, followed with a 10 min dissociation phase in the running buffer. Control sensors with no S protein or antibody were also dipped in the ACE2 or S protein solutions and the running buffer

as references. Recorded sensorgrams with background subtracted from the references were analyzed using the software Octet Data Analysis HT Version 12.0 (ForteBio). Binding kinetics was evaluated using a 1:1 Langmuir model except for dimeric ACE2 and antibodies G32B6 and C12A2, which were analyzed by a bivalent binding model. Sensorgrams showing unrealistic off-rates were fit individually to a single exponential function shown below to obtain the steady state response R_{eq} at each concentration.

$$R = Req * (1 - e^{-k*t})$$

The K_D was obtained by fitting R_{eq} value and its corresponding concentration to the model: "one site-specific" using GraphPad Prism 8.0.2 according to H.J. Motulsky, Prism 5 Statistics Guide, 2007, GraphPad Software Inc., San Diego CA, www.graphpad.com). All K_D values for multivalent interactions with antibody IgG or dimeric ACE2 and trimeric S protein are the apparent affinities with avidity effects.

Flow cytometry

Expi293F cells (ThermoFisher Scientific) were grown in Expi293 expression medium (ThermoFisher Scientific). Cell surface display DNA constructs for the SARS-CoV-2 spike variants together with a plasmid expressing blue fluorescent protein (BFP) were transiently transfected into Expi293F cells using ExpiFectamine 293 reagent (ThermoFisher Scientific) per manufacturer's instruction. Two days after transfection, the cells were stained with primary antibodies or the histagged $ACE2_{615}$-foldon T27W protein (*33*) at 10 μg/ml concentration. For antibody staining, an Alexa Fluor 647 conjugated donkey anti-human IgG Fc F(ab')2 fragment (Jackson ImmunoResearch, West Grove, PA) was used as secondary antibody at 5 μg/ml concentration. For $ACE2_{615}$-foldon T27W staining, APC conjugated anti-HIS antibody (Miltenyi Biotec, Auburn, CA) was used as secondary antibody at 1:50 dilution. Cells were run through an Intellicyt iQue Screener Plus flow cytometer. Cells gated for positive BFP expression were analyzed for antibody and $ACE2_{615}$-foldon T27W binding. The flow cytometry assays were repeated three times with essentially identical results.

MLV-based pseudovirus assay

Murine Leukemia Virus (MLV) particles (plasmids of the MLV components kindly provided by Dr. Gary Whittaker at Cornell University and Drs. Catherine Chen and Wei Zheng at National

Center for Advancing Translational Sciences, National Institutes of Health), pseudotyped with various SARS-CoV-2 S protein constructs, were generated in HEK 293T cells, following a protocol described previously for SARS-CoV (*58, 59*). To enhance incorporation of S protein into the particles, the C-terminal 19 residues in the cytoplasmic tail of each S protein were deleted. To prepare for infection, 7.5×10^3 of HEK 293 cells, stably transfected with a full-length human ACE2 expression construct, in 15 μl culture medium were plated into a 384-well white-clear plate coated with poly-D-Lysine to enhance the cell attachment. On day 2, 15 μl of MLV pseudoviruses for each variant were added into each well pre-seeded with HEK293-ACE2 cells. The plate was centrifuged at 114 xg for 5 min at 12°C. After incubation of the pseudoviruses with the cells for a time period (10 min-8 hr), as indicated in the figures, the medium was removed and the cells were washed once with 1xDPBS. 30 μl of fresh medium was added back into each well. The cells were then incubated at 37°C for additional 40 hr. Luciferase activities were measured with Firefly Luciferase Assay Kit (CB-80552-010, Codex BioSolutions Inc).

HIV-based pseudovirus assay

Neutralizing activity against SARS-CoV-2 pseudovirus was measured using a single-round infection assay in 293T/ACE2 target cells. Pseudotyped virus particles were produced in 293T/17 cells (ATCC) by co-transfection of plasmids encoding codon-optimized SARS-CoV-2 full-length S constructs, packaging plasmid pCMV DR8.2, and luciferase reporter plasmid pHR' CMV-Luc. G614 S, packaging and luciferase plasmids were kindly provided by Dr. Barney Graham (Vaccine Research Center, NIH). The 293T cell line stably overexpressing the human ACE2 cell surface receptor protein was kindly provided by Drs. Michael Farzan and Huihui Ma (The Scripps Research Institute). For neutralization assays, serial dilutions of monoclonal antibodies (mAbs) were performed in duplicate followed by addition of pseudovirus. Pooled serum samples from convalescent COVID-19 patients or pre-pandemic normal healthy serum (NHS) were used as positive and negative controls, respectively. Plates were incubated for 1 hour at 37°C followed by addition of 293/ACE2 target cells (1×10^4/well). Wells containing cells + pseudovirus (without sample) or cells alone acted as positive and negative infection controls, respectively. Assays were harvested on day 3 using Promega BrightGlo luciferase reagent and luminescence detected with a Promega GloMax luminometer. Titers are reported as the

concentration of mAb that inhibited 50% or 80% virus infection (IC_{50} and IC_{80} titers, respectively). All neutralization experiments were repeated twice with similar results.

Cryo-EM sample preparation and data collection

To prepare cryo EM grids, 3.5 µl of the freshly purified sample from the peak fraction in DDM at ~2.5 mg/ml for the Delta variant or ~2.0 mg/ml for the Gamma and Kappa variants was applied to a 1.2/1.3 Quantifoil gold grid (Quantifoil Micro Tools GmbH), which had been glow discharged with a PELCO easiGlow™ Glow Discharge Cleaning system (Ted Pella, Inc.) for 60 s at 15 mA. Grids were immediately plunge-frozen in liquid ethane using a Vitrobot Mark IV (ThermoFisher Scientific), and excess protein was blotted away by using grade 595 filter paper (Ted Pella, Inc.) with a blotting time of 4 s, a blotting force of -12 at 4°C with 100% humidity. The grids were first screened for ice thickness and particle distribution. Selected grids were used to acquire images by a Titan Krios transmission electron microscope (ThermoFisher Scientific) operated at 300 keV and equipped with a BioQuantum GIF/K3 direct electron detector. Automated data collection was carried out using SerialEM version 3.8.6 (*60*) at a nominal magnification of 105,000× and the K3 detector in counting mode (calibrated pixel size, 0.825 Å) at an exposure rate of 20.24 (for Delta), ~20.69/20.63/27.13 (for three data sets of Gamma), or ~21.12/20.10 (for two data sets of Kappa) electrons per pixel per second. Each movie add a total accumulated electron exposure of ~51.48 (Delta), ~54.72/54.56/53.4 (Gamma), or ~51.63/51.15 (Kappa) e-/Å^2, fractionated in 50 (Delta), 51/51/50 (Gamma), or 51/51 (Kappa) frames. Data sets were acquired using a defocus range of 0.8-2.2 (Delta), 0.8-2.3 (Gamma), or 0.8-2.2 (Kappa) µm.

Image processing and 3D reconstructions

Drift correction for cryo-EM images was performed using MotionCor2 (*61*), and contrast transfer function (CTF) was estimated by Gctf (*62*) using motion-corrected sums without dose-weighting. Motion corrected sums with dose-weighting were used for all other image processing. crYOLO (*63*) was used for particle picking and RELION3.0.8 (*64*) for 2D classification, 3D classification and refinement procedure. For the Delta sample, 1,830,328 particles were extracted from 20,274 images using crYOLO with a trained model, and then

subjected to 2D classification, giving 1,386,630 good particles. A low-resolution negative-stain reconstruction of the Wuhan-Hu-1 (D614) sample was low-pass filtered to 40Å resolution and used as an initial model for 3D classification with C1 symmetry. After two rounds of 3D classification, two major classes with clear structural features were combined and subjected to a third round of 3D classification in C1 symmetry. The calculation led to five major classes, one in a closed, three RBD-down conformation, and four in the one-RBD-up conformation. The class of the closed conformation was re-extracted unbinned and subjected to one round of 3D auto-refinement, giving a map at 4.2Å resolution from 125,763 particles. Additional round of signal-subtraction and 3D classification using a mask for the apex region of the S trimer were performed, leading to three distinct classes, two in the closed conformation and one in the one-RBD-up conformation. The two classes in the closed conformation were combined and subjected to another round of 3D auto-refinement, followed by CTF refinement, particle polishing, and 3D auto-refinement, producing a map at 4.2Å resolution from 102,521 particles. Second round of signal-subtraction and 3D classification focusing on the apex region were carried out, giving one major class in the closed conformation with 94,680 particles. Further round of 3D auto-refinement in C3 symmetry using a whole mask was applied to this class, followed by CTF refinement, particle polishing, and 3D auto-refinement, yielding a map at 3.1Å resolution. Four classes in the one-RBD-up conformation from the third rounds of 3D classification were combined, and particles re-extracted and unbinned for one round of 3D auto-refinement, then further combined with the RBD-up class from the first round of signal-subtraction/ classification based on the apex region of the closed S trimer. This combined class containing 255,909 particles was autorefined producing a map at 4.1Å resolution. Particle CTF refinement/particle polishing and another round of autorefinement were performed before subjected to signal-subtraction and 3D classification using the apex mask. Two major classes in the one-RBD-up conformation were produced and they were subjected to 3D auto-refinement in C1 symmetry using a whole mask, CTF refinement, particle polishing and a final round of 3D auto-refinement. The final reconstructions have 191,067 and 25,370 particles with maps 3.4Å and 4.3Å resolution, respectively. Additional rounds of 3D auto-refinement were performed for each class using different sizes of masks at the top region to improve local resolution. The best density maps were used for model building.

For the Kappa sample, two data sets were collected and initially processed separately. 1,199,999 and 1,718,600 particles were extracted using crYOLO with a trained model from 22,019 and 17,314 images, respectively, from the two sets. The selected particles were subjected to 2D classification, giving 1,112,384 and 1,649,296 particles, respectively. A low-resolution negative-stain reconstruction of the Wuhan-Hu-1 (D614) sample was low-pass-filtered to 40Å as an initial model for 3D classification with C1 symmetry. Three rounds of 3D classification were performed separately for each data set. For data set 1, two major classes were identified, one in the closed conformation and the other in the one-RBD-up conformation. The class in the closed state was 3D auto-refined with C1 symmetry to 4.3Å after CTF refinement and particle polishing. One round of signal-subtraction and 3D classification using the top mask was performed, giving two major classes, once again, one in the closed conformation and the other in the one-RBD-up conformation. This one-RBD-up class was combined with the RBD-up class from the previous third round of 3D classification, and refined to give a map at 4.6Å resolution from 67,997 particles. The class in the closed state with 63,146 particles was refined to produce a map at 3.8Å resolution after CTF refinement and particle polishing. For data set 2, 3D classification identified two classes in the closed conformation and combination of the two gave a map at 4.9Å resolution from 171,733 particles after 3D auto-refinement. One round of signal-subtraction and 3D classification using the top mask was performed, leading to two major classes, one in the closed conformation and the other in the one-RBD-up conformation. Another round of 3D auto-refinement was carried out, giving two maps with 71,425 and 42,005 particles, respectively.

The two classes in the closed state from the two data sets were then combined and subjected to 3D auto-refinement, CTF refinement, particle polishing and a final round of 3D auto-refinement, yielding a map at 4.1Å resolution from 255,909 particles. One round of signal-subtraction 3D and classification with the top mask was performed, leading to a major class in the closed conformation. After 3D auto-refinement with C1/C3 symmetry using a whole mask, CTF refinement, particle polishing and a final round of auto-refinement, this class yielded a final reconstruction at 3.1Å resolution from 123,193 particles. Similarly, the one-RBD-up classes from the two date sets were combined and subjected to 3D auto-refinement,

CTF refinement, particle polishing and 3D auto-refinement, producing a map at 4.0Å resolution from 110,002 particles. One round of signal-subtraction and 3D classification using the top mask was carried out, giving two major classes in distinct one-RBD-up conformations with the RBD projecting at slightly different angles. After 3D auto-refinement with C1 symmetry using a whole mask, CTF refinement, particle polishing and a final round of auto-refinement, the two classes yielded two reconstructions at 3.7Å and 4.3Å resolution from 81,717 and 21,830 particles, respectively. Additional rounds of 3D auto-refinement were performed for each class using different sizes of masks at the top region to improve local resolution. The best density maps were used for model building.

For the Gamma sample, which was not very stable and quite heterogeneous, three independent data sets were collected. 1,652,420, 2,757,190 and 1,893,863 particles were extracted by crYOLO with a trained model from 25,424, 32,569 and 29,163 images, respectively, from the three sets. The selected particles were subjected to 2D classification, giving 1,564,938, 1,904,078 and 1,788,000 particles, respectively. A low-resolution negative-stain reconstruction of the Wuhan-Hu-1 (D614) sample was low-pass-filtered to 40Å as an initial model for 3D classification with C1 symmetry. Two or three rounds of 3D classification were performed separately for the three data sets and each of them produced a major class resembling an S trimer. The three classes were combined and subjected to additional round of 3D classification in C1 symmetry, leading to three good classes. The new classes were auto-refined, giving a map at 4.9Å resolution from 143,872 particles. A round of signal-subtraction and 3D classification using the top mask were performed to produce two major classes in two distinct one-RBD-up conformations with the RBD projecting at slightly different angles. The two classes contain 69,302 and 36,346 particles, respectively and they were subjected to 3D auto-refinement with C1 symmetry using a whole mask, CTF refinement/particle polishing and a final round auto-refinement, yielding two reconstructions at 3.8Å and 4.4Å resolution, respectively. Additional rounds of 3D auto-refinement were performed for each class using different sizes of masks at the top region to improve local resolution. The best density maps were used for model building.

All resolutions were reported from the gold-standard Fourier shell correlation (FSC) using the 0.143 criterion. Density maps were corrected from the modulation transfer function of the K3 detector and sharpened by applying a temperature factor that was estimated using post-processing in RELION. Local resolution was determined using ResMap (*65*) with half-reconstructions as input maps.

Model building

The initial templates for model building used our G614 S trimer structures (PDB ID: 7KRQ and PDB ID: 7KRR; ref(*31*)). Several rounds of manual building were performed in Coot (*66*). The model was then refined in Phenix (*67*) against the 3.1Å (closed), 3.4Å (one-RBD-up 1), 4.3Å (one-RBD-up 2) cryo-EM maps of the Delta variant; the 3.8Å (one-RBD-up 1) and 4.4Å (one-RBD-up 2) cryo-EM maps of the Gamma variant; and the 3.1Å (closed), 3.7Å (one-RBD-up 1) and 4.3Å (one-RBD-up 2) cryo-EM maps of the Kappa variant. Iteratively, refinement was performed in both Refmac (real space refinement) and ISOLDE (*68*), and the Phenix refinement strategy included minimization_global, local_grid_search, and adp, with rotamer, Ramachandran, and reference-model restraints, using 7KRQ and 7KRR as the reference model. The refinement statistics are summarized in Table S3. Structural biology applications used in this project were compiled and configured by SBGrid (*69*).

Supplementary materials

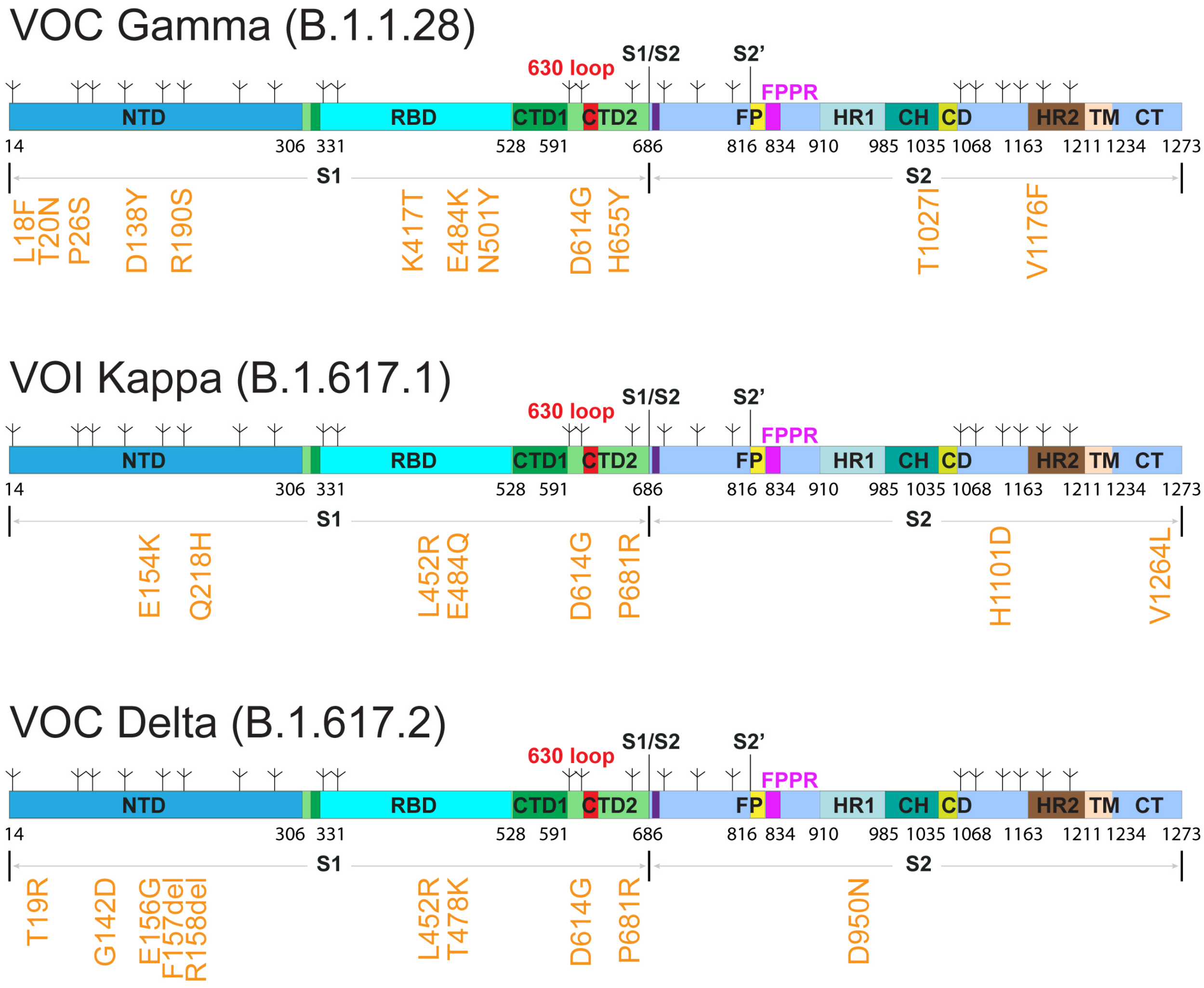

Figure S1. Schematic representation of full-length SARS-CoV-2 spike (S) proteins from the VOC Gamma (B.1.1.28), VOI Kappa (B.1.617.1) and VOC Delta (B.1.617.2). The sequences are derived from the Gamma (hCoV-19/Brazil/AM-992/2020), Kappa (hCoV-19/India/MH-NEERI-NGP-40449/2021) and Delta (hCoV-19/India/GJ-GBRC619/2021) variants. Segments of S1 and S2 include: NTD, N-terminal domain; RBD, receptor-binding domain; CTD1, C-terminal domain 1; CTD2, C-terminal domain 2; 630 loop; S1/S2, S1/S2 cleavage site; S2', S2' cleavage site; FP, fusion peptide; FPPR, fusion peptide proximal region; HR1, heptad repeat 1; CH, central helix region; CD, connector domain; HR2, heptad repeat 2; TM, transmembrane anchor; CT, cytoplasmic tail; and tree-like symbols for glycans. Positions of all mutations (from the amino-acid sequence of Wuhan-Hu-1) are shown in orange text.

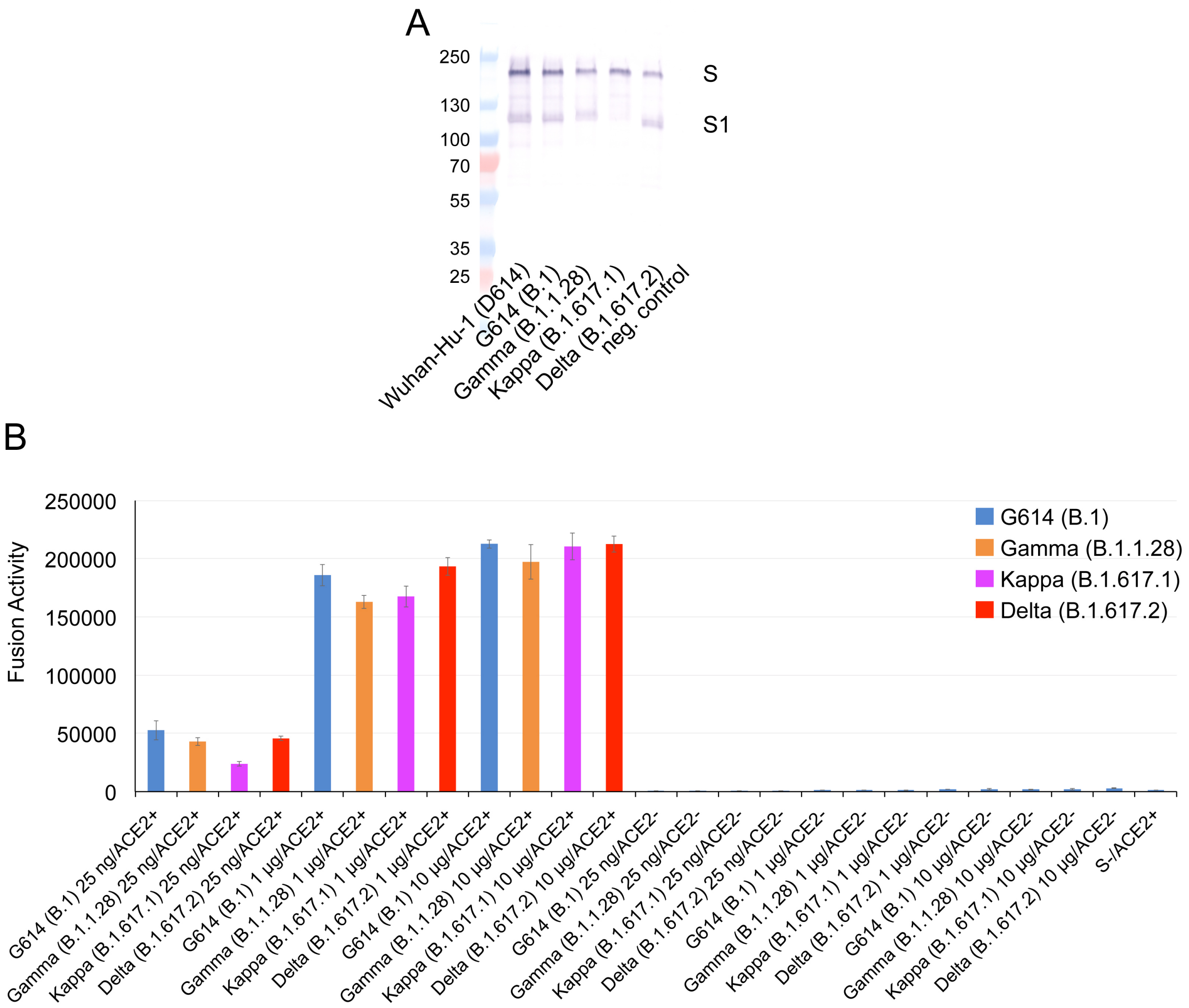

Figure S2. Expression and cell-cell fusion of SARS-CoV-2 variant S proteins. (**A**) Expression and processing of the full-length S constructs in HEK293 cells. S samples prepared from HEK293 cells transiently transfected with 10 μg of the full-length S expression plasmids were detected by anti-RBD polyclonal antibodies. Bands for the uncleaved S and S1 fragment are indicated. (**B**) HEK293T cells transfected with the untagged, full-length S protein expression plasmids were fused with ACE2-expressing cells. Cell-cell fusion led to reconstitution of α and ω fragments of β-galactosidase yielding an active enzyme, and the fusion activity was then quantified by a chemiluminescent assay. No ACE2 and no S were negative controls.

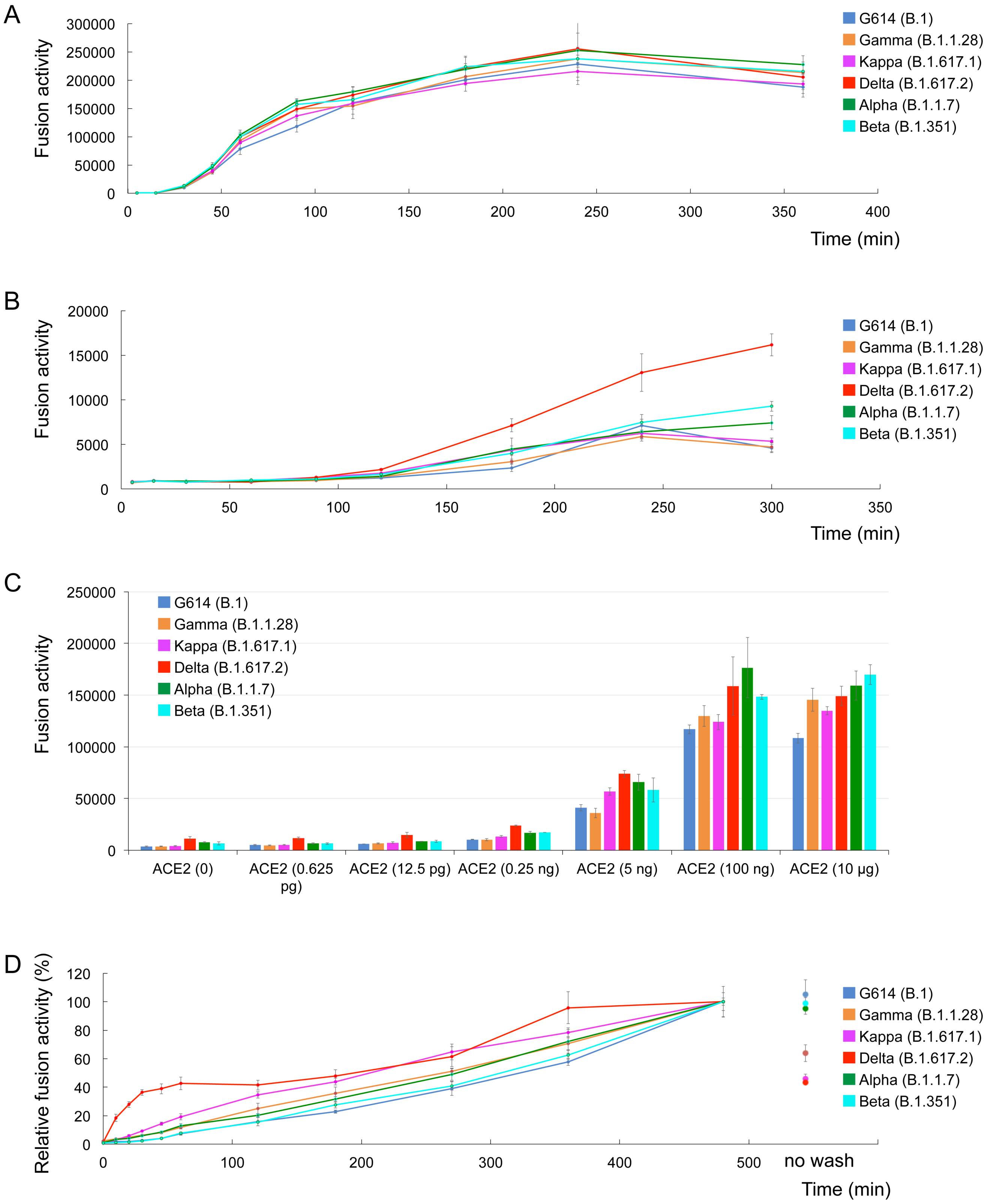

Figure S3. Comparison of membrane fusion mediated by the Delta variant with that by other variants. (**A**) Time-course of cell-cell fusion mediated by various full-length S proteins

expressed at the 10 μg transfection level, as indicated, with HEK293 cells transfected with 10 μg of ACE2. (**B**) Time-course of cell-cell fusion mediated by various full-length S proteins (10 μg transfection), as indicated, with HEK293 cells with no exogenous ACE2. (**C**) Cell-cell fusion mediated by various full-length S proteins with HEK293 cells transfected with various levels (0-10 μg) of ACE2 expression constructs. (**D**) Time course of infection HEK293-ACE2 cells by MLV-based pseudotyped viruses using various SARS-CoV-2 variant S constructs containing a CT deletion in a single cycle. Infection was initiated by mixing viruses and target cells, and viruses were washed out at each time point as indicated. Selected data points are highlighted in Fig. 1. The experiments were repeated at least three times with independent samples giving similar results.

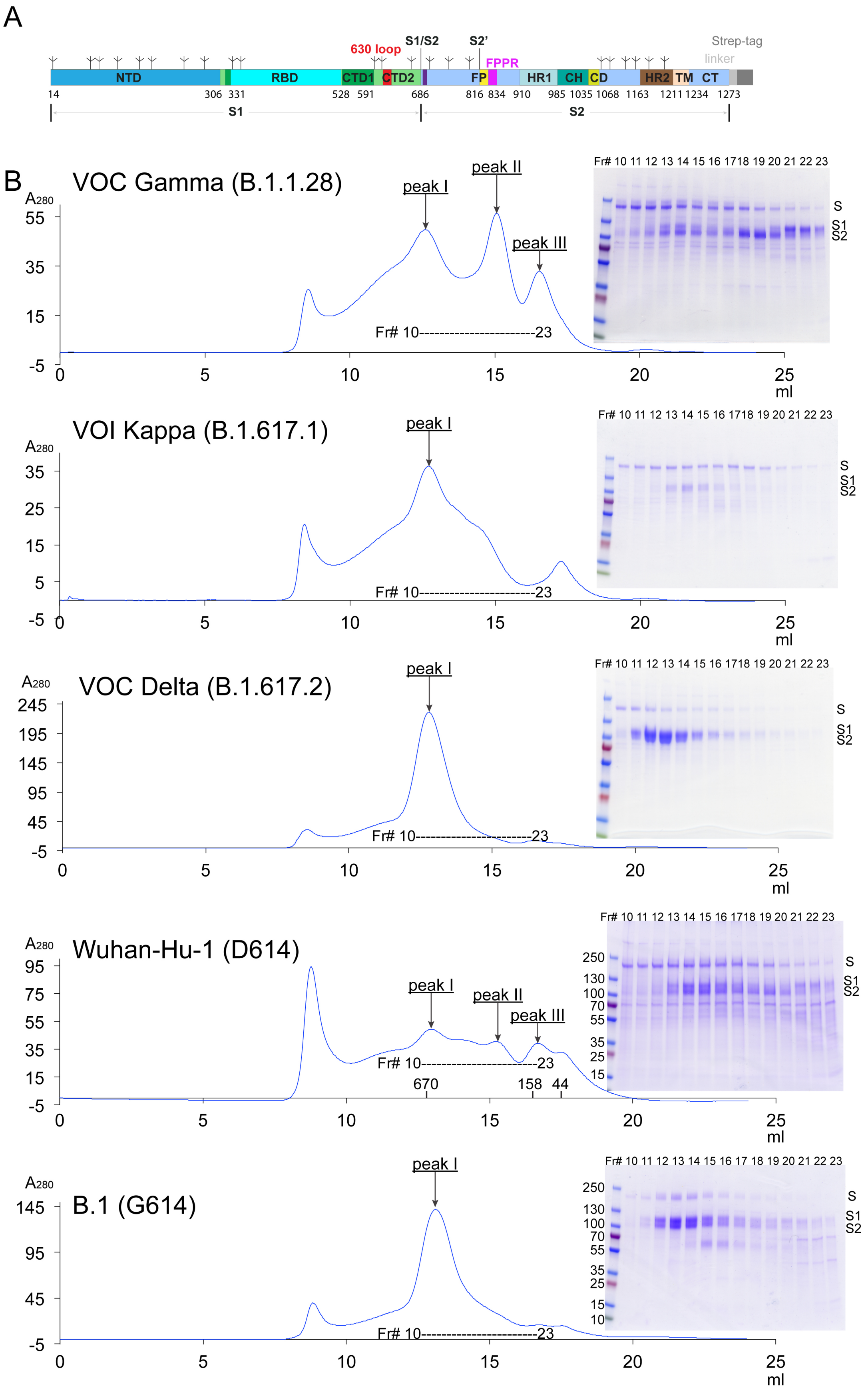
A
630 loop
S1/S2
S2'
FPPR
Strep-tag
linker
NTD
RBD
CTD1
CTD2
FP
HR1
CH
CD
HR2
TM
CT
14 306 331 528 591 686 816 834 910 985 1035 1068 1163 1211 1234 1273
S1
S2
B
VOC Gamma (B.1.1.28)
peak I
peak II
peak III
Fr# 10---------------------23
VOI Kappa (B.1.617.1)
VOC Delta (B.1.617.2)
Wuhan-Hu-1 (D614)
670 158 44
B.1 (G614)
A280
ml
Fr# 10 11 12 13 14 15 16 17 18 19 20 21 22 23
S
S1
S2
250 130 100 70 55 35 25 15 10

Figure S4. Production of full-length S protein from the Gamma, Kappa and Delta variants. (**A**) A strep-tag was fused to the C-terminus of the full-length S protein by a flexible linker. (**B**) The full-length S proteins were extracted and purified in detergent DDM, and further resolved by gel-filtration chromatography on a Superose 6 column. Peak I, the prefusion S trimer; peak II, the postfusion S2 trimer; and peak III, the dissociated monomeric S1. Inset, peak fractions were analyzed by Coomassie stained SDS-PAGE. Labeled bands are S, S1 and S2. Fr#, fraction number. Each experiment was repeated at least three times independently with similar results. The data for the preparations from the Wuhan-Hu-1 (D614) and B.1 (G614), published previously (*28, 31*), are included for convenient comparison.

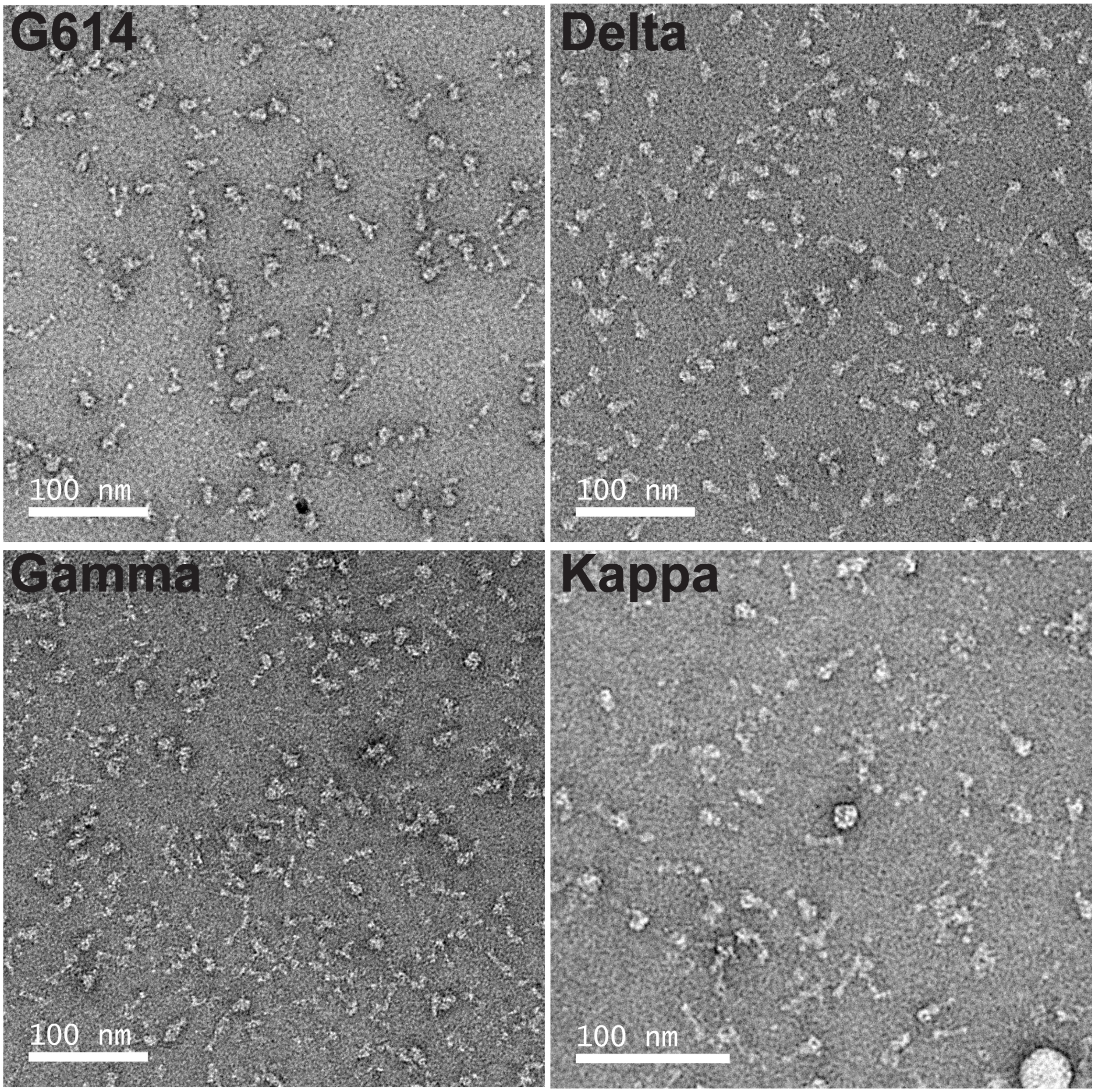

Figure S5. Representative images of various full-length S trimers by negative stain EM.

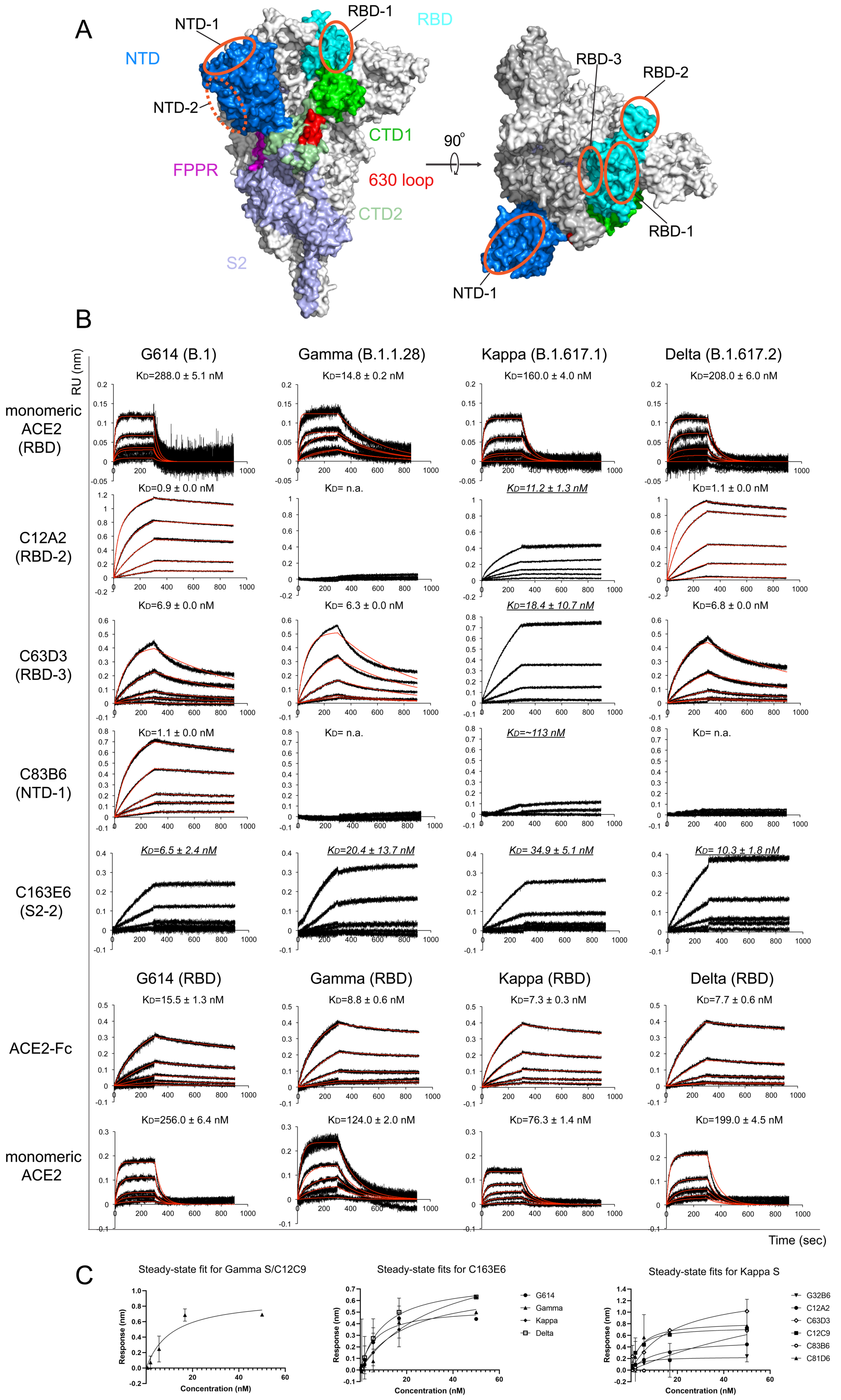
A
NTD-1
NTD
NTD-2
RBD-1
RBD
CTD1
FPPR
630 loop
CTD2
S2
90°
RBD-3
RBD-2
RBD-1
NTD-1
B
RU (nm)
G614 (B.1)
Gamma (B.1.1.28)
Kappa (B.1.617.1)
Delta (B.1.617.2)
monomeric ACE2 (RBD)
KD=288.0 ± 5.1 nM
KD=14.8 ± 0.2 nM
KD=160.0 ± 4.0 nM
KD=208.0 ± 6.0 nM
C12A2 (RBD-2)
KD=0.9 ± 0.0 nM
KD= n.a.
KD=11.2 ± 1.3 nM
KD=1.1 ± 0.0 nM
C63D3 (RBD-3)
KD=6.9 ± 0.0 nM
KD= 6.3 ± 0.0 nM
KD=18.4 ± 10.7 nM
KD=6.8 ± 0.0 nM
C83B6 (NTD-1)
KD=1.1 ± 0.0 nM
KD= n.a.
KD=~113 nM
KD= n.a.
C163E6 (S2-2)
KD=6.5 ± 2.4 nM
KD=20.4 ± 13.7 nM
KD= 34.9 ± 5.1 nM
KD= 10.3 ± 1.8 nM
G614 (RBD)
Gamma (RBD)
Kappa (RBD)
Delta (RBD)
ACE2-Fc
KD=15.5 ± 1.3 nM
KD=8.8 ± 0.6 nM
KD=7.3 ± 0.3 nM
KD=7.7 ± 0.6 nM
monomeric ACE2
KD=256.0 ± 6.4 nM
KD=124.0 ± 2.0 nM
KD=76.3 ± 1.4 nM
KD=199.0 ± 4.5 nM
Time (sec)
C
Steady-state fit for Gamma S/C12C9
Steady-state fits for C163E6
Steady-state fits for Kappa S
Response (nm)
Concentration (nM)
G614
Gamma
Kappa
Delta
G32B6
C12A2
C63D3
C12C9
C83B6
C81D6

Figure S6. Additional antigenic properties of the purified full-length SARS-CoV-2 S proteins. (**A**) Antibody competition clusters as described in ref(*32*). Surface regions of the S trimer targeted by antibodies on S1 are highlighted by orange ellipses, including RBD-1, RBD-2, RBD-3, NTD-1 and NTD-2. The exact location of NTD-2 is uncertain and therefore marked with a dashed line. (**B**) Binding analysis of the prefusion S trimers from G614, Gamma, Kappa and Delta variants with soluble ACE2 constructs was performed by bio-layer interferometry (BLI). For ACE2 binding, the purified S proteins were immobilized on AR2G biosensors and dipped into the wells containing ACE2 at various concentrations. For antibody binding, various antibodies were immobilized to AHC biosensors and dipped into the wells containing each purified S protein at different concentrations. Binding kinetics were evaluated using a 1:1 Langmuir model except for antibody C12A2 targeting the RBD-2, which was analyzed by a bivalent binding model. All K_D values for multivalent interactions with antibody IgG or dimeric ACE2 and trimeric S protein are the apparent affinities with avidity effects. The sensorgrams are in black and the fits in red. Binding constants highlighted by underlines were estimated by steady-state analysis as described in the Methods. RU, response unit. Binding constants are also summarized here and in Table S1. All experiments were repeated at least twice with essentially identical results. (**C**) Steady-state analysis by plotting steady-state responses against concentrations. Kd values were derived from the fits.

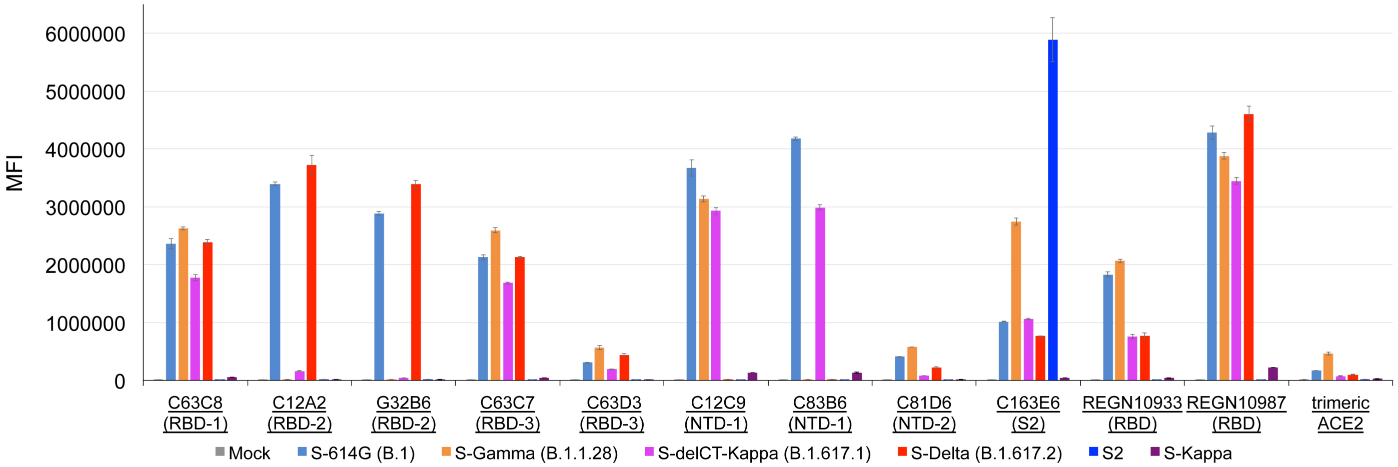

Figure S7. Antigenic properties of the cell-surface S proteins assessed by flow cytometry. Antibody and ACE2 binding to the full-length S proteins of the G614, Gamma, Kappa and Delta variants, as well as an S2 construct expressed on the cell surfaces analyzed by flow cytometry. An S-delCT-Kappa with the C-terminal 19 residues deleted was included because the expression level of the full-length S construct (S-Kappa) was low. The antibodies and their targets are indicated. Two therapeutic anti-RBD antibodies by Regeneron, REGN10933 (casirivimab) and REGN10987 (imdevimab), were also included in this assay. A designed ACE2-based inhibitor $ACE2_{615}$-foldon-T27W was used for detecting receptor binding (*33*). MFI, mean fluorescent intensity. The error bars represent standard errors of mean from measurements using three independently transfected cell samples. The flow cytometry assays were repeated three times with essentially identical results.

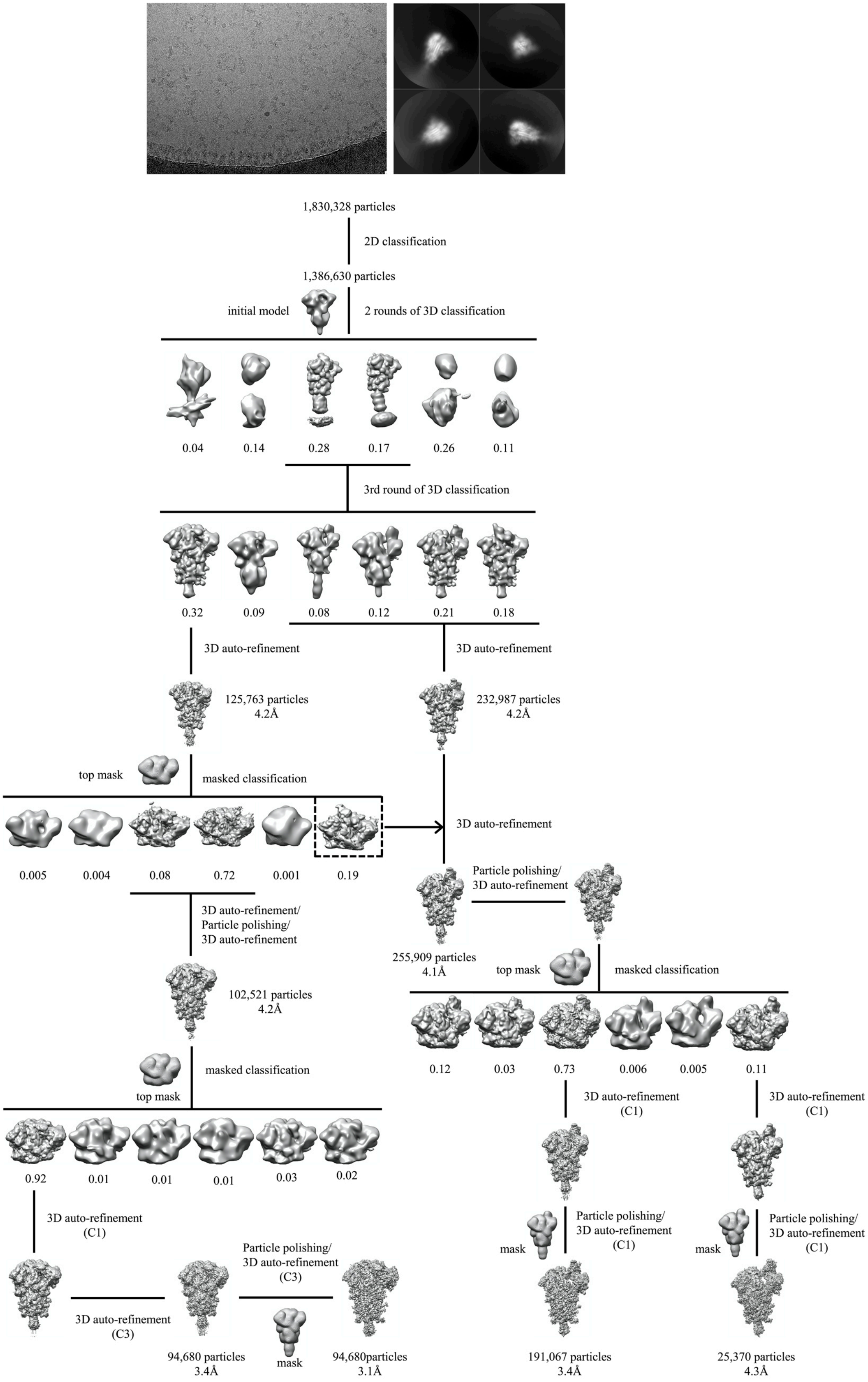
1,830,328 particles
2D classification
1,386,630 particles
initial model
2 rounds of 3D classification
0.04
0.14
0.28
0.17
0.26
0.11
3rd round of 3D classification
0.32
0.09
0.08
0.12
0.21
0.18
3D auto-refinement
3D auto-refinement
125,763 particles
4.2Å
232,987 particles
4.2Å
top mask
masked classification
3D auto-refinement
0.005
0.004
0.08
0.72
0.001
0.19
Particle polishing/
3D auto-refinement
3D auto-refinement/
Particle polishing/
3D auto-refinement
255,909 particles
4.1Å
top mask
masked classification
102,521 particles
4.2Å
0.12
0.03
0.73
0.006
0.005
0.11
masked classification
top mask
3D auto-refinement
(C1)
3D auto-refinement
(C1)
0.92
0.01
0.01
0.01
0.03
0.02
3D auto-refinement
(C1)
Particle polishing/
3D auto-refinement
(C1)
Particle polishing/
3D auto-refinement
(C1)
mask
mask
Particle polishing/
3D auto-refinement
(C3)
3D auto-refinement
(C3)
94,680 particles
3.4Å
mask
94,680particles
3.1Å
191,067 particles
3.4Å
25,370 particles
4.3Å

Figure S8. Cryo-EM analysis of the Delta S trimer. Top, representative micrograph**,** and 2D averages (box dimension: 396Å) of the cryo-EM particle images of the Delta S trimer. Bottom, data processing workflow for structure determination.

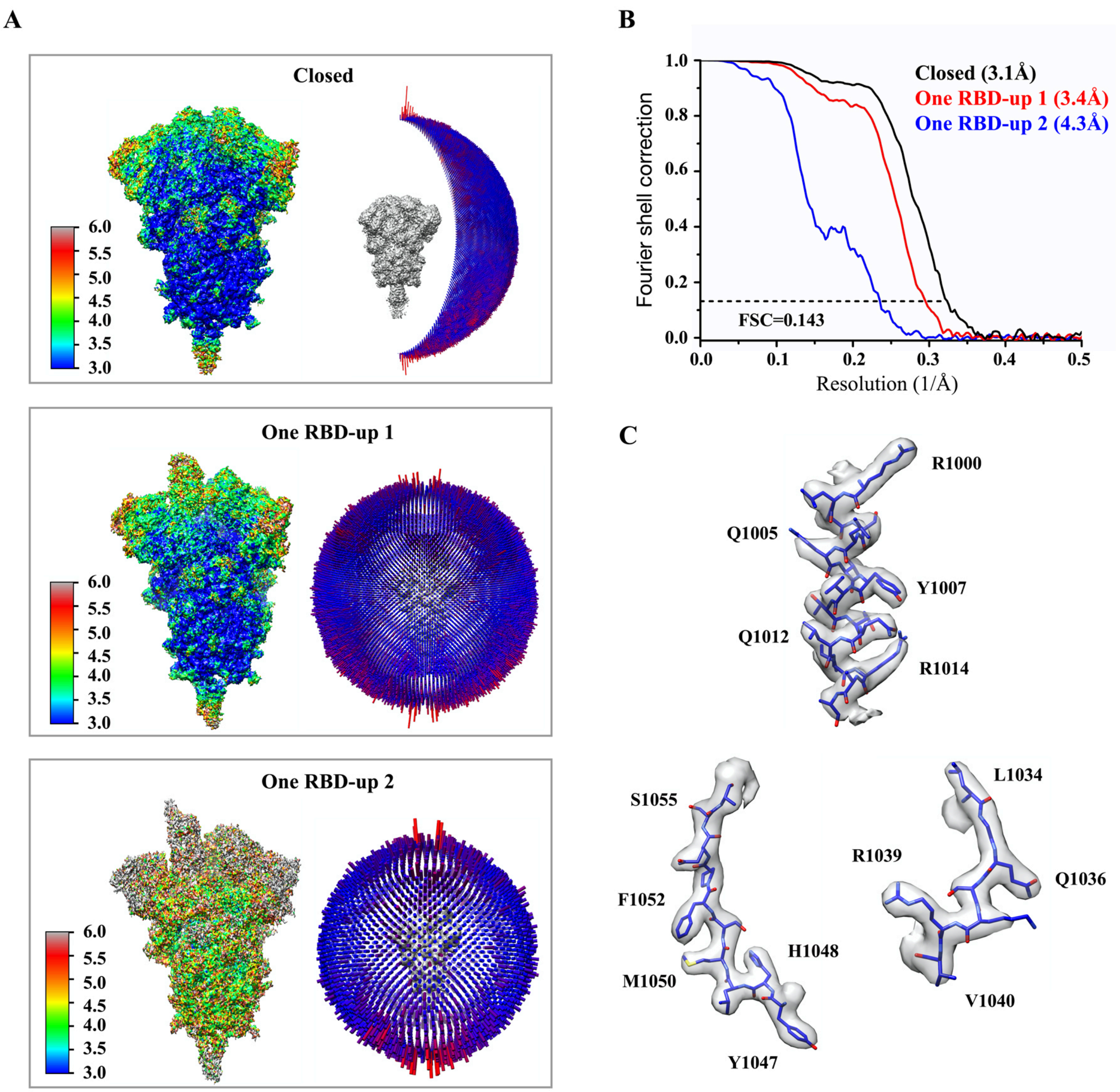

Figure S9. Analysis of the Delta S trimer structure. (**A**) 3D reconstructions of the Delta trimer preparation in the closed, two one-RBD-up conformations, respectively, are colored according to local resolution estimated by ResMap. Angular distribution of the cryo-EM particles used in each reconstruction is shown in the side view of the EM map. (**B**) Gold standard FSC curves of the three refined 3D reconstructions of the Delta S trimer. (**C**) Representative density in gray surface from EM maps with a resolution better than 3.5Å.

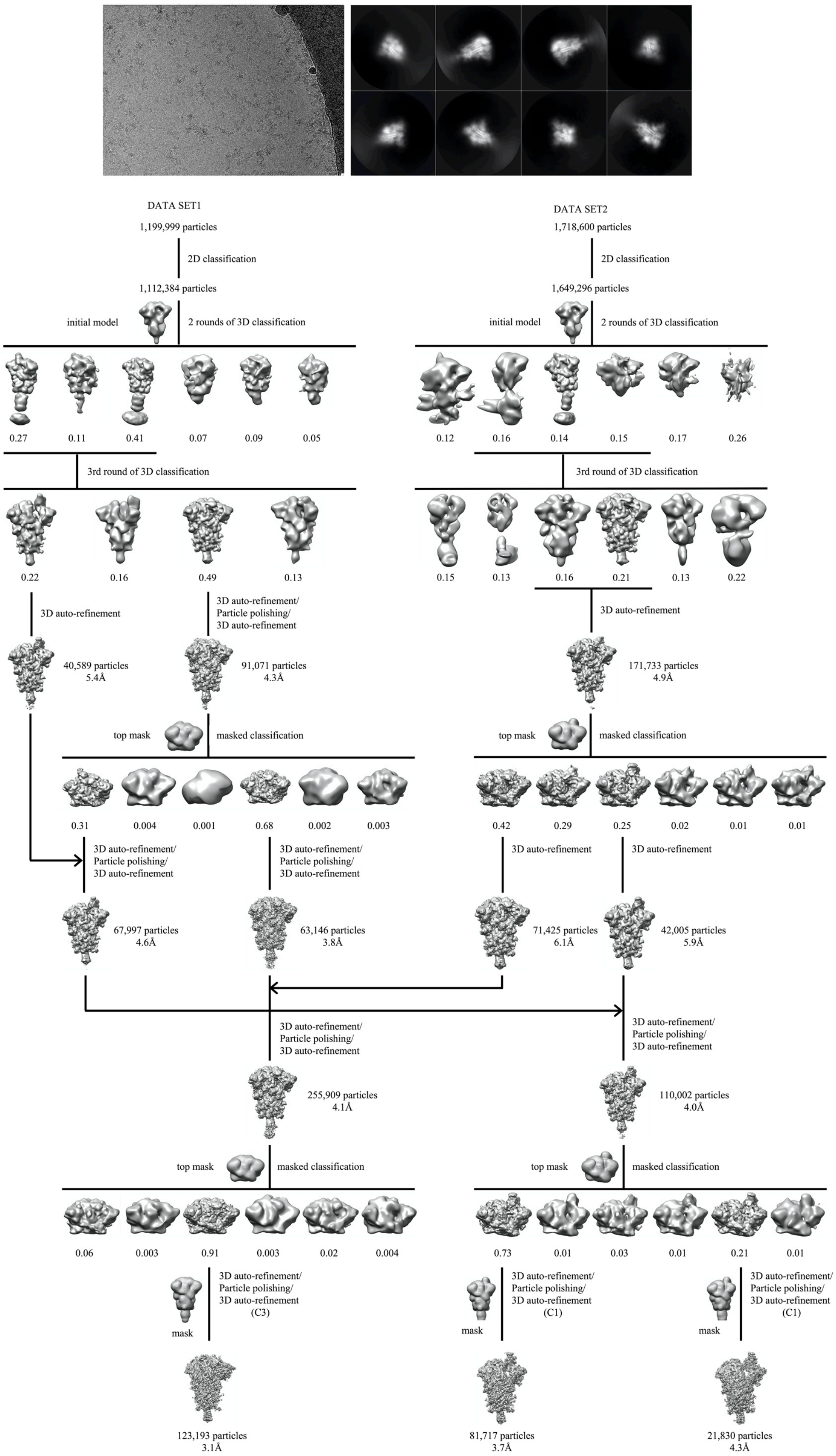
DATA SET1
1,199,999 particles
2D classification
1,112,384 particles
initial model
2 rounds of 3D classification
0.27
0.11
0.41
0.07
0.09
0.05
3rd round of 3D classification
0.22
0.16
0.49
0.13
3D auto-refinement
40,589 particles
5.4Å
3D auto-refinement/
Particle polishing/
3D auto-refinement
91,071 particles
4.3Å
top mask
masked classification
0.31
0.004
0.001
0.68
0.002
0.003
3D auto-refinement/
Particle polishing/
3D auto-refinement
67,997 particles
4.6Å
3D auto-refinement/
Particle polishing/
3D auto-refinement
63,146 particles
3.8Å
DATA SET2
1,718,600 particles
2D classification
1,649,296 particles
initial model
2 rounds of 3D classification
0.12
0.16
0.14
0.15
0.17
0.26
3rd round of 3D classification
0.15
0.13
0.16
0.21
0.13
0.22
3D auto-refinement
171,733 particles
4.9Å
top mask
masked classification
0.42
0.29
0.25
0.02
0.01
0.01
3D auto-refinement
71,425 particles
6.1Å
3D auto-refinement
42,005 particles
5.9Å
3D auto-refinement/
Particle polishing/
3D auto-refinement
255,909 particles
4.1Å
3D auto-refinement/
Particle polishing/
3D auto-refinement
110,002 particles
4.0Å
top mask
masked classification
0.06
0.003
0.91
0.003
0.02
0.004
top mask
masked classification
0.73
0.01
0.03
0.01
0.21
0.01
mask
3D auto-refinement/
Particle polishing/
3D auto-refinement
(C3)
123,193 particles
3.1Å
mask
3D auto-refinement/
Particle polishing/
3D auto-refinement
(C1)
81,717 particles
3.7Å
mask
3D auto-refinement/
Particle polishing/
3D auto-refinement
(C1)
21,830 particles
4.3Å

Figure S10. Cryo-EM analysis of the Kappa S trimer. Top, representative micrograph**,** and 2D averages (box dimension: 396Å) of the cryo-EM particle images of the Kappa S trimer. Bottom, data processing workflow for structure determination.

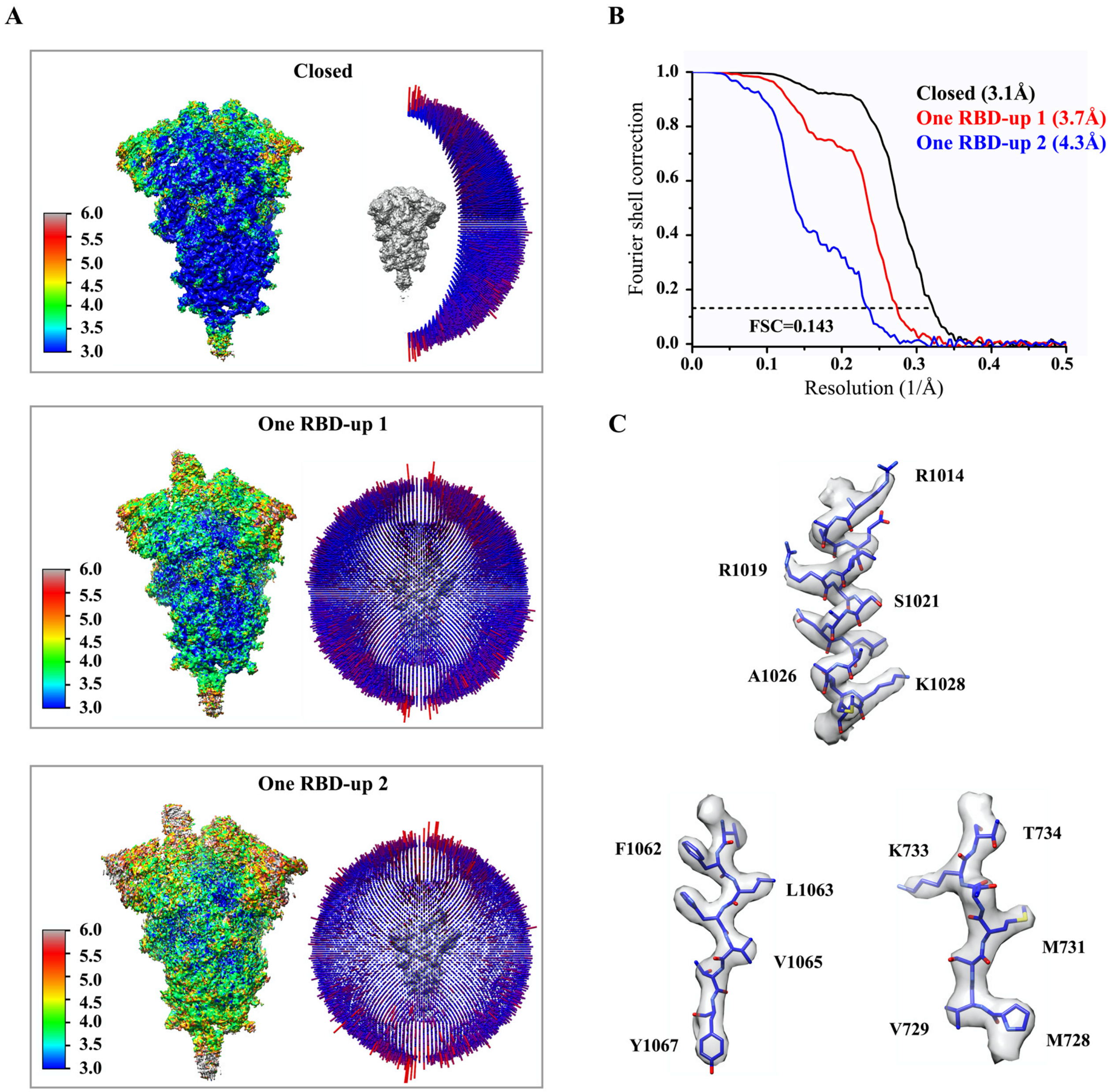

Figure S11. Analysis of the Kappa S trimer structure. (**A**) 3D reconstructions of the Kappa S trimer preparation in the closed and two one-RBD-up conformations, respectively, are colored according to local resolution estimated by ResMap. Angular distribution of the cryo-EM particles used in reconstruction for the closed conformation is shown in the side view of the EM map. (**B**) Gold standard FSC curves of the refined 3D reconstructions of the Kappa S trimer. (**C**) Representative density in gray surface from EM maps with a resolution better than 3.5Å.

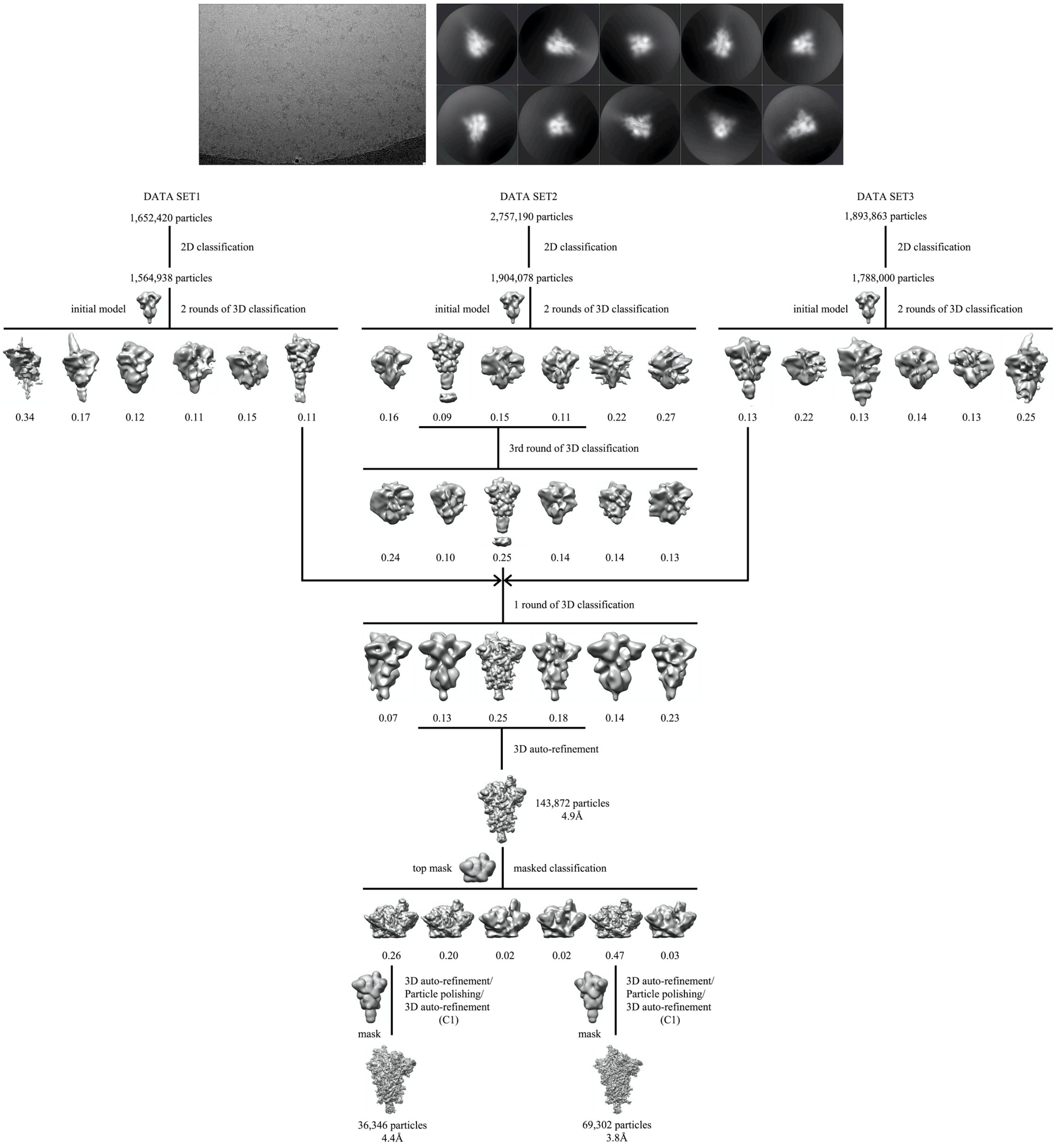

Figure S12. Cryo-EM analysis of the Gamma S trimer. Top, representative micrograph**,** and 2D averages (box dimension: 396Å) of the cryo-EM particle images of the Gamma S trimer. Bottom, data processing workflow for structure determination.

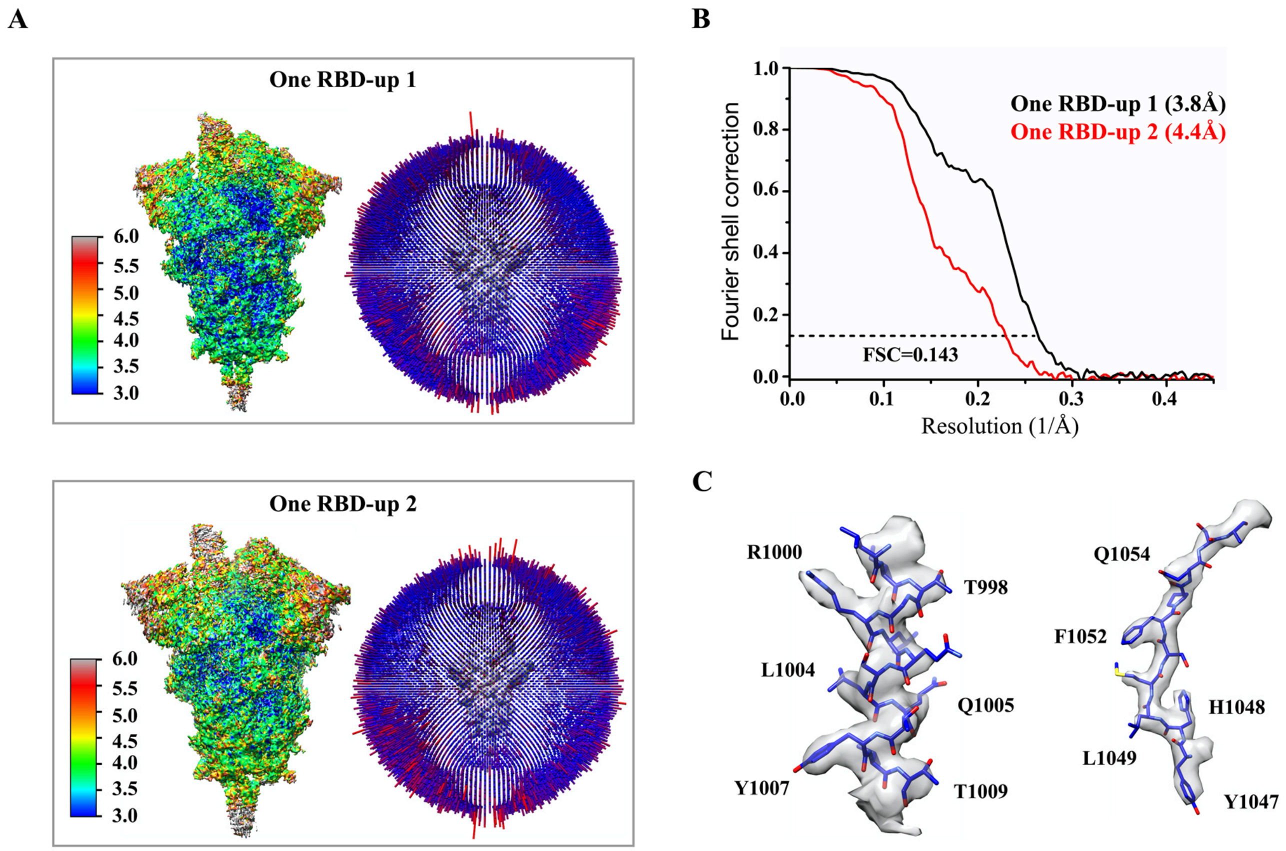

Figure S13. Analysis of the Gamma S trimer structure. (**A**) 3D reconstructions of the Gamma S trimer preparation in the two one-RBD-up conformations, respectively, are colored according to local resolution estimated by ResMap. Angular distribution of the cryo-EM particles used in reconstruction for the closed conformation is shown in the side view of the EM map. (**B**) Gold standard FSC curves of the refined 3D reconstructions of the Gamma S trimer. (**C**) Representative density in gray surface from the 3.8Å EM map.

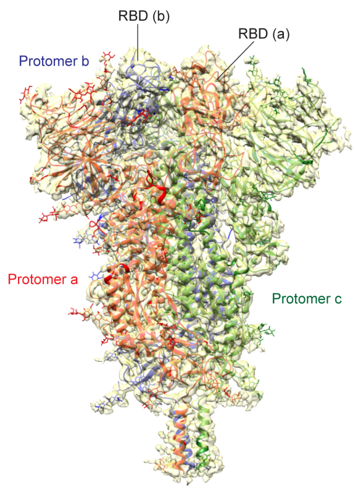

Delta closed conformation
(31%; 3.1Å)

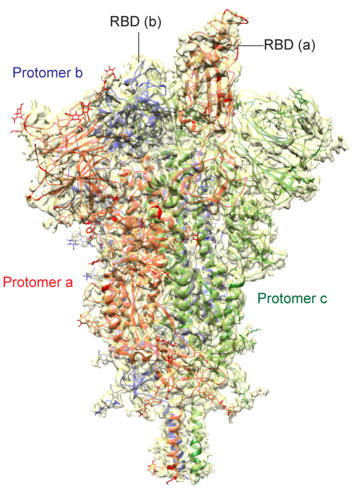

Delta one-RBD-up conformation 1
(61%; 3.4Å)

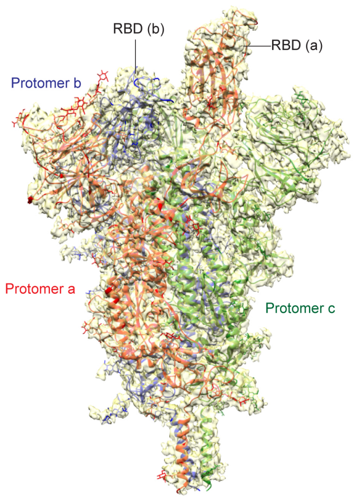

Delta one-RBD-up conformation 2
(8%; 4.3Å)

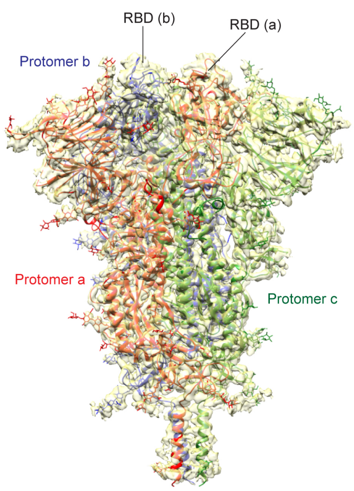

Kappa closed conformation
(54%; 3.1Å)

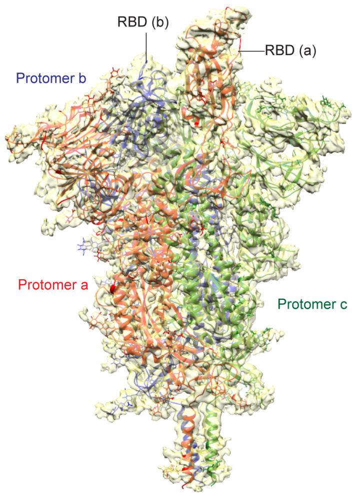

Kappa one-RBD-up conformation 1
(36%; 3.7Å)

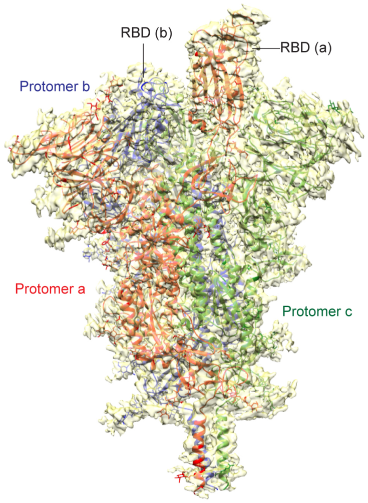

Kappa one-RBD-up conformation 2
(10%; 4.3Å)

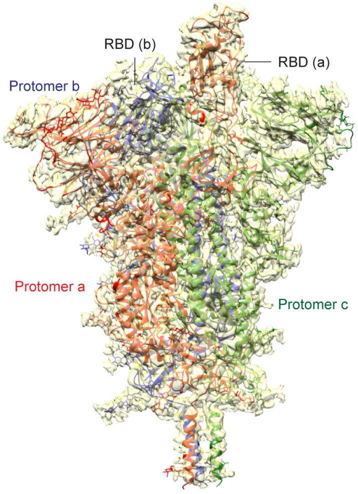

Gamma one-RBD-up conformation 1
(66%; 3.8Å)

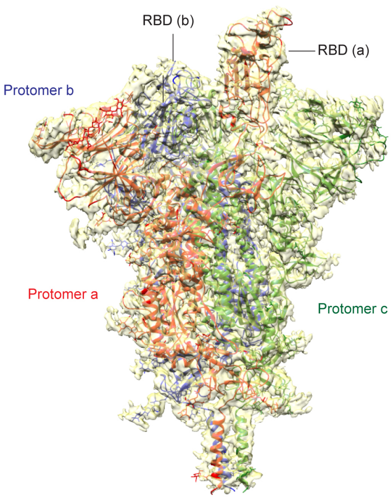

Gamma one-RBD-up conformation 2
(34%; 4.4Å)

Figure S14. Cryo-EM structures of the full-length S proteins of the Delta, Kappa and Gamma variants. Three structures of the Delta S trimer, representing a closed prefusion conformation and two distinct one-RBD-up conformations, were modeled based on corresponding cryo-EM density maps at 3.1-4.3Å resolution. Three structures of the Kappa S trimer, representing a closed prefusion conformation and two distinct one-RBD-up conformations, were modeled based on corresponding cryo-EM density maps at 3.1-4.3Å resolution. Two structures of the Gamma S trimer, representing two distinct one-RBD-up conformations, were modeled based on corresponding cryo-EM density maps at 3.8-4.4Å resolution. Three protomers (a, b, c) are colored in red, blue and green, respectively. RBD locations are indicated. Particle percentage for each class in the data processing is also indicated, but it may not accurately reflect the conformation distribution of the S trimer in solution.

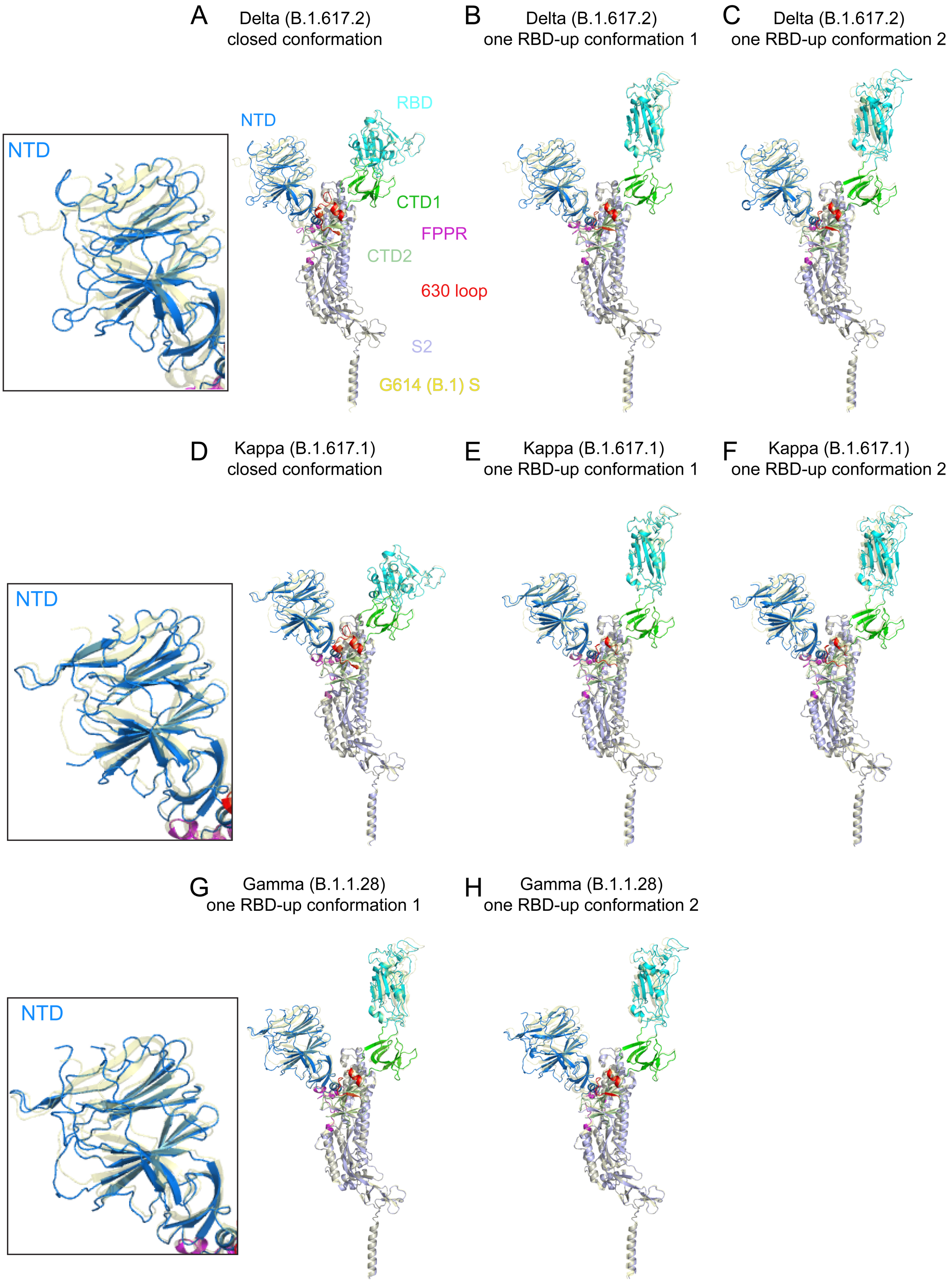
A
Delta (B.1.617.2)
closed conformation
B
Delta (B.1.617.2)
one RBD-up conformation 1
C
Delta (B.1.617.2)
one RBD-up conformation 2
NTD
NTD
RBD
CTD1
FPPR
CTD2
630 loop
S2
G614 (B.1) S
D
Kappa (B.1.617.1)
closed conformation
E
Kappa (B.1.617.1)
one RBD-up conformation 1
F
Kappa (B.1.617.1)
one RBD-up conformation 2
NTD
G
Gamma (B.1.1.28)
one RBD-up conformation 1
H
Gamma (B.1.1.28)
one RBD-up conformation 2
NTD

Figure S15. Superposition of the variant trimer structures and the G614 structure. (**A**)-(**C**) Side views of superposition of the closed conformation and two distinct one-RBD-up conformations of the Delta S (various colors) in ribbon diagram, aligned by the S2 portion with the closed prefusion structure and the one-RBD-up conformation of the G614 S (yellow), respectively. The positions of the RBD-down and three different RBD-up conformations are indicated. (**D**)-(**F**) Comparison of the Kappa S (various colors) and G614 trimer (yellow) structures in the corresponding conformations, when aligned by S2. (**G**) and (**H**) Comparison of the Gamma S (various colors) and G614 trimer (yellow) structures in the one-RBD-up conformation, when aligned by S2.

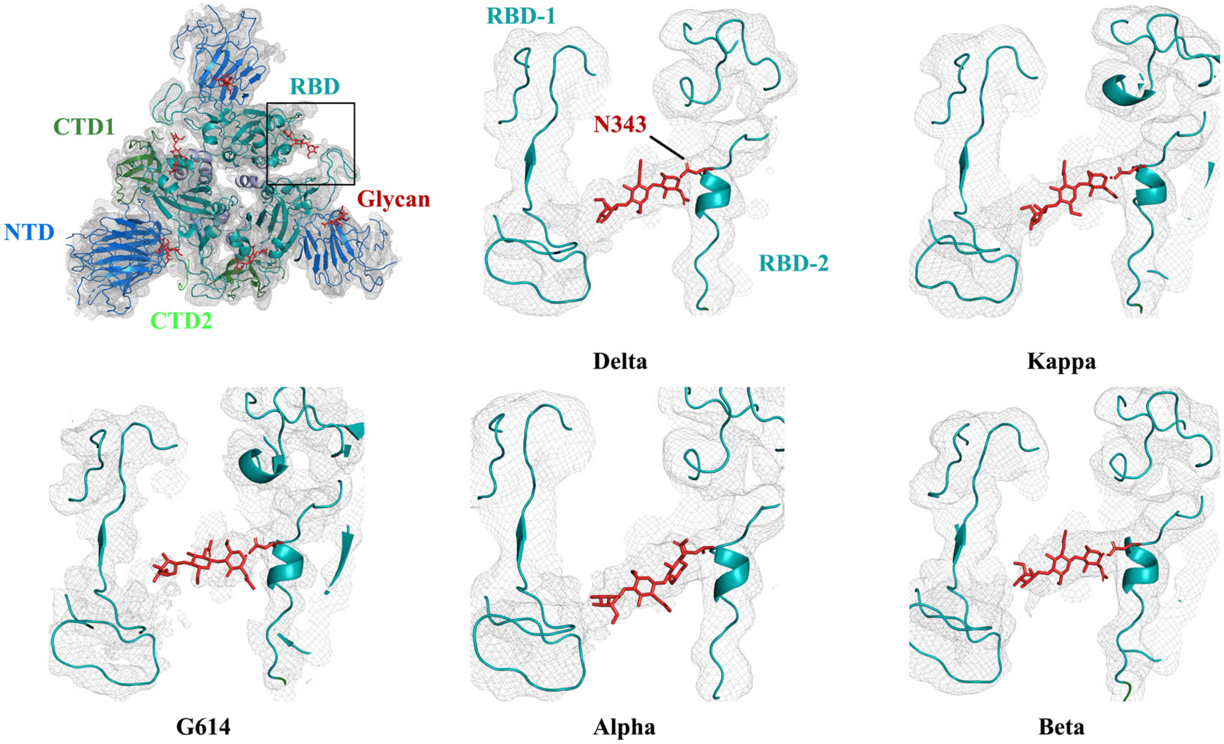

Figure S16. Comparison of the density of the N-linked glycan at Asn343 in the variant trimers. The EM maps of G614, Delta, Kappa, Alpha and Beta trimers in the closed prefusion conformation are compared at the same resolution (3.6Å) and the same contour level.

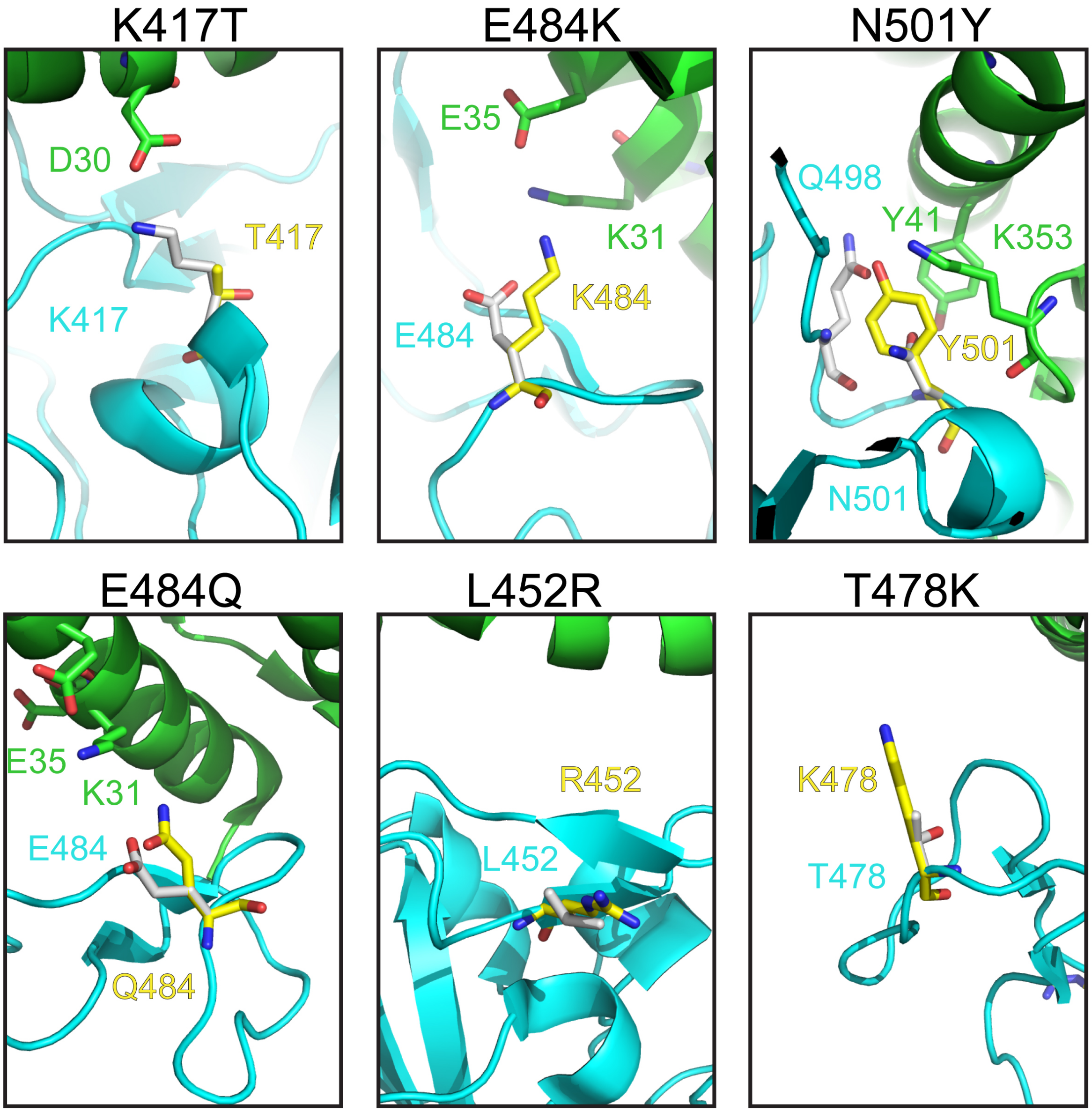

Figure S17. Modeled interface between the RBD and ACE2. The interface between ACE2 in ribbon diagram in green and RBD in cyan from the complex structure (PDB ID: 6M0J; ref(*40*)). Modeled K417T, E484K, N501Y, E484Q, L452R and T478K are shown as sticks. Panels E484K and N501Y were published previously in ref(*2*).

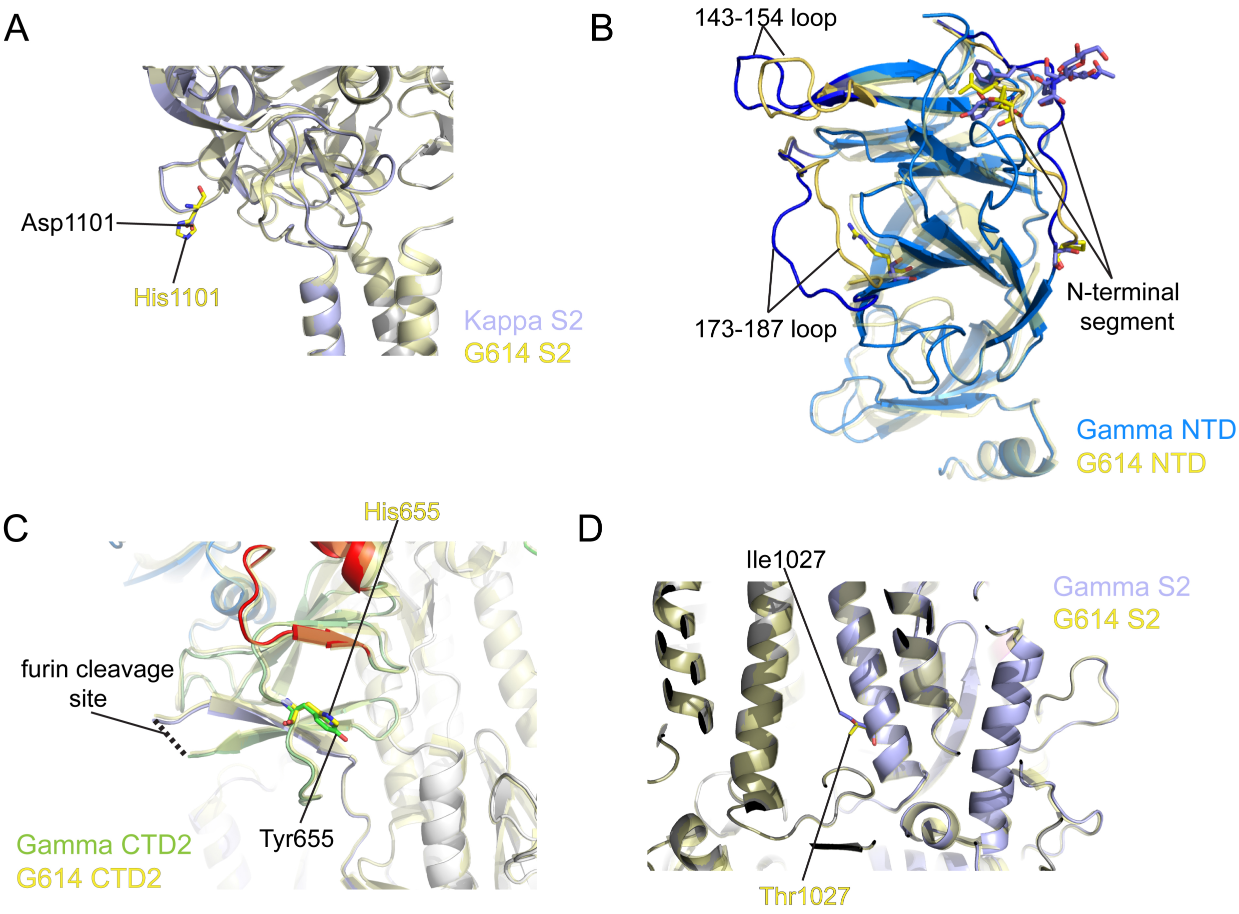

Figure S18. Structural impact of the mutations in the variants. (**A**) Superposition of the structure of the Kappa S trimer in ribbon representation with the structure of the G614 S in yellow, showing the regions near the mutation H1101D. (**B**) Superposition of the NTD structures of the Gamma (blue) and G614 (yellow) S trimers in the one-RBD-up conformation. Replacements of the N-terminal segment, 143-154 and 173-187 loops are indicated. (**C**) and (**D**) Superposition of the structure of the Gamma S trimer in ribbon representation with the structure of the G614 S in yellow aligned by S2, showing the region near mutations H655Y and T1027I. All mutations are shown as sticks.

Table S1. Binding constants of S-ACE2 interaction

		K_D[a] (M)	K_D Error	ka (1/Ms)	ka2	ka Error	ka2 Error	kdis (1/s)	kdis2	kdis Error	kdis2 Error
**ACE2-Fc (RBD)**	G614 S	1.96E-08	2.52E-09	2.64E+04	6.00E-01	2.29E+03	7.68E+00	5.18E-04	8.98E-02	4.91E-05	1.13E+00
	Gamma S	2.41E-09	2.76E-10	9.45E+04	2.09E+01	4.95E+03	2.16E+00	2.28E-04	7.22E-01	2.31E-05	1.70E-01
	Kappa S	7.51E-09	8.99E-10	5.14E+04	4.29E-01	3.36E+03	5.44E-01	3.86E-04	3.85E-02	3.88E-05	5.59E-02
	Delta S	1.76E-07	5.64E-08	2.62E+04	1.06E-01	1.13E+03	4.38E-02	4.60E-03	1.83E-03	1.46E-03	4.41E-04
	G614 RBD	1.55E-08	1.30E-09	2.83E+04	2.27E-03	2.14E+03	1.07E+03	4.40E-04	2.77E+00	1.60E-05	1.30E+06
	Gamma RBD	8.76E-09	5.67E-10	3.95E+04	4.08E+00	1.21E+03	8.31E+00	3.46E-04	5.48E-01	1.97E-05	1.11E+00
	Kappa RBD	7.34E-09	2.82E-10	4.02E+04	3.31E+00	1.10E+03	1.05E+02	2.95E-04	1.07E+00	7.95E-06	3.38E+01
	Delta RBD	7.66E-09	5.93E-10	2.64E+04	4.17E-02	1.72E+03	9.46E-01	2.02E-04	9.36E-02	8.40E-06	2.10E+00
**Monomeric ACE2 (RBD)**	G614 S	2.88E-07	5.13E-09	1.00E+05		1.71E+03		2.89E-02		1.48E-04	
	Gamma S	1.48E-08	1.92E-10	2.20E+05		2.75E+03		3.26E-03		1.10E-05	
	Kappa S	1.60E-07	4.00E-09	9.71E+04		2.31E+03		1.55E-02		1.22E-04	
	Delta S	2.08E-07	6.04E-09	7.38E+04		2.05E+03		1.53E-02		1.33E-04	
	G614 RBD	2.56E-07	6.44E-09	8.15E+04		1.96E+03		2.09E-02		1.55E-04	
	Gamma RBD	1.24E-07	1.96E-09	5.68E+04		8.58E+02		7.06E-03		3.16E-05	
	Kappa RBD	7.63E-08	1.38E-09	1.59E+05		2.75E+03		1.21E-02		6.42E-05	
	Delta RBD	1.99E-07	4.46E-09	6.16E+04		1.32E+03		1.23E-02		8.06E-05	
**C63C8 (RBD-1)**	G614 S	7.05E-09	4.38E-11	8.92E+04		3.64E+02		6.29E-04		2.95E-06	
	Gamma S	2.78E-09	1.56E-11	2.03E+05		8.41E+02		5.65E-04		2.13E-06	
	Kappa S	1.90E-10	1.12E-11	1.31E+05		5.07E+02		2.50E-05		1.47E-06	
	Delta S	4.76E-09	1.97E-11	1.95E+05		6.97E+02		9.29E-04		1.94E-06	
**G32B6 (RBD-2)**	G614 S	7.05E-10	2.13E-11	2.12E+05	2.76E+00	2.73E+03	1.09E-01	1.49E-04	3.22E-01	4.08E-06	2.31E-02
	Gamma S	N.D.	N.D.	N.D.	N.D.	N.D.	N.D.	N.D.	N.D.	N.D.	N.D.
	Kappa S	*3.43E-09*[a]	*2.09E-09*								
	Delta S	1.71E-09	2.10E-11	2.44E+05	3.22E+00	1.58E+03	5.29E+00	4.19E-04	6.43E-01	4.35E-06	1.05E+00
**C12A2 (RBD-2)**	G614 S	9.00E-10	1.69E-11	2.01E+05	1.52E-01	1.94E+03	1.42E-02	1.81E-04	2.27E-02	2.93E-06	2.18E-03
	Gamma S	N.D.	N.D.	N.D.	N.D.	N.D.	N.D.	N.D.	N.D.	N.D.	N.D.
	Kappa S	*1.12E-08*	*1.33E-09*								
	Delta S	1.14E-09	1.65E-11	1.69E+05	1.10E-01	1.43E+03	1.39E-02	1.92E-04	2.73E-02	2.26E-06	3.61E-03
**C63C7 (RBD-3)**	G614 S	4.64E-09	1.97E-11	9.09E+04		3.04E+02		4.22E-04		1.10E-06	
	Gamma S	2.86E-09	1.08E-11	1.65E+05		4.44E+02		4.72E-04		1.25E-06	
	Kappa S	1.18E-10	9.51E-12	1.04E+05		3.06E+02		1.23E-05		9.87E-07	
	Delta S	2.36E-09	7.20E-12	1.94E+05		4.01E+02		4.58E-04		1.03E-06	
**C63D3 (RBD-3)**	G614 S	6.87E-09	4.45E-11	2.02E+05		1.19E+03		1.39E-03		3.75E-06	
	Gamma S	6.29E-09	3.40E-11	2.81E+05		1.41E+03		1.77E-03		3.61E-06	
	Kappa S	*1.84E-08*	*1.07E-08*								
	Delta S	6.82E-09	3.79E-11	1.63E+05		8.10E+02		1.11E-03		2.80E-06	
**C12C9 (NTD-1)**	G614 S	3.50E-09	1.91E-11	7.88E+04		2.94E+02		2.75E-04		1.10E-06	
	Gamma S	*1.10E-08*	*6.48E-09*								
	Kappa S	*3.31E-09*	*6.32E-10*								
	Delta S	N.D.	N.D.	N.D.		N.D.		N.D.		N.D.	
**C83B6 (NTD-1)**	G614 S	1.07E-09	5.27E-12	1.86E+05		3.60E+02		1.99E-04		9.03E-07	
	Gamma S	N.D.	N.D.	N.D.		N.D.		N.D.		N.D.	
	Kappa S	*~1.13E-07*	*N.A.*								
	Delta S	N.D.	N.D.	N.D.		N.D.		N.D.		N.D.	
**C81D6 (NTD-2)**	G614 S	3.99E-09	4.74E-11	7.25E+04		6.25E+02		2.89E-04		2.36E-06	
	Gamma S	1.91E-09	5.92E-11	4.84E+04		6.81E+02		9.25E-05		2.56E-06	
	Kappa S	*4.16E-09*	*1.49E-09*								
	Delta S	6.87E-10	2.25E-11	7.02E+04		4.30E+02		4.83E-05		1.56E-06	
**C163E6 (S2-2)**	G614 S	*6.47E-09*	*2.38E-09*								
	Gamma S	*2.04E-08*	*1.37E-08*								
	Kappa S	*3.49E-08*	*5.11E-09*								
	Delta S	*1.03E-08*	*1.75E-09*								

[a]All K_D values for multivalent interactions with antibody IgG or dimeric ACE2 and trimeric S protein are the apparent affinities with avidity effects. [b]Values in italic are derived from steady-state fitting.

Table S2. Neutralization of the SARS-CoV-2 variants

Antibody/ACE2 construct	Neutralization titer (μg/ml)							
	B.1 (G614)		Gamma		Kappa		Delta	
	IC_{50}	IC_{80}	IC_{50}	IC_{80}	IC_{50}	IC_{80}	IC_{50}	IC_{80}
C63C8 (RBD-1)	8.030	27.794	4.378	24.127	5.619	25.555	4.563	19.939
C12A2 (RBD-2)	0.014	0.071	>50	>50	>50	>50	0.063	0.339
G32B6 (RBD-2)	0.018	0.051	>50	>50	>50	>50	0.015	0.048
C63C7 (RBD-3)	4.729	29.985	3.273	18.781	4.532	20.257	7.704	34.343
C63D3 (RBD-3)	37.186	>50	30.614	>50	>50	>50	46.126	>50
C12C9 (NTD-1)	0.019	43.34	0.341	>50	22.939	>50	>50	>50
C83B6 (NTD-1)	0.105	>50	>50	>50	>50	>50	>50	>50
C81D6 (NTD-2)	>50	>50	>50	>50	>50	>50	>50	>50
C163E6 (S2-2)	>50	>50	>50	>50	>50	>50	>50	>50
ACE2-T27W-Fd	0.079	0.568	0.036	0.180	0.045	0.147	0.022	0.054
Positive serum pool_2 (1/x)	1,922	426	58	23	1,046	96	2,300	150
Normal Human Serum (1/x)	<20	<20	<20	<20	<20	<20	<20	<20

Table S3. Cryo-EM statistics.

EM data collection and reconstruction statistics

Protein	Full-length S of Delta variant			Full-length S of Gamma variant		Full-length S of Kappa variant		
Microscope	Titan Krios			Titan Krios		Titan Krios		
Voltage(kV)	300			300		300		
Detector	Gatan K3			Gatan K3		Gatan K3		
Magnification(nominal)	105,000			105,000		105,000		
Energy filter slit width (eV)	20			20		20		
Calibrated pixel size (Å/pix)	0.825			0.825		0.825		
Exposure rate (e^-/pix/sec)	20.24			20.69/20.63/27.13		21.12/20,101		
Frames per exposure	50			51/51/50		51/51		
Total electron exposure (e^-/Å^2)	51.48			54.72/54.56/53.4		51.63/51.151		
Exposure per frame (e^-/Å^2)	1.03			1.073/1.07/1.06		1.012/1.003		
Defocus range (µm)	-0.8,-2.2			-0.8, -2.3		-0.8, -2.2		
Automation software	SerialEM			SerialEM		SerialEM		
# of Micrographs used	20,274			25,424/32,569/29,163		22,019/17,314		
Particles extracted	1,830,328			1,652,420/2,757,190/1,893,863		1,199,999/1,718,600		
Particles after 2D classification	1,386,630			1,564,938/1,904,078/1,788,000		1,112,384/1,649,296		
Class	Closed	RBD-up 1	RBD-up 2	RBD-up 1	RBD-up 2	Closed	RBD-up 1	RBD-up 2
Total # of refined particles	94,680	191,067	25,370	69,302	36,346	123,193	81,717	21,830
Symmetry imposed	C3	C1	C1	C1	C1	C3	C1	C1
Estimated accuracy of translations/rotations	0.87/1.96	1.23/2.45	2.35/4.24	1.73/3.11	2.27/3.80	1.04/2.29	1.66/3.24	1.86/3.36
Map sharpening B-factor	-93.6	-111.9	-92.1	-124.6	-153.7	-103.6	-121.8	-130.3
Unmasked Resolution at 0.5/0.143 FSC (Å)	3.9/3.4	4.4/3.8	8.9/7.3	7.1/4.3	8.4/6.1	4.0/3.6	6.4/4.0	8.5/5.9
Masked resolution at 0.5/0.143 FSC (Å)	3.6/3.1	3.9/3.4	7.3/4.3	4.5/3.8	6.9/4.4	3.6/3.1	4.2/3.7	7.4/4.3

Model refinement and validation statistics

Class	Closed	RBD-up 1	RBD-up 2	RBD-up 1	RBD-up 2	Closed	RBD-up 1	RBD-up 2
PDB								
Composition								
Amino acids	3303	3287	3269	3230	3300	3330	3270	3270
Glycans	57	57	57	60	60	57	57	57
RMSD bonds (Å)	0.014	0.014	0.015	0.013	0.013	0.013	0.015	0.015
RMSD angles (◦)	1.80	1.91	1.92	1.81	1.83	1.88	2.08	1.88
Mean B-factors								
Amino acids	96	64	64	62	62	105	128	64
Glycans	127	88	88	88	88	137	159	87
Ramachandran								
Favored (%)	93.00	91.80	91.68	92.10	91.96	92.85	92.61	91.58
Allowed(%)	6.23	7.43	7.42	7.17	7.16	6.55	6.96	7.77
Outliers(%)	0.76	0.77	0.90	0.73	0.89	0.61	0.43	0.65
Rotamer outliers (%)	1.39	2.97	3.86	2.10	1.84	1.17	1.82	2.38
Clash score	0.35	2.26	3.42	2.28	3.43	0.46	0.77	2.67
C-beta outliers (%)	0.42	0.52	1.01	0.51	0.49	0.45	0.52	0.39
CaBLAM outliers (%)	3.11	3.57	3.57	2.68	2.99	3.18	3.01	3.07
CC (mask)	0.80	0.76	0.61	0.69	0.63	0.81	0.79	0.63
CC (volume)	0.80	0.76	0.60	0.69	0.63	0.80	0.78	0.62
MolProbity score	1.18	1.86	2.08	1.73	1.82	1.17	1.41	1.84
EMRinger score	3.73	2.68	0.88	1.74	0.74	3.16	2.46	0.73

References and Notes

1. P. Zhou, X.-L. Yang, X.-G. Wang, B. Hu, L. Zhang, W. Zhang, H.-R. Si, Y. Zhu, B. Li, C.-L. Huang, H.-D. Chen, J. Chen, Y. Luo, H. Guo, R.-D. Jiang, M.-Q. Liu, Y. Chen, X.-R. Shen, X. Wang, X.-S. Zheng, K. Zhao, Q.-J. Chen, F. Deng, L.-L. Liu, B. Yan, F.-X. Zhan, Y.-Y. Wang, G.-F. Xiao, Z.-L. Shi, A pneumonia outbreak associated with a new coronavirus of probable bat origin. *Nature* **579**, 270–273 (2020). doi:10.1038/s41586-020-2012-7 Medline

2. Y. Cai, J. Zhang, T. Xiao, C. L. Lavine, S. Rawson, H. Peng, H. Zhu, K. Anand, P. Tong, A. Gautam, S. Lu, S. M. Sterling, R. M. Walsh Jr., S. Rits-Volloch, J. Lu, D. R. Wesemann, W. Yang, M. S. Seaman, B. Chen, Structural basis for enhanced infectivity and immune evasion of SARS-CoV-2 variants. *Science* **373**, 642–648 (2021). doi:10.1126/science.abi9745 Medline

3. P. Mlcochova, S. A. Kemp, M. S. Dhar, G. Papa, B. Meng, I. A. T. M. Ferreira, R. Datir, D. A. Collier, A. Albecka, S. Singh, R. Pandey, J. Brown, J. Zhou, N. Goonawardane, S. Mishra, C. Whittaker, T. Mellan, R. Marwal, M. Datta, S. Sengupta, K. Ponnusamy, V. S. Radhakrishnan, A. Abdullahi, O. Charles, P. Chattopadhyay, P. Devi, D. Caputo, T. Peacock, C. Wattal, N. Goel, A. Satwik, R. Vaishya, M. Agarwal, The Indian SARS-CoV-2 Genomics Consortium (INSACOG), The Genotype to Phenotype Japan (G2P-Japan) Consortium, The CITIID-NIHR BioResource COVID-19 Collaboration, A. Mavousian, J. Hyeon Lee, J. Bassi, C. Silacci-Fegni, C. Saliba, D.Pinto, T. Irie, I. Yoshida, W. L. Hamilton, K. Sato, S. Bhatt, S. Flaxman, L. C. James, D. Corti, L. Piccoli, W. S. Barclay, P. Rakshit, A. Agrawal, and R. K. Gupta, Delta variant replication and immune evasion. *Nature* **599**, 114–119 (2021). doi:10.1038/s41586-021-03944-y Medline

4. U. K. G. Scientific Advisory Group for Emergencies (SAGE), SPI-M-O: Consensus Statement on COVID-19. https://assets.publishing.service.gov.uk/government/uploads/system/uploads/attachment_data/file/993321/S991267_SPI-M-O_Consensus_Statement.pdf (2021).

5. J. Dagpunar, Interim estimates of increased transmissibility, growth rate, and reproduction number of the Covid-19 B.1.617.2 variant of concern in the United Kingdom. *medRxiv*, 2021.2006.2003.21258293 (2021).

6. B. Li *et al.*, Viral infection and transmission in a large well-traced outbreak caused by the Delta SARS-CoV-2 variant. *medRxiv*, https://doi.org/10.1101/2021.1107.1107.21260122 (2021).

7. A. Sheikh, J. McMenamin, B. Taylor, C. Robertson; Public Health Scotland and the EAVE II Collaborators, SARS-CoV-2 Delta VOC in Scotland: Demographics, risk of hospital admission, and vaccine effectiveness. *Lancet* **397**, 2461–2462 (2021). doi:10.1016/S0140-6736(21)01358-1 Medline

8. D. N. Fisman, A. R. Tuite, Evaluation of the relative virulence of novel SARS-CoV-2 variants: a retrospective cohort study in Ontario, Canada. *CMAJ* **42**, E1619–E1625 (2021). doi:10.1503/cmaj.211248 Medline

9. D. Planas, D. Veyer, A. Baidaliuk, I. Staropoli, F. Guivel-Benhassine, M. M. Rajah, C. Planchais, F. Porrot, N. Robillard, J. Puech, M. Prot, F. Gallais, P. Gantner, A. Velay, J. Le Guen, N. Kassis-Chikhani, D. Edriss, L. Belec, A. Seve, L. Courtellemont, H. Péré, L. Hocqueloux, S. Fafi-Kremer, T. Prazuck, H. Mouquet, T. Bruel, E. Simon-Lorière, F. A. Rey, O. Schwartz, Reduced sensitivity of SARS-CoV-2 variant Delta to antibody neutralization. *Nature* **596**, 276–280 (2021). doi:10.1038/s41586-021-03777-9 Medline

10. V. V. Edara, B. A. Pinsky, M. S. Suthar, L. Lai, M. E. Davis-Gardner, K. Floyd, M. W. Flowers, J. Wrammert, L. Hussaini, C. R. Ciric, S. Bechnak, K. Stephens, B. S. Graham, E. Bayat Mokhtari, P. Mudvari, E. Boritz, A. Creanga, A. Pegu, A. Derrien-Colemyn, A. R. Henry, M. Gagne, D. C. Douek, M. K. Sahoo, M. Sibai, D. Solis, R. J. Webby, T. Jeevan, T. P. Fabrizio, Infection and Vaccine-Induced Neutralizing-Antibody Responses to the SARS-CoV-2 B.1.617 Variants. *N. Engl. J. Med.* **385**, 664–666 (2021). doi:10.1056/NEJMc2107799 Medline

11. J. Lopez Bernal, N. Andrews, C. Gower, E. Gallagher, R. Simmons, S. Thelwall, J. Stowe, E. Tessier, N. Groves, G. Dabrera, R. Myers, C. N. J. Campbell, G. Amirthalingam, M. Edmunds, M. Zambon, K. E. Brown, S. Hopkins, M. Chand, M. Ramsay, Effectiveness of Covid-19 Vaccines against the B.1.617.2 (Delta) Variant. *N. Engl. J. Med.* **385**, 585–594 (2021). doi:10.1056/NEJMoa2108891 Medline

12. T. Tada *et al.*, Comparison of Neutralizing Antibody Titers Elicited by mRNA and Adenoviral Vector Vaccine against SARS-CoV-2 Variants. *bioRxiv*, 2021.2007.2019.452771 (2021).

13. N. R. Faria, T. A. Mellan, C. Whittaker, I. M. Claro, D. D. S. Candido, S. Mishra, M. A. E. Crispim, F. C. S. Sales, I. Hawryluk, J. T. McCrone, R. J. G. Hulswit, L. A. M. Franco, M. S. Ramundo, J. G. de Jesus, P. S. Andrade, T. M. Coletti, G. M. Ferreira, C. A. M. Silva, E. R. Manuli, R. H. M. Pereira, P. S. Peixoto, M. U. G. Kraemer, N. Gaburo Jr., C. D. C. Camilo, H. Hoeltgebaum, W. M. Souza, E. C. Rocha, L. M. de Souza, M. C. de Pinho, L. J. T. Araujo, F. S. V. Malta, A. B. de Lima, J. D. P. Silva, D. A. G. Zauli, A. C. S. Ferreira, R. P. Schnekenberg, D. J. Laydon, P. G. T. Walker, H. M. Schlüter, A. L. P. Dos Santos, M. S. Vidal, V. S. Del Caro, R. M. F. Filho, H. M. Dos Santos, R. S. Aguiar, J. L. Proença-Modena, B. Nelson, J. A. Hay, M. Monod, X. Miscouridou, H. Coupland, R. Sonabend, M. Vollmer, A. Gandy, C. A. Prete Jr., V. H. Nascimento, M. A. Suchard, T. A. Bowden, S. L. K. Pond, C.-H. Wu, O. Ratmann, N. M. Ferguson, C. Dye, N. J. Loman, P. Lemey, A. Rambaut, N. A. Fraiji, M. D. P. S. S. Carvalho, O. G. Pybus, S. Flaxman, S. Bhatt, E. C. Sabino, Genomics and epidemiology of the P.1 SARS-CoV-2 lineage in Manaus, Brazil. *Science* **372**, 815–821 (2021). doi:10.1126/science.abh2644 Medline

14. M. Hoffmann, P. Arora, R. Groß, A. Seidel, B. F. Hörnich, A. S. Hahn, N. Krüger, L. Graichen, H. Hofmann-Winkler, A. Kempf, M. S. Winkler, S. Schulz, H.-M. Jäck, B. Jahrsdörfer, H. Schrezenmeier, M. Müller, A. Kleger, J. Münch, S. Pöhlmann, SARS-CoV-2 variants B.1.351 and P.1 escape from neutralizing antibodies. *Cell* **184**, 2384–2393.e12 (2021). doi:10.1016/j.cell.2021.03.036 Medline

15. P. D. Yadav *et al*., SARS CoV-2 variant B.1.617.1 is highly pathogenic in hamsters than B.1 variant. *bioRxiv*, 2021.2005.2005.442760 (2021).

16. V.-V. Edara, L. Lai, M. K. Sahoo, K. Floyd, M. Sibai, D. Solis, M. W. Flowers, L. Hussaini, C. R. Ciric, S. Bechnack, K. Stephens, E. B. Mokhtari, P. Mudvari, A. Creanga, A. Pegu, A. Derrien-Colemyn, A. R. Henry, M. Gagne, B. S. Graham, J. Wrammert, D. C. Douek, E. Boritz, B. A. Pinsky, M. S. Suthar, Infection and Vaccine-Induced Neutralizing-Antibody Responses to the SARS-CoV-2 B.1.617 Variants. *N. Engl. J. Med.* **385**, 664–666 (2021). doi:10.1056/NEJMc2107799 Medline

17. B. J. Bosch, R. van der Zee, C. A. de Haan, P. J. Rottier, The coronavirus spike protein is a class I virus fusion protein: Structural and functional characterization of the fusion core complex. *J. Virol.* **77**, 8801–8811 (2003). doi:10.1128/JVI.77.16.8801-8811.2003 Medline

18. M. Hoffmann, H. Kleine-Weber, S. Schroeder, N. Krüger, T. Herrler, S. Erichsen, T. S. Schiergens, G. Herrler, N.-H. Wu, A. Nitsche, M. A. Müller, C. Drosten, S. Pöhlmann, SARS-CoV-2 Cell Entry Depends on ACE2 and TMPRSS2 and Is Blocked by a Clinically Proven Protease Inhibitor. *Cell* **181**, 271–280.e8 (2020). doi:10.1016/j.cell.2020.02.052 Medline

19. J. K. Millet, G. R. Whittaker, Host cell entry of Middle East respiratory syndrome coronavirus after two-step, furin-mediated activation of the spike protein. *Proc. Natl. Acad. Sci. U.S.A.* **111**, 15214–15219 (2014). doi:10.1073/pnas.1407087111 Medline

20. M. A. Tortorici, D. Veesler, Structural insights into coronavirus entry. *Adv. Virus Res.* **105**, 93–116 (2019). doi:10.1016/bs.aivir.2019.08.002 Medline

21. D. Wrapp, N. Wang, K. S. Corbett, J. A. Goldsmith, C.-L. Hsieh, O. Abiona, B. S. Graham, J. S. McLellan, Cryo-EM structure of the 2019-nCoV spike in the prefusion conformation. *Science* **367**, 1260–1263 (2020). doi:10.1126/science.abb2507 Medline

22. J. Shang, Y. Wan, C. Luo, G. Ye, Q. Geng, A. Auerbach, F. Li, Cell entry mechanisms of SARS-CoV-2. *Proc. Natl. Acad. Sci. U.S.A.* **117**, 11727–11734 (2020). doi:10.1073/pnas.2003138117 Medline

23. Y. Huang, C. Yang, X. F. Xu, W. Xu, S. W. Liu, Structural and functional properties of SARS-CoV-2 spike protein: Potential antivirus drug development for COVID-19. *Acta Pharmacol. Sin.* **41**, 1141–1149 (2020). doi:10.1038/s41401-020-0485-4 Medline

24. C. Zhu, G. He, Q. Yin, L. Zeng, X. Ye, Y. Shi, W. Xu, Molecular biology of the SARs-CoV-2 spike protein: A review of current knowledge. *J. Med. Virol.* **93**, 5729–5741 (2021). doi:10.1002/jmv.27132 Medline

25. N. Murgolo, A. G. Therien, B. Howell, D. Klein, K. Koeplinger, L. A. Lieberman, G. C. Adam, J. Flynn, P. McKenna, G. Swaminathan, D. J. Hazuda, D. B. Olsen, SARS-CoV-2 tropism, entry, replication, and propagation: Considerations for

drug discovery and development. *PLOS Pathog.* **17**, e1009225 (2021). doi:10.1371/journal.ppat.1009225 Medline

26. R. Peng, L. A. Wu, Q. Wang, J. Qi, G. F. Gao, Cell entry by SARS-CoV-2. *Trends Biochem. Sci.* **46**, 848–860 (2021). doi:10.1016/j.tibs.2021.06.001 Medline
27. B. Korber, W. M. Fischer, S. Gnanakaran, H. Yoon, J. Theiler, W. Abfalterer, N. Hengartner, E. E. Giorgi, T. Bhattacharya, B. Foley, K. M. Hastie, M. D. Parker, D. G. Partridge, C. M. Evans, T. M. Freeman, T. I. de Silva, C. McDanal, L. G. Perez, H. Tang, A. Moon-Walker, S. P. Whelan, C. C. LaBranche, E. O. Saphire, D. C. Montefiori, A. Angyal, R. L. Brown, L. Carrilero, L. R. Green, D. C. Groves, K. J. Johnson, A. J. Keeley, B. B. Lindsey, P. J. Parsons, M. Raza, S. Rowland-Jones, N. Smith, R. M. Tucker, D. Wang, M. D. Wyles; Sheffield COVID-19 Genomics Group, Tracking Changes in SARS-CoV-2 Spike: Evidence that D614G Increases Infectivity of the COVID-19 Virus. *Cell* **182**, 812–827.e19 (2020). doi:10.1016/j.cell.2020.06.043 Medline
28. Y. Cai, J. Zhang, T. Xiao, H. Peng, S. M. Sterling, R. M. Walsh Jr., S. Rawson, S. Rits-Volloch, B. Chen, Distinct conformational states of SARS-CoV-2 spike protein. *Science* **369**, 1586–1592 (2020). doi:10.1126/science.abd4251 Medline
29. J. Yu, Z. Li, X. He, M. S. Gebre, E. A. Bondzie, H. Wan, C. Jacob-Dolan, D. R. Martinez, J. P. Nkolola, R. S. Baric, D. H. Barouch, Deletion of the SARS-CoV-2 Spike Cytoplasmic Tail Increases Infectivity in Pseudovirus Neutralization Assays. *J. Virol.* **95**, JVI.00044-21 (2021). doi:10.1128/JVI.00044-21 Medline
30. T. Giroglou, J. Cinatl Jr., H. Rabenau, C. Drosten, H. Schwalbe, H. W. Doerr, D. von Laer, Retroviral vectors pseudotyped with severe acute respiratory syndrome coronavirus S protein. *J. Virol.* **78**, 9007–9015 (2004). doi:10.1128/JVI.78.17.9007-9015.2004 Medline
31. J. Zhang, Y. Cai, T. Xiao, J. Lu, H. Peng, S. M. Sterling, R. M. Walsh Jr., S. Rits-Volloch, H. Zhu, A. N. Woosley, W. Yang, P. Sliz, B. Chen, Structural impact on SARS-CoV-2 spike protein by D614G substitution. *Science* **372**, 525–530 (2021). doi:10.1126/science.abf2303 Medline
32. P. Tong, A. Gautam, I. W. Windsor, M. Travers, Y. Chen, N. Garcia, N. B. Whiteman, L. G. A. McKay, N. Storm, L. E. Malsick, A. N. Honko, F. J. N. Lelis, S. Habibi, S. Jenni, Y. Cai, L. J. Rennick, W. P. Duprex, K. R. McCarthy, C. L. Lavine, T. Zuo, J. Lin, A. Zuiani, J. Feldman, E. A. MacDonald, B. M. Hauser, A. Griffths, M. S. Seaman, A. G. Schmidt, B. Chen, D. Neuberg, G. Bajic, S. C. Harrison, D. R. Wesemann, Memory B cell repertoire for recognition of evolving SARS-CoV-2 spike. *Cell* **184**, 4969–4980.e15 (2021). doi:10.1016/j.cell.2021.07.025 Medline
33. T. Xiao, J. Lu, J. Zhang, R. I. Johnson, L. G. A. McKay, N. Storm, C. L. Lavine, H. Peng, Y. Cai, S. Rits-Volloch, S. Lu, B. D. Quinlan, M. Farzan, M. S. Seaman, A. Griffiths, B. Chen, A trimeric human angiotensin-converting enzyme 2 as an anti-SARS-CoV-2 agent. *Nat. Struct. Mol. Biol.* **28**, 202–209 (2021). doi:10.1038/s41594-020-00549-3 Medline

34. T. Sztain, S.-H. Ahn, A. T. Bogetti, L. Casalino, J. A. Goldsmith, E. Seitz, R. S. McCool, F. L. Kearns, F. Acosta-Reyes, S. Maji, G. Mashayekhi, J. A. McCammon, A. Ourmazd, J. Frank, J. S. McLellan, L. T. Chong, R. E. Amaro, A glycan gate controls opening of the SARS-CoV-2 spike protein. *Nat. Chem.* **13**, 963–968 (2021). doi:10.1038/s41557-021-00758-3 Medline

35. G. Cerutti, Y. Guo, T. Zhou, J. Gorman, M. Lee, M. Rapp, E. R. Reddem, J. Yu, F. Bahna, J. Bimela, Y. Huang, P. S. Katsamba, L. Liu, M. S. Nair, R. Rawi, A. S. Olia, P. Wang, B. Zhang, G.-Y. Chuang, D. D. Ho, Z. Sheng, P. D. Kwong, L. Shapiro, Potent SARS-CoV-2 neutralizing antibodies directed against spike N-terminal domain target a single supersite. *Cell Host Microbe* **29**, 819–833.e7 (2021). doi:10.1016/j.chom.2021.03.005 Medline

36. X. Chi, R. Yan, J. Zhang, G. Zhang, Y. Zhang, M. Hao, Z. Zhang, P. Fan, Y. Dong, Y. Yang, Z. Chen, Y. Guo, J. Zhang, Y. Li, X. Song, Y. Chen, L. Xia, L. Fu, L. Hou, J. Xu, C. Yu, J. Li, Q. Zhou, W. Chen, A neutralizing human antibody binds to the N-terminal domain of the Spike protein of SARS-CoV-2. *Science* **369**, 650–655 (2020). doi:10.1126/science.abc6952 Medline

37. M. McCallum, A. De Marco, F. A. Lempp, M. A. Tortorici, D. Pinto, A. C. Walls, M. Beltramello, A. Chen, Z. Liu, F. Zatta, S. Zepeda, J. di Iulio, J. E. Bowen, M. Montiel-Ruiz, J. Zhou, L. E. Rosen, S. Bianchi, B. Guarino, C. S. Fregni, R. Abdelnabi, S. C. Foo, P. W. Rothlauf, L.-M. Bloyet, F. Benigni, E. Cameroni, J. Neyts, A. Riva, G. Snell, A. Telenti, S. P. J. Whelan, H. W. Virgin, D. Corti, M. S. Pizzuto, D. Veesler, N-terminal domain antigenic mapping reveals a site of vulnerability for SARS-CoV-2. *Cell* **184**, 2332–2347.e16 (2021). doi:10.1016/j.cell.2021.03.028 Medline

38. D. Li, R. J. Edwards, K. Manne, D. R. Martinez, A. Schäfer, S. M. Alam, K. Wiehe, X. Lu, R. Parks, L. L. Sutherland, T. H. Oguin 3rd, C. McDanal, L. G. Perez, K. Mansouri, S. M. C. Gobeil, K. Janowska, V. Stalls, M. Kopp, F. Cai, E. Lee, A. Foulger, G. E. Hernandez, A. Sanzone, K. Tilahun, C. Jiang, L. V. Tse, K. W. Bock, M. Minai, B. M. Nagata, K. Cronin, V. Gee-Lai, M. Deyton, M. Barr, T. Von Holle, A. N. Macintyre, E. Stover, J. Feldman, B. M. Hauser, T. M. Caradonna, T. D. Scobey, W. Rountree, Y. Wang, M. A. Moody, D. W. Cain, C. T. DeMarco, T. N. Denny, C. W. Woods, E. W. Petzold, A. G. Schmidt, I.-T. Teng, T. Zhou, P. D. Kwong, J. R. Mascola, B. S. Graham, I. N. Moore, R. Seder, H. Andersen, M. G. Lewis, D. C. Montefiori, G. D. Sempowski, R. S. Baric, P. Acharya, B. F. Haynes, K. O. Saunders, In vitro and in vivo functions of SARS-CoV-2 infection-enhancing and neutralizing antibodies. *Cell* **184**, 4203–4219.e32 (2021). doi:10.1016/j.cell.2021.06.021 Medline

39. M. McCallum, A. C. Walls, K. R. Sprouse, J. E. Bowen, L. E. Rosen, H. V. Dang, Anna. D. Marco, N. Franko, S.W. Tilles, J. Logue, M. C. Miranda, M. Ahlrichs, L. Carter, G. Snell, M. S. Pizzuto, H. Y. Chu, W. C. V. Voorhis, D. Corti, D. Veesler, Molecular basis of immune evasion by the Delta and Kappa SARS-CoV-2 variants. *Science*, eabl8506 (2021). doi:10.1126/science.abl8506

40. J. Lan, J. Ge, J. Yu, S. Shan, H. Zhou, S. Fan, Q. Zhang, X. Shi, Q. Wang, L. Zhang, X. Wang, Structure of the SARS-CoV-2 spike receptor-binding domain bound to

the ACE2 receptor. *Nature* **581**, 215–220 (2020). doi:10.1038/s41586-020-2180-5 Medline

41. J. Shang, G. Ye, K. Shi, Y. Wan, C. Luo, H. Aihara, Q. Geng, A. Auerbach, F. Li, Structural basis of receptor recognition by SARS-CoV-2. *Nature* **581**, 221–224 (2020). doi:10.1038/s41586-020-2179-y Medline

42. A. C. Walls, Y.-J. Park, M. A. Tortorici, A. Wall, A. T. McGuire, D. Veesler, Structure, Function, and Antigenicity of the SARS-CoV-2 Spike Glycoprotein. *Cell* **183**, 1735 (2020). doi:10.1016/j.cell.2020.11.032 Medline

43. S. M. Gobeil, K. Janowska, S. McDowell, K. Mansouri, R. Parks, V. Stalls, M. F. Kopp, K. Manne, D. Li, K. Wiehe, K. O. Saunders, R. J. Edwards, B. Korber, B. F. Haynes, R. Henderson, P. Acharya, Effect of natural mutations of SARS-CoV-2 on spike structure, conformation, and antigenicity. *Science* **373**, eabi6226 (2021). doi:10.1126/science.abi6226 Medline

44. T. N. Starr, A. J. Greaney, S. K. Hilton, D. Ellis, K. H. D. Crawford, A. S. Dingens, M. J. Navarro, J. E. Bowen, M. A. Tortorici, A. C. Walls, N. P. King, D. Veesler, J. D. Bloom, Deep Mutational Scanning of SARS-CoV-2 Receptor Binding Domain Reveals Constraints on Folding and ACE2 Binding. *Cell* **182**, 1295–1310.e20 (2020). doi:10.1016/j.cell.2020.08.012 Medline

45. M. Yuan, D. Huang, C. D. Lee, N. C. Wu, A. M. Jackson, X. Zhu, H. Liu, L. Peng, M. J. van Gils, R. W. Sanders, D. R. Burton, S. M. Reincke, H. Prüss, J. Kreye, D. Nemazee, A. B. Ward, I. A. Wilson, Structural and functional ramifications of antigenic drift in recent SARS-CoV-2 variants. *Science* **373**, 818–823 (2021). doi:10.1126/science.abh1139 Medline

46. T. J. Yang, P.-Y. Yu, Y.-C. Chang, K.-H. Liang, H.-C. Tso, M.-R. Ho, W.-Y. Chen, H.-T. Lin, H.-C. Wu, S. D. Hsu, Effect of SARS-CoV-2 B.1.1.7 mutations on spike protein structure and function. *Nat. Struct. Mol. Biol.* **28**, 731–739 (2021). doi:10.1038/s41594-021-00652-z Medline

47. M. Ferrari, L. Mekkaoui, F. T. Ilca, Z. Akbar, R. Bughda, K. Lamb, K. Ward, F. Parekh, R. Karattil, C. Allen, P. Wu, V. Baldan, G. Mattiuzzo, E. M. Bentley, Y. Takeuchi, J. Sillibourne, P. Datta, A. Kinna, M. Pule, S. C. Onuoha, Characterization of a Novel ACE2-Based Therapeutic with Enhanced Rather than Reduced Activity against SARS-CoV-2 Variants. *J. Virol.* **95**, e0068521 (2021). doi:10.1128/JVI.00685-21 Medline

48. P. Wang, M. S. Nair, L. Liu, S. Iketani, Y. Luo, Y. Guo, M. Wang, J. Yu, B. Zhang, P. D. Kwong, B. S. Graham, J. R. Mascola, J. Y. Chang, M. T. Yin, M. Sobieszczyk, C. A. Kyratsous, L. Shapiro, Z. Sheng, Y. Huang, D. D. Ho, Antibody resistance of SARS-CoV-2 variants B.1.351 and B.1.1.7. *Nature* **593**, 130–135 (2021). doi:10.1038/s41586-021-03398-2 Medline

49. E. Volz, S. Mishra, M. Chand, J. C. Barrett, R. Johnson, L. Geidelberg, W. R. Hinsley, D. J. Laydon, G. Dabrera, Á. O'Toole, R. Amato, M. Ragonnet-Cronin, I. Harrison, B. Jackson, C. V. Ariani, O. Boyd, N. J. Loman, J. T. McCrone, S. Gonçalves, D. Jorgensen, R. Myers, V. Hill, D. K. Jackson, K. Gaythorpe, N. Groves, J. Sillitoe, D. P. Kwiatkowski, S. Flaxman, O. Ratmann, S. Bhatt, S.

Hopkins, A. Gandy, A. Rambaut, N. M. Ferguson; COVID-19 Genomics UK (COG-UK) consortium, Assessing transmissibility of SARS-CoV-2 lineage B.1.1.7 in England. *Nature* **593**, 266–269 (2021). doi:10.1038/s41586-021-03470-x Medline

50. M. U. G. Kraemer, V. Hill, C. Ruis, S. Dellicour, S. Bajaj, J. T. McCrone, G. Baele, K. V. Parag, A. L. Battle, B. Gutierrez, B. Jackson, R. Colquhoun, Á. O'Toole, B. Klein, A. Vespignani, E. Volz, N. R. Faria, D. M. Aanensen, N. J. Loman, L. du Plessis, S. Cauchemez, A. Rambaut, S. V. Scarpino, O. G. Pybus; COVID-19 Genomics UK (COG-UK) Consortium, Spatiotemporal invasion dynamics of SARS-CoV-2 lineage B.1.1.7 emergence. *Science* **373**, 889–895 (2021). doi:10.1126/science.abj0113 Medline
51. N. G. Davies, S. Abbott, R. C. Barnard, C. I. Jarvis, A. J. Kucharski, J. D. Munday, C. A. B. Pearson, T. W. Russell, D. C. Tully, A. D. Washburne, T. Wenseleers, A. Gimma, W. Waites, K. L. M. Wong, K. van Zandvoort, J. D. Silverman, K. Diaz-Ordaz, R. Keogh, R. M. Eggo, S. Funk, M. Jit, K. E. Atkins, W. J. Edmunds; CMMID COVID-19 Working Group; COVID-19 Genomics UK (COG-UK) Consortium, Estimated transmissibility and impact of SARS-CoV-2 lineage B.1.1.7 in England. *Science* **372**, eabg3055 (2021). doi:10.1126/science.abg3055 Medline
52. S. Kim *et al*., Differential Interactions Between Human ACE2 and Spike RBD of SARS-CoV-2 Variants of Concern. *bioRxiv*, 2021.2007.2023.453598 (2021).
53. A. Saito *et al*., SARS-CoV-2 spike P681R mutation enhances and accelerates viral fusion. *bioRxiv*, 2021.2006.2017.448820 (2021).
54. Y. Liu *et al*., Delta spike P681R mutation enhances SARS-CoV-2 fitness over Alpha variant. *bioRxiv*, 2021.2008.2012.456173 (2021).
55. L. Liu, P. Wang, M. S. Nair, J. Yu, M. Rapp, Q. Wang, Y. Luo, J. F.-W. Chan, V. Sahi, A. Figueroa, X. V. Guo, G. Cerutti, J. Bimela, J. Gorman, T. Zhou, Z. Chen, K.-Y. Yuen, P. D. Kwong, J. G. Sodroski, M. T. Yin, Z. Sheng, Y. Huang, L. Shapiro, D. D. Ho, Potent neutralizing antibodies against multiple epitopes on SARS-CoV-2 spike. *Nature* **584**, 450–456 (2020). doi:10.1038/s41586-020-2571-7 Medline
56. C. O. Barnes, A. P. West Jr., K. E. Huey-Tubman, M. A. G. Hoffmann, N. G. Sharaf, P. R. Hoffman, N. Koranda, H. B. Gristick, C. Gaebler, F. Muecksch, J. C. C. Lorenzi, S. Finkin, T. Hägglöf, A. Hurley, K. G. Millard, Y. Weisblum, F. Schmidt, T. Hatziioannou, P. D. Bieniasz, M. Caskey, D. F. Robbiani, M. C. Nussenzweig, P. J. Bjorkman, Structures of Human Antibodies Bound to SARS-CoV-2 Spike Reveal Common Epitopes and Recurrent Features of Antibodies. *Cell* **182**, 828–842.e16 (2020). doi:10.1016/j.cell.2020.06.025 Medline
57. J. Chen, J. M. Kovacs, H. Peng, S. Rits-Volloch, J. Lu, D. Park, E. Zablowsky, M. S. Seaman, B. Chen, HIV-1 ENVELOPE. Effect of the cytoplasmic domain on antigenic characteristics of HIV-1 envelope glycoprotein. *Science* **349**, 191–195 (2015). doi:10.1126/science.aaa9804 Medline

58. C. Z. Chen, M. Xu, M. Pradhan, K. Gorshkov, J. D. Petersen, M. R. Straus, W. Zhu, P. Shinn, H. Guo, M. Shen, C. Klumpp-Thomas, S. G. Michael, J. Zimmerberg, W. Zheng, G. R. Whittaker, Identifying SARS-CoV-2 Entry Inhibitors through Drug Repurposing Screens of SARS-S and MERS-S Pseudotyped Particles. *ACS Pharmacol. Transl. Sci.* **3**, 1165–1175 (2020). doi:10.1021/acsptsci.0c00112 Medline

59. J. K. Millet, G. R. Whittaker, Murine Leukemia Virus (MLV)-based Coronavirus Spike-pseudotyped Particle Production and Infection. *Biol. Protoc* **6**, (2016).

60. D. N. Mastronarde, Automated electron microscope tomography using robust prediction of specimen movements. *J. Struct. Biol.* **152**, 36–51 (2005). doi:10.1016/j.jsb.2005.07.007 Medline

61. S. Q. Zheng, E. Palovcak, J.-P. Armache, K. A. Verba, Y. Cheng, D. A. Agard, MotionCor2: Anisotropic correction of beam-induced motion for improved cryo-electron microscopy. *Nat. Methods* **14**, 331–332 (2017). doi:10.1038/nmeth.4193 Medline

62. K. Zhang, Gctf: Real-time CTF determination and correction. *J. Struct. Biol.* **193**, 1–12 (2016). doi:10.1016/j.jsb.2015.11.003 Medline

63. T. Wagner, F. Merino, M. Stabrin, T. Moriya, C. Antoni, A. Apelbaum, P. Hagel, O. Sitsel, T. Raisch, D. Prumbaum, D. Quentin, D. Roderer, S. Tacke, B. Siebolds, E. Schubert, T. R. Shaikh, P. Lill, C. Gatsogiannis, S. Raunser, SPHIRE-crYOLO is a fast and accurate fully automated particle picker for cryo-EM. *Commun. Biol.* **2**, 218 (2019). doi:10.1038/s42003-019-0437-z Medline

64. S. H. Scheres, RELION: Implementation of a Bayesian approach to cryo-EM structure determination. *J. Struct. Biol.* **180**, 519–530 (2012). doi:10.1016/j.jsb.2012.09.006 Medline

65. A. Kucukelbir, F. J. Sigworth, H. D. Tagare, Quantifying the local resolution of cryo-EM density maps. *Nat. Methods* **11**, 63–65 (2014). doi:10.1038/nmeth.2727 Medline

66. P. Emsley, B. Lohkamp, W. G. Scott, K. Cowtan, Features and development of Coot. *Acta Crystallogr. D Biol. Crystallogr.* **66**, 486–501 (2010). doi:10.1107/S0907444910007493 Medline

67. P. D. Adams, P. V. Afonine, G. Bunkóczi, V. B. Chen, I. W. Davis, N. Echols, J. J. Headd, L.-W. Hung, G. J. Kapral, R. W. Grosse-Kunstleve, A. J. McCoy, N. W. Moriarty, R. Oeffner, R. J. Read, D. C. Richardson, J. S. Richardson, T. C. Terwilliger, P. H. Zwart, PHENIX: A comprehensive Python-based system for macromolecular structure solution. *Acta Crystallogr. D Biol. Crystallogr.* **66**, 213–221 (2010). doi:10.1107/S0907444909052925 Medline

68. T. I. Croll, ISOLDE: A physically realistic environment for model building into low-resolution electron-density maps. *Acta Crystallogr. D Struct. Biol.* **74**, 519–530 (2018). doi:10.1107/S2059798318002425 Medline

69. A. Morin, B. Eisenbraun, J. Key, P. C. Sanschagrin, M. A. Timony, M. Ottaviano, P. Sliz, Collaboration gets the most out of software. *eLife* **2**, e01456 (2013). doi:10.7554/eLife.01456 Medline
